# Questionnaire‐ and linkage‐based outcomes in Dutch childhood cancer survivors: Methodology of the DCCSS LATER study part 1

**DOI:** 10.1002/cam4.5519

**Published:** 2022-12-15

**Authors:** Jop C. Teepen, Judith L. Kok, Elizabeth A. M. Feijen, Jacqueline J. Loonen, Marry M. van den Heuvel‐Eibrink, Helena J. van der Pal, Wim J. E. Tissing, Dorine Bresters, Birgitta Versluys, Martha A. Grootenhuis, Marloes Louwerens, Sebastian J. C. M. M. Neggers, Hanneke M. van Santen, Andrica de Vries, Geert O. Janssens, Jaap G. den Hartogh, Flora E. van Leeuwen, Nynke Hollema, Nina Streefkerk, Ellen Kilsdonk, Margriet van der Heiden‐van der Loo, Eline van Dulmen‐den Broeder, Cécile M. Ronckers, Leontien C. M. Kremer, for the DCCSS LATER Study Group

**Affiliations:** ^1^ Princess Máxima Center for Pediatric Oncology Utrecht The Netherlands; ^2^ Radboudumc Center of Expertise for Cancer Survivorship, Department of Hematology Radboud University Medical Center Nijmegen The Netherlands; ^3^ Department of Pediatric Oncology/Hematology, Erasmus Medical Center Rotterdam The Netherlands; ^4^ Department of Pediatric Oncology/Hematology University of Groningen, University Medical Center Groningen Groningen The Netherlands; ^5^ Department of Internal Medicine Leiden University Medical Center Leiden The Netherlands; ^6^ Department of Medicine, Erasmus Medical Center Rotterdam The Netherlands; ^7^ Department of Pediatric Endocrinology, Wilhelmina Children's Hospital University Medical Center Utrecht Utrecht The Netherlands; ^8^ Department Radiation Oncology, University Medical Center Utrecht Utrecht The Netherlands; ^9^ Dutch Childhood Cancer Organization De Bilt The Netherlands; ^10^ Department of Epidemiology and Biostatistics The Netherlands Cancer Institute Amsterdam The Netherlands; ^11^ Department of Anesthesiology, Intensive Care and Pain Medicine, St. Antonius Hospital Nieuwegein The Netherlands; ^12^ Department of Pediatrics, Erasmus Medical Center Rotterdam The Netherlands; ^13^ Department of Pediatric Oncology/Hematology, Amsterdam UMC Vrije Universiteit Amsterdam Amsterdam The Netherlands; ^14^ Brandenburg Medical School Institute of Biostatistics and Registry Research Neuruppin Germany; ^15^ University Medical Center Utrecht, Wilhelmina Children's Hospital Utrecht The Netherlands; ^16^ Emma Children's Hospital, Amsterdam UMC University of Amsterdam Amsterdam The Netherlands

**Keywords:** childhood cancer survivors, cohort studies, health outcomes, linkage, methodology, surveys and questionnaires

## Abstract

**Background:**

Childhood cancer survivors are at risk for developing long‐term adverse health outcomes. To identify the risk of and risk factors for specific health outcomes, well‐established cohorts are needed with detailed information on childhood cancer diagnosis, treatment, and health outcomes. We describe the design, methodology, characteristics, and data availability of the Dutch Childhood Cancer Survivor Study LATER cohort (1963–2001) part 1; questionnaire and linkage studies.

**Methods:**

The LATER cohort includes 5‐year childhood cancer survivors, diagnosed in the period 1963–2001, and before the age of 18 in any of the seven former pediatric oncology centers in the Netherlands. Information on health outcomes from survivors and invited siblings of survivors was collected by questionnaires and linkages to medical registries.

**Results:**

In total, 6165 survivors were included in the LATER cohort. Extensive data on diagnosis and treatment have been collected. Information on a variety of health outcomes has been ascertained by the LATER questionnaire study and linkages with several registries for subsequent tumors, health care use, and hospitalizations.

**Conclusion:**

Research with data of the LATER cohort will provide new insights into risks of and risk factors for long‐term health outcomes. This can enhance risk stratification for childhood cancer survivors and inform surveillance guidelines and development of interventions to prevent (the impact of) long‐term adverse health outcomes. The data collected will be a solid baseline foundation for future follow‐up studies.

## Graphical abstract

The Dutch Childhood Cancer Survivor Study (DCCSS) LATER cohort includes a large, unselected multi‐center cohort of childhood cancer survivors with an unprecedented combination of detailed, individual‐level data on diagnosis and treatment and highly complete outcome data on various long‐term health outcomes. This Figure gives an overview on inclusion criteria and data collections of part 1 of the DCCSS LATER study.

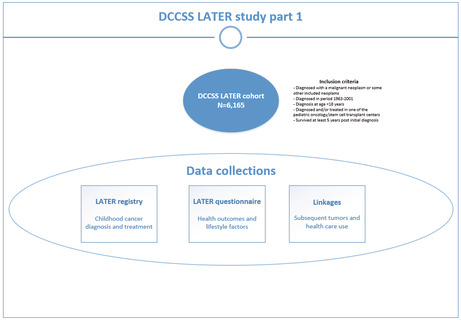

## 1 INTRODUCTION

Although the success of treatment for children with cancer has resulted in a growing population of adult survivors, these survivors are at risk for impairment of long‐term health due to their former disease and its treatment. Previous studies have shown that more than 75% of childhood cancer survivors experience at least one long‐term health problem during their life.^1^, ^2^, ^3^, ^4^ To identify the risk of and risk factors for specific health outcomes, well‐established cohorts are needed with detailed information on childhood cancer diagnosis and treatment as well as validated outcomes of health outcomes.

The Dutch Childhood Cancer Survivor Study (DCCSS) LATER collaborative Study Group was initiated to optimize patient care and research in Dutch childhood cancer survivors. The LATER Study Group aims to (1) coordinate and facilitate multi‐center late effects studies, (2) set up a study cohort of 5‐year survivors, and to (3) design and implement a data platform to facilitate centralized data registration, data monitoring, data handling as well as risk stratification and eligibility checks for specific research studies.

**FIGURE 1:**
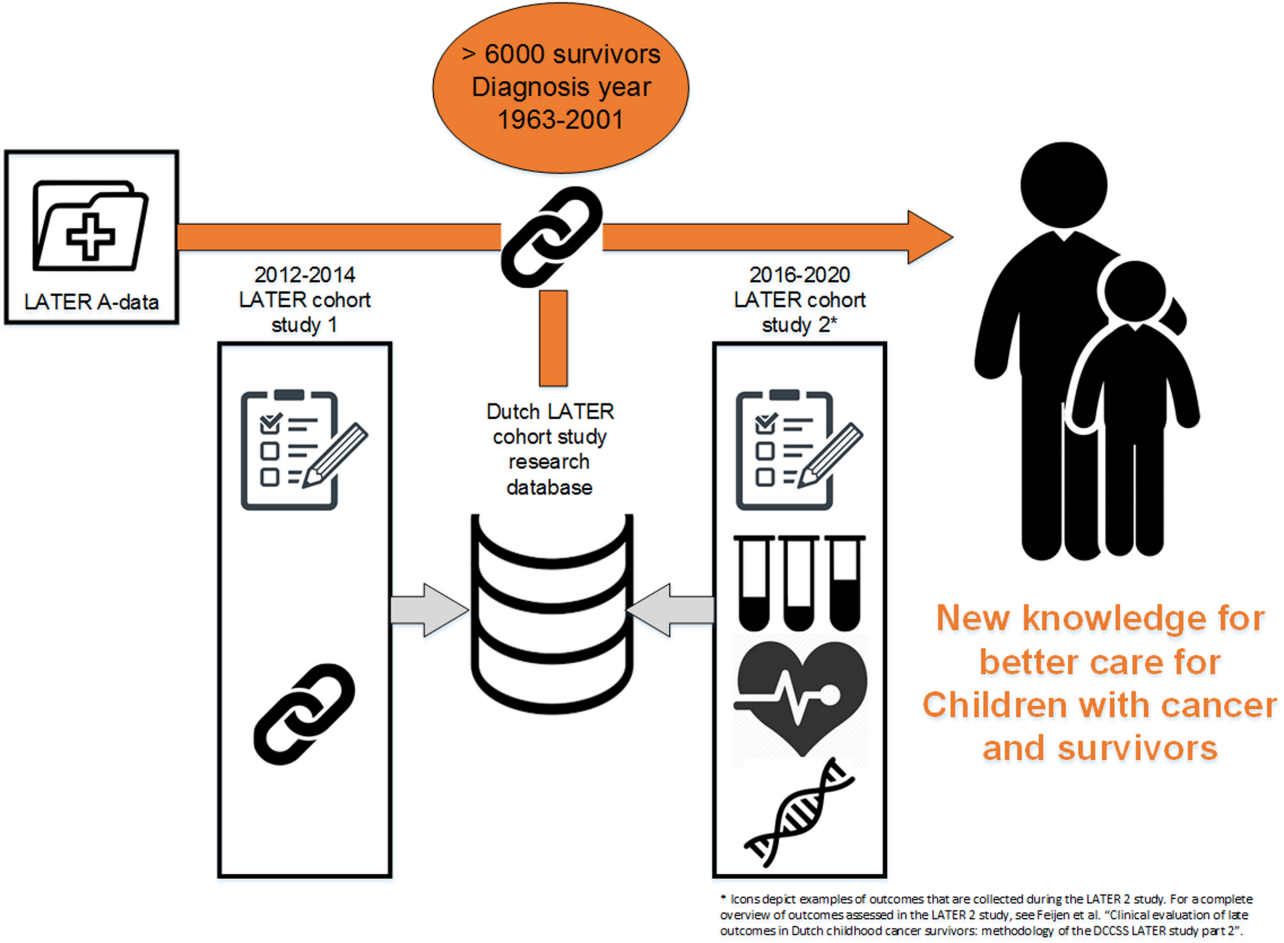
Overview of the DCCSS LATER cohort and specific study parts.

The LATER study consists of two parts (Figure 1). This report concerns the LATER 1 Study, a collection of follow‐up studies with questionnaire and record linkage based outcome assessment for a range of health outcomes and health care use indicators. For the LATER 2 study, survivors received additional study diagnostic tests and study instruments, for example, questionnaires, as part of a regular care visit, as described in more detail elsewhere.

In the LATER 1 study, health outcomes were collected by a general health and lifestyle questionnaire, by linkages to external medical registries, and by reviewing medical records. The goals of the LATER 1 study are (A) to describe risks of long‐term adverse health outcomes among Dutch childhood cancer survivors (B) to identify disease‐, treatment‐, genetic‐, and lifestyle‐related risk factors for long‐term health outcomes among survivors.

The objective of this paper is to describe the design, methodology, and data availability of the LATER study part 1; questionnaire and linkage studies. In addition, we describe its characteristics and unique features.

## 2 METHODS

### 2.1 Study population

The LATER cohort 1963–2001 includes 5‐year childhood cancer survivors who were diagnosed in one of the seven original pediatric oncology/hematopoietic stem cell centers. Eligible patients were identified using existing, prospectively kept patient registries, patient listings, and medical record archives from departments of pediatrics, pediatric oncology and radiation oncology. Completeness of the cohort diagnosed before 1989 has been checked by linkage with trial databases, the Dutch Childhood Oncology Group (DCOG) childhood leukemia registry and regional cancer registries that were running before the start of the nationwide cancer registry. All participating clinics contributed data on survivors diagnosed between 1980 and 2001. For the period prior to 1980, the exact starting year for inclusion in the registry‐cohort is clinic‐specific, depending on the start date of the pediatric (oncology) clinic for those with complete medical file archives or patients listings, or the start date of the local clinic‐specific registry of children with cancer. In addition, if the patient numbers in the first year for a given clinic were very sparse, the starting year was chosen as the first calendar year for which consistent numbers of survivors were identified in view of the distribution of patients over time.

#### 2.1.1 Inclusion criteria LATER cohort

To be eligible for inclusion in the LATER cohort, the following criteria applied: (1) diagnosed with a malignant neoplasm covered by the third edition of the International Classification of Childhood Cancer^5^ and some additional other neoplasms discussed with experts (selected low‐grade brain tumors and systemic multifocal or polyostotic Langerhans cell histiocytosis, as those patients were often treated in pediatric oncology wards in the Netherlands according to protocols that harbor some of the same medications and/or radiotherapy regimens used for malignant conditions (Table S1); (2) diagnosed in the period 1963–2001; (3) diagnosed before age 18 years; (4) diagnosed and/or treated in one of the pediatric oncology/hematopoietic stem cell transplant centers: Amsterdam UMC [the former two hospitals were merged in 2018; Emma Children's Hospital/Academic Medical Center, VU University Medical Center], Willem‐Alexander Children's Hospital/Leiden University Medical Center, Sophia Children's Hospital/Erasmus Medical Center/Daniel den Hoed Clinic Rotterdam, Beatrix Children's Hospital/University Medical Center Groningen, Radboud University Medical Center Nijmegen, and Wilhelmina Children's Hospital/University Medical Center Utrecht); (5) survived at least 5 years post‐initial childhood cancer diagnosis. Enrollment and data collection were done by experienced data managers in the local centers, under supervision of a pediatric oncologist/internal medicine specialist and using structured registration protocols.

### 2.2 Data collection

#### 2.2.1 LATER registry

Data on eligible childhood cancer survivors were entered into the LATER registry based on a LATER pseudonym; the registry does not contain any personally identifiable information. Key lists of LATER pseudonym and patient identifiable information were stored in the pediatric oncology center where the survivors were diagnosed and/or treated for childhood cancer. Since the opening of the Princess Máxima Center for Pediatric Oncology in 2018, many survivors receive late effects outpatient care there and therefore their key list information has been transferred from their original pediatric oncology center to the Princess Máxima Center upon their agreement. Some patients were included in more than one center (because of diagnosis, treatment(s), and/or follow‐up in different centers), leading to administrative twins in the central registry database. Potential duplicates were identified by combinations of birth date, sex, diagnosis year, and childhood cancer type and subsequently verified by the local data managers in the LATER centers, with cross‐validation by phone, based on full name. For the use of health care data and questionnaire data for research purposes, patients (or, in the case of minors, the parents/guardians) were asked for permission during the questionnaire survey. Also, survivors or their legal guardians can at any moment in time indicate that they object to the use of their care data for research purposes, in the pediatric oncology center where they were treated/followed up. Patients who objected to the use of their health care data for research purposes, were registered according to the “minimal registration” protocol, including sex, cancer type, time of diagnosis (in broad categories), and general treatment data (yes/no for surgery, chemotherapy, radiotherapy, respectively). These data are not available for researchers, but can be tabulated in summary to describe the total cohort.

For the web‐based registry details on prior cancer diagnosis and treatment of primary tumor and all recurrences were abstracted by trained data managers in the LATER centers. Treatment information includes details on surgery, radiotherapy, chemotherapy, hematopoietic cell transplantation (HCT), and other supportive medication. Chemotherapy details included start and end dates, drug names, and cumulative doses. In addition, the number of administrations was registered for vincristine. Chemotherapy information was abstracted from chemotherapy charts and other relevant documentation in the medical records. Depending on the unit of chemotherapy dose, height and weight of the child at the time of chemotherapy administration were abstracted to be able to calculate body surface area in cases where dose of chemotherapy was registered as the total dose in mg (absolute value) that needs conversion into dose per m^2^. In addition, names of other drugs and details on HCT were recorded. For radiotherapy, details on prescribed dose, field(s), and boost/surdosage were recorded from the letter of the radiation oncologist in the pediatric oncology main patient file. All data in the LATER registry have been monitored by central LATER data managers on completeness and validity via standardized protocols. Data monitoring consisted of checks for administrative twins, inclusion and exclusion criteria, and on accuracy and completeness of diagnosis and treatment coding.

Vital status and emigration status were initially traced via local resources, including the medical file, recent correspondence with other physicians, the hospital electronic patient information system, and the electronic Personal Records Database, which contains address and vital status information on all inhabitants of the Netherlands from October 1, 1994. In 2014, we linked the cohort to the Central Bureau for Genealogy (which keeps records of Dutch decedents) to update vital status of the cohort up to January 1, 2014. From 2014 onwards, vital status and emigration status were traced via the Personal Records Database, which is accessible in all original treatment centers.

#### 2.2.2 Additional data processing for use in research

##### Chemotherapy

For the chemotherapy data we summed the doses for each agent (per mode of administration, e.g., intravenous, oral, intrathecal) per patient, including the chemotherapy data of primary cancer and all recurrences. In case the dose was in another unit than mg/m^2^, we converted the dose into mg/m^2^ if possible. In total, in 17.8% of all administrations^6^ we knew which agent was administered, but not the exact dose. The number of survivors for whom chemotherapy doses were missing for all administrations was 1%. In cases with missing dose, we imputed the mean dose of other survivors who had the same treatment protocol (if at least three survivors had the same protocol). For survivors without a known protocol, we imputed the mean dose of all survivors within the same diagnosis group and diagnosis period (5‐year period).

##### Radiotherapy

We defined the radiotherapy exposure to different body compartments: head/cranium, spinal, neck, thorax, abdomen/pelvis, extremities, and total body (Figure S1). For each coded radiation field in the LATER registry we assessed whether the body compartment was in‐ or out of primary treatment field. The total prescribed dose (for primary tumor and recurrences) for each body compartment was determined for all individuals, including boost dose, and was summed when the same location was irradiated. We collected the maximum dose to smallest field. In case of two or more non‐overlapping fields in one body compartment, the dose to the field with the highest dose was assigned. The radiotherapy records, such as notes, chart, simulation radiographs, were checked when there was ambiguity. To date, details on radiation charts and simulation films have been collected for 2187 survivors (86% of all survivors treated with radiotherapy). A large‐scale organ dose‐reconstruction effort in collaboration with MD Anderson Cancer Center^7^, ^8^ is ongoing for groups of individuals that have received comparable types of radiotherapy.

#### 2.2.3 LATER questionnaire: health outcomes and lifestyle factors

##### Survivors

Between September 2012 and April 2014, a questionnaire survey was conducted among all 5‐year survivors of the LATER cohort who were alive with a known address in the Netherlands during the survey period, were proficient in Dutch language, and who were not considered ineligible (on active cancer treatment, very poor health status, severe mental health problems, severe mental retardation) to participate by their late effects physician. Eligible survivors received information about the general aims of the study and the central LATER registry. The mailed package consisted of an invitation to participate, including a patient information form, and an informed consent form to allow for central storage of medical data for late effects research purposes. For survivors aged 12–17, parents and the survivors had to sign the informed consent form, while for survivors aged 18+, only the survivor had to sign. The questionnaire inquired about the survivors' medical history after cancer treatment, current disease symptoms, medication use, some aspects of health‐related quality of life, social and psychosexual outcomes, education, nationality and country of birth, socioeconomic status, and lifestyle risk factors for chronic diseases including smoking, alcohol use, and physical activity (see Supplementary Materials for actual questionnaire in Dutch and Supplementary Table 2 for the translation of the items into English). Questionnaire content slightly differed between those aged 12–17 and 18+, and between males and females (e.g., females had additional questions on menarche/menopause, anticonception, and mammograms). For survivors aged 12–17, the parts on sensitive topics (e.g., lifestyle behaviors, such as smoking, and sexual behavior) were in a separate questionnaire, so that survivors could fill these out separate from the questions filled out by their parents. Health outcomes reported in the questionnaire were validated by self‐reported medication use or by medical record abstraction, either from the records of the pediatric oncology or late effects outpatient clinic or from available records from other specialties, if necessary for specific outcomes.

##### Non‐responders: primary health care physician or medical records

For survivors who did not respond to the questionnaire after a written invitation, a written reminder, and at least two telephone reminders, we asked their primary care physician to complete a short questionnaire on major health outcomes and some risk factors. If the primary care physician did not send back the questionnaire after a written invitation, a written reminder, and two telephone reminders, information on major health outcomes and risk factors was extracted from the medical records available in the pediatric oncology department and/or the late effects outpatient clinic medical record. We only used medical records from these departments, because those records usually provide a comprehensive overview on all major health outcomes.

##### Sibling control group

Survivors who participated in the 2013–2014 questionnaire survey were asked to invite their respective siblings. In all, 1662 eligible siblings were reported to the study group, and approached for participation in 2015. They received the same questionnaire as the survivors, except for specific questions related to the previous cancer. Health outcomes for siblings were validated by self‐reported medication use or by contacting their primary care physician.

#### 2.2.4 Linkages

Linkages of the LATER cohort with external medical registries were performed, including the population‐based Netherlands Cancer Registry (NCR) to identify subsequent malignant neoplasms (SMNs). The NCR has nationwide coverage since 1989.^9^ Furthermore, the cohort was linked to the nationwide network and registry of histo‐ and cytopathology in the Netherlands (PALGA) to obtain information on solid benign tumors, basal cell carcinomas, and on SMNs in the period <1989.^10^ PALGA was established in 1970 and attained full nationwide coverage in 1991. Information on primary care physician‐based health care use and hospitalizations was ascertained by linkage with the Nivel Primary Care database and with the Dutch Hospital Discharge register and compared with matched general population controls.

#### 2.2.5 Published studies

We summarized the study characteristics and outcomes of all studies that used data of the LATER 1 study. In parallel to the LATER collaboration, an outcome‐specific epidemiologic LATER cohort study on female reproductive health outcomes among cancer survivors was set‐up; details on methodology of the DCCSS‐LATER‐VEVO effort were reported previously.^11^, ^12^, ^13^

## 3 RESULTS

### 3.1 LATER cohort 1963–2001

Table 1 shows tumor‐ and treatment characteristics of 6165 survivors in the LATER cohort diagnosed during 1963–2001, as well as the subgroups who participated in the questionnaire study, the non‐participants, and the non‐eligible survivors. There were slightly more male survivors (55.7%) in the cohort. The most common primary cancer diagnoses were leukemias (34.0%), followed by lymphomas (17.2%), and central nervous system (CNS) tumors (13.7%). More than half of the survivors were diagnosed in the period 1990–2001 (50.9%). Of all survivors, 48.1% received chemotherapy without radiotherapy, 32.9% chemotherapy with radiotherapy, 7.9% received radiotherapy without chemotherapy, 9.3% surgery only, and 1.0% no treatment. Among survivors treated with radiotherapy, head/cranium was the most common site (22.8%), followed by abdomen/pelvis (7.7%), and spine (7.2%). The most common group of chemotherapy exposure was vinca alkaloids (72.6%), while about half of all survivors received alkylating agents (51.5%), antimetabolites (47.1%), and anthracyclines (45.6%). In the cohort, 6.4% had received an HCT.

**TABLE 1:** Characteristics of the DCCSS LATER cohort

Characteristics	Overall		Questionnaire participants		Questionnaire non‐participants		Non‐eligible	
	(*N* = 6165)		(*N* = 3369)		(*N* = 1958)		(*n* = 838)	
	*n*	%	*n*	%	*n*	%	*n*	%
Sex								
Female	2731	44.3%	1609	47.8%	760	38.8%	362	43.2%
Male	3434	55.7%	1760	52.2%	1198	61.2%	476	56.8%
Transgender	1	0.0%	0	0.0%	1	0.0%	0	
Primary childhood cancer diagnosis^a^								
Leukemias, myeloproliferative diseases and myelodysplastic diseases	2094	34.0%	1157	34.3%	675	34.5%	262	31.3%
Lymphomas and reticulo endothelial neoplasms	1062	17.2%	587	17.4%	358	18.3%	117	14.0%
CNS and miscellaneous intracranial and intraspinal neoplasms	844	13.7%	433	12.9%	249	12.7%	162	19.3%
Neuroblastoma and other peripheral nervous cell tumors	324	5.3%	179	5.3%	108	5.5%	37	4.4%
Retinoblastoma	33	0.5%	15	0.4%	13	0.7%	5	0.6%
Renal tumors	596	9.7%	371	11.0%	181	9.2%	44	5.3%
Hepatic tumors	52	0.8%	35	1.0%	15	0.8%	2	0.2%
Bone tumors	370	6.0%	184	5.5%	103	5.3%	83	9.9%
Soft tissue and other extraosseous sarcomas	450	7.3%	235	7.0%	139	7.1%	76	9.1%
Germ cell tumors, trophoblastic tumors, and neoplasms of gonads	232	3.8%	124	3.7%	81	4.1%	27	3.2%
Other malignant epithelial neoplasms and malignant melanomas	102	1.7%	45	1.3%	35	1.8%	22	2.6%
Other and unspecified malignant neoplasms	6	0.1%	4	0.1%	1	0.1%	1	0.1%
Age at diagnosis^b^								
0–4 y	2727	45.3%	1579	46.9%	831	45.3%	317	38.9%
5–9 y	1628	27.1%	896	26.6%	506	27.6%	226	27.8%
10–14 y	1285	21.4%	690	20.5%	395	21.5%	200	24.6%
15–17 y	376	6.3%	202	6.0%	103	5.6%	71	8.7%
Calendar period of childhood cancer diagnosis								
1963–1969	119	1.9%	59	1.8%	21	1.1%	39	4.7%
1970–1979	978	15.9%	493	14.6%	236	12.1%	249	29.7%
1980–1989	1931	31.3%	1048	31.1%	589	30.1%	294	35.1%
1990–2001	3137	50.9%	1769	52.5%	1112	56.8%	256	30.5%
Time since childhood cancer diagnosis at last known date^c^								
5–9 y	338	5.5%	0	0.0%	0	0.0%	338	40.3%
10–19 y	1276	20.7%	636	18.9%	429	21.9%	211	25.2%
20–29 y	2301	37.3%	1320	39.2%	836	42.7%	145	17.3%
30+ y	2250	36.5%	1413	41.9%	693	35.4%	144	17.2%
Attained age at last known date^b^ ^,^ ^c^								
<20 y	562	9.3%	135	4.0%	64	3.5%	363	44.6%
20–29 y	1745	29.0%	982	29.2%	562	30.6%	201	24.7%
30–39 y	2051	34.1%	1182	35.1%	737	40.2%	132	16.2%
40+ y	1658	27.6%	1068	31.7%	472	25.7%	118	14.5%
Vital status at last known date^c^								
Alive	5428	88.0%	3283	97.4%	1920	98.1%	225	26.8%
Dead	737	12.0%	86	2.6%	38	1.9%	613	73.2%
Chemotherapy^d^								
No	1123	18.2%	1624	48.2%	952	48.6%	336	40.1%
Yes	5005	81.2%	1729	51.3%	994	50.8%	462	55.1%
Missing	37	0.6%	16	0.5%	12	0.6%	40	4.8%
Radiotherapy^d^								
No	3608	58.5%	2045	60.7%	1296	66.2%	267	31.9%
Yes	2527	41.0%	1319	39.2%	658	33.6%	550	65.6%
Missing	30	0.5%	5	0.1%	4	0.2%	21	2.5%
Surgery^d^								
No	2912	47.2%	1624	48.2%	952	48.6%	336	40.1%
Yes	3185	51.7%	1729	51.3%	994	50.8%	462	55.1%
Missing	68	1.1%	16	0.5%	12	0.6%	40	4.8%
Therapy^d^								
No treatment	61	1.0%	23	0.7%	29	1.5%	9	1.1%
Surgery only	575	9.3%	295	8.8%	233	11.9%	47	5.6%
Chemotherapy only (± surgery)	2967	48.1%	1727	51.3%	1033	52.8%	207	24.7%
Radiotherapy only (± surgery)	484	7.9%	251	7.5%	125	6.4%	108	12.9%
Chemotherapy and radiotherapy (± surgery)	2030	32.9%	1067	31.7%	533	27.2%	430	51.3%
Missing	48	0.8%	6	0.2%	5	0.3%	37	4.4%
Radiotherapy site^b^ ^,^ ^d^ ^,^ ^e^								
Head/cranium	1359	22.8%	720	21.5%	314	17.2%	325	41.2%
Spinal	430	7.2%	207	6.2%	73	4.0%	150	19.1%
Neck	235	3.9%	117	3.5%	73	4.0%	45	5.7%
Thorax	390	6.5%	199	5.9%	104	5.7%	87	11.1%
Abdomen/pelvis	460	7.7%	271	8.1%	104	5.7%	85	10.8%
Extremities	131	2.2%	66	2.0%	29	1.6%	36	4.6%
Total body irradiation	218	3.7%	105	3.1%	55	3.0%	58	7.4%
Chemotherapy^b^ ^,^ ^d^								
Alkylating agents	3074	51.5%	1718	51.1%	900	49.2%	456	58.4%
Anthracyclines	2722	45.6%	1539	45.8%	819	44.7%	364	46.7%
Epipodophyllotoxins	1282	21.5%	657	19.6%	372	20.3%	253	32.5%
Vinca alkaloids	4335	72.6%	2490	74.1%	1307	71.4%	538	68.7%
Platinum agents	786	13.2%	421	12.5%	219	12.0%	146	18.8%
Antimetabolites	2813	47.1%	1601	47.6%	861	47.0%	351	44.9%
Hematopoietic cell transplantation^b^ ^,^ ^d^								
No	5532	92.0%	3156	93.7%	1731	94.3%	645	79.2%
Autologous bone marrow transplant	155	2.6%	71	2.1%	28	1.5%	56	6.9%
Allogenic bone marrow transplant	231	3.8%	119	3.5%	62	3.4%	50	6.1%
Missing	98	1.6%	21	0.6%	14	0.8%	63	7.7%

^a^ Diagnostic groups included all malignancies covered by the third edition of the International Classification of Childhood Cancer (ICCC‐3) as well as multifocal Langerhans Cell Histiocytosis, and selected non‐malignant ependymomas and astrocytomas.

^b^ Information was missing for all survivors who declined registration (*n* = 149). Information on radiotherapy site variables and chemotherapy groups were missing for an additional 41–61 survivors (depending on variable). Percentages were calculated based on cohort with information.

^c^ Information was complete for 91.8% up to January 1st 2017.

^d^ Treatment data includes primary treatment and treatment for recurrences.

^e^ Radiotherapy includes external beam radiotherapy, brachytherapy, and radioisotopes.

Table 2 presents treatment characteristics by childhood cancer type. By childhood cancer types, CNS tumor survivors had the highest percentage of treatment with surgery only (32.3%) as well as radiotherapy without chemotherapy (30.5%). More than half of CNS tumor survivors (56.9%) and 28% of leukemia survivors received radiotherapy to the head/cranium. Highest percentages of alkylating agent treatment were among survivors of lymphomas (81.6%) and soft tissue sarcomas (74.4%) and treatment with anthracyclines was most common among survivors of bone tumors (83.1%) and lymphomas (68.1%). Allogenic HCT occurred almost exclusively among survivors of leukemias and lymphomas, while autologous HCT also occurred infrequently among other childhood cancer types.

**TABLE 2:** Treatment characteristics by childhood cancer diagnosis

Characteristics	Primary childhood cancer diagnosis^a^													
	Leukemias		Lymphomas		CNS tumors		Renal tumors		Bone tumors		Soft tissue sarcomas		Other neoplasms	
	(*N* = 2094)		(*N* = 1062)		(*N* = 844)		(*N* = 596)		(*N* = 370)		(*N* = 450)		(*N* = 749)	
	*n*	%	*n*	%	*n*	%	*n*	%	*n*	%	*n*	%	*n*	%
Therapy^c^														
No treatment	7	0.3%	9	0.8%	29	3.4%	1	0.2%	0	0.0%	0	0.0%	15	1.9%
Surgery only	2	0.1%	4	0.4%	273	32.3%	15	2.5%	17	4.6%	51	11.3%	213	26.8%
Chemotherapy only (±surgery)	1291	61.7%	632	59.5%	45	5.3%	336	56.4%	194	52.4%	192	42.7%	277	34.9%
Radiotherapy only (±surgery)	1	0.0%	47	4.4%	257	30.5%	9	1.5%	31	8.4%	26	5.8%	113	14.2%
Chemotherapy and radiotherapy (±surgery)	773	36.9%	362	34.1%	229	27.1%	234	39.3%	126	34.1%	178	39.6%	128	16.1%
Missing	20	1.0%	8	0.8%	11	1.3%	1	0.2%	2	0.5%	3	0.7%	3	0.4%
Radiotherapy site^b^ ^,^ ^c^ ^,^ ^d^														
Head/cranium	568	28.0%	124	12.0%	452	56.9%	2	0.3%	16	4.4%	98	22.3%	99	13.6%
Spinal	114	5.6%	25	2.4%	224	28.3%	5	0.9%	19	5.2%	6	1.4%	37	5.1%
Neck	8	0.4%	176	17.1%	1	0.1%	2	0.3%	0	0.0%	25	5.7%	23	3.2%
Thorax	18	0.9%	211	20.5%	0	0.0%	44	7.5%	71	19.6%	23	5.2%	23	3.2%
Abdomen/pelvis	12	0.6%	99	9.6%	0	0.0%	225	38.5%	16	4.4%	48	10.9%	60	8.2%
Extremities	2	0.1%	28	2.7%	1	0.1%	2	0.3%	69	19.2%	25	5.7%	4	0.5%
Total body irradiation	204	10.1%	14	1.4%	0	0.0%	0	0.0%	0	0.0%	0	0.0%	0	0.0%
Chemotherapy^b^ ^,^ ^c^														
Alkylating agents	1124	55.5%	848	81.6%	202	25.4%	58	9.9%	235	64.9%	326	74.4%	281	38.4%
Anthracyclines	1176	58.1%	708	68.1%	9	1.1%	199	34.1%	301	83.1%	192	43.8%	137	18.7%
Epipodophyllotoxins	519	25.7%	254	24.4%	103	13.0%	48	8.2%	55	15.2%	91	20.7%	212	29.0%
Vinca alkaloids	1895	93.5%	947	91.1%	232	29.2%	524	89.9%	196	54.1%	351	80.0%	190	26.0%
Platinum agents	3	0.1%	36	3.5%	178	22.4%	48	8.2%	140	38.7%	100	22.8%	281	38.4%
Antimetabolites	2005	98.9%	562	54.1%	80	10.1%	4	0.7%	104	28.7%	21	4.8%	37	5.1%
Hematopoietic cell transplantation^b^ ^,^ ^c^														
No	1724	84.4%	995	95.1%	779	97.0%	574	98.3%	347	95.3%	424	95.9%	689	93.9%
Autologous	66	3.2%	27	2.6%	5	0.6%	5	0.9%	8	2.2%	9	2.0%	35	4.8%
Allogenic	218	10.7%	12	1.1%	0	0.0%	0	0.0%	0	0.0%	0	0.0%	1	0.1%
Missing	35	1.7%	12	1.1%	19	2.4%	5	0.9%	9	2.5%	9	2.0%	9	1.2%

^a^ Diagnostic groups included all malignancies covered by the third edition of the International Classification of Childhood Cancer (ICCC‐3) as well as multifocal Langerhans Cell Histiocytosis, and selected non‐malignant ependymomas and astrocytomas.

^b^ Information was missing for all survivors who declined registration (*n* = 149). Information on radiotherapy site variables and chemotherapy groups were missing for an additional 41–61 survivors. (depending on variable). Percentages were calculated based on cohort with information.

^c^ Treatment data includes primary treatment and treatment for recurrences.

^d^ Radiotherapy includes external beam radiotherapy, brachytherapy, and radioisotopes.

**FIGURE 2:**
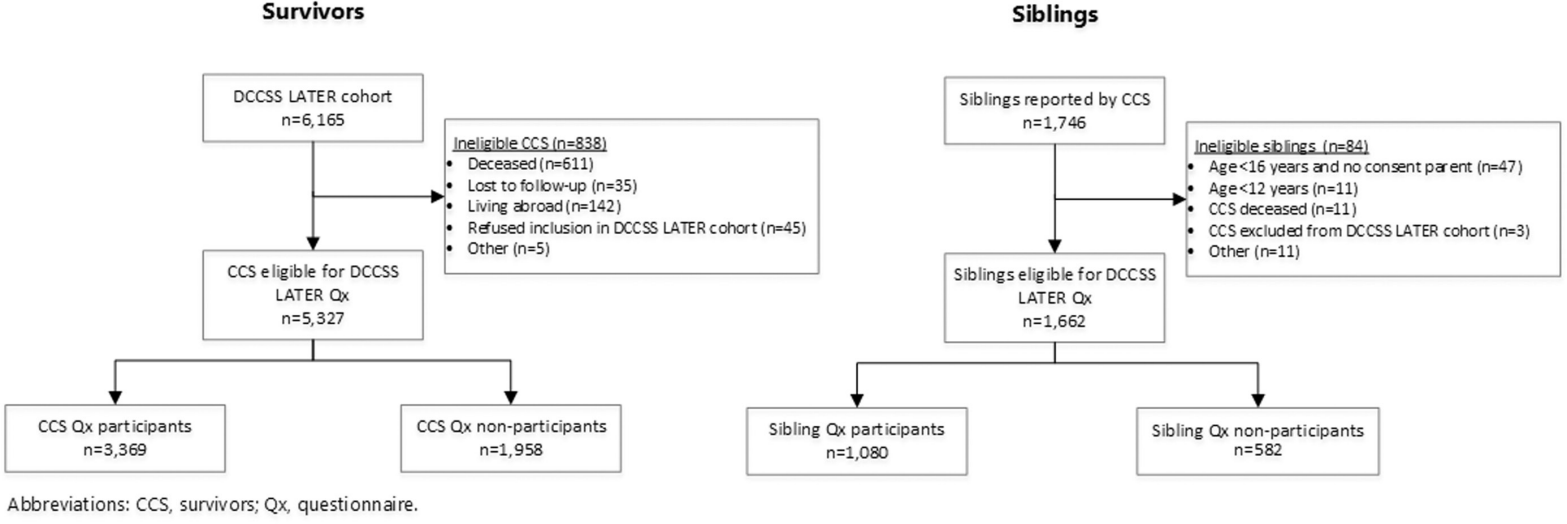
Flowchart of invitation process and participation of survivors and siblings in the DCCSS LATER questionnaire.

In total, 5327 survivors were invited for the questionnaire survey, of whom 3369 (63.2%) participated (Figure 2). For siblings, 1662 were invited and 1080 (65.0%) participated.

### 3.2 Published studies

Until present, 35 published reports from national and international collaborative studies include data from the LATER 1 study,^6^, ^14^, ^15^, ^16^, ^17^, ^18^, ^19^, ^20^, ^21^, ^22^, ^23^, ^24^, ^25^, ^26^, ^27^, ^28^, ^29^, ^30^, ^31^, ^32^, ^33^, ^34^, ^35^, ^36^, ^37^, ^38^, ^39^, ^40^, ^41^, ^42^, ^43^, ^44^ addressing cardiac outcomes,^20^, ^21^, ^22^, ^23^ subsequent tumors,^6^, ^14^, ^15^, ^19^, ^24^, ^25^, ^26^, ^29^, ^33^, ^34^, ^35^, ^42^, ^44^, ^45^ burden of disease,^31^, ^32^ mortality,^46^ fatigue,^47^ ototoxicity,^16^, ^17^, ^18^, ^27^, ^28^ reproductive outcomes,^30^, ^36^, ^37^, ^38^, ^39^, ^40^ and psychosocial outcomes.^41^, ^43^ Characteristics of published studies are summarized in Table S3.

## 4 DISCUSSION

The LATER cohort study includes a large, unselected multi‐center cohort of childhood cancer survivors with an unprecedented combination of detailed, individual‐level data on diagnosis and treatment and highly complete outcome data on various long‐term health outcomes. In this paper, we presented an overview of methods of cohort identification and data collection, of baseline characteristics of the cohort, and of published papers with data from the cohort.

The LATER cohort is unique as it represents a large near‐national cohort with detailed individual data on childhood cancer diagnosis and treatment for the majority of childhood cancer survivors in the Netherlands initially diagnosed in the period 1963–2001, including treatment data on recurrences and subsequent tumors. A small group of patients who were treated outside the pediatric oncology centers are not covered, such as 15–17‐year‐old lymphoma patients treated in hematology departments, thyroid cancer patients treated by endocrinologists/adult oncologists, retinoblastoma patients treated by ophthalmologists, and certain types of brain tumors treated by neurosurgeons. This is an advantage compared to hospital‐based cohorts, as hospital‐based cohorts might not reflect the total eligible cohort of childhood cancer survivors with respect to the distribution of childhood cancer types and disease severity and therefore might underestimate or overestimate the population risks of late effects. The LATER study does not rely solely on self‐reported information from questionnaires. Self‐reported information on health outcomes was validated by self‐reported medication use or by medical records to limit information bias. For non‐responders, information on key outcomes was obtained via their primary care physicians. Also, some health outcomes, such as subsequent tumors, hospital episodes, and primary care physician use, were obtained by linkages to nationwide disease registries and health care registrations available for secondary data use. This provided objective and complete information on these outcomes from the time those registrations were started. Also, for several outcomes we also had a control group of siblings of survivors, as they were also invited to participate in the questionnaire study. Because data have been collected for a large and heterogeneous group of childhood cancer survivors, the LATER study also provides unique opportunities to compare health outcomes across certain subgroups of survivors.

A limitation of the study is that more than one third (36.8%) of eligible survivors who received the questionnaire survey did not participate and therefore no extensive questionnaire data are available for these survivors. Although this response rate is fairly high for a questionnaire study, this could possibly under‐ or overestimate the true incidence of health outcome and the effects of risk factors. However, we did not observe major differences in childhood cancer types and treatments between participants and nonparticipants, so we do not expect this to be a major influence, although we cannot exclude the possibility that for some specific subgroups of survivors this might impact results slightly. Also, for nonparticipants we collected information on key health outcomes via their GP or medical records and those survivors were included in linkage‐based health outcome ascertainment, so data on those outcomes were largely complete. Another thing to take into consideration when interpreting results of the DCCSS LATER cohort is that there can be differences with cohorts from other countries with respect to treatment approach, general culture (such as lifestyle factors), and health care system.

There are several other large childhood cancer survivors cohort studies worldwide. Large cohorts in North America include the hospital‐based Childhood Cancer Survivor Study^48^ and the St. Jude Lifetime Cohort Study,^49^ as well as the population‐based Childhood, Adolescent and Young Adult Cancer Survivors Program.^50^ In Europe, there are the population‐based British Childhood Cancer Survivor Study,^51^ the Adult Life after Childhood Cancer in Scandinavia,^52^ and the Swiss Childhood Cancer Survivor Study,^53^ and the hospital‐based French Childhood Cancer Survivor Study,^54^ French childhood cancer survivor study for leukemia (LEA Cohort),^55^ and the Italian Study on off‐therapy Childhood Cancer Survivors.^56^ To get more insight in rare outcomes it will be very important to collaborate internationally by combining data from these large‐scale cohorts with long‐term follow‐up data on outcomes. Examples of such collaboration are the PanCareSurFup consortium, in which data from 13 data providers in 12 European countries were collected and harmonized to study cardiac outcomes, second cancers, and mortality in European childhood cancer survivors^57^ and The International Consortium for Pooled Studies on Subsequent Malignancies after Childhood and Adolescent Cancer, a consortium initiated by the Princess Máxima Center to initially pool data from seven cohorts worldwide in order to answer clinically‐relevant questions on breast cancer risk after childhood cancer.^58^

The data collected will be a solid baseline foundation for new studies. It is necessary to continue the follow‐up of the cohort, as chronic health outcomes will increase as the population ages. Also, it is important to study long‐term health outcomes in more recently diagnosed survivors (2002 and later), as these survivors may have different risk patterns, because treatments have been evolved. At the moment we have already collected data on diagnosis and treatment for more than 14,000 survivors diagnosed until 2015. In the future, research results will also be translated into intervention studies. For example, currently a lifestyle intervention is ongoing among survivors with obesity and/or low physical activity who aim to improve their lifestyle.

In conclusion, we described the design, methodology, characteristics, and data availability of the Dutch Childhood Cancer Survivor Study LATER cohort (1963–2001) part 1; questionnaire and linkage studies. Research including data collected for the LATER cohort will provide new insights into risks of and risk factors for long‐term health outcomes, which can enhance risk stratification for childhood cancer survivors and inform surveillance guidelines and development of interventions to prevent (the impact of) long‐term adverse health outcomes. The data collected will be a solid baseline foundation for future follow‐up studies.

## AUTHOR CONTRIBUTIONS

**Judith L. Kok:** Formal analysis (equal); investigation (equal); methodology (equal); writing – original draft (equal); writing – review and editing (equal). **Elizabeth (Lieke) A.M. Feijen:** Methodology (equal); writing – review and editing (equal). **J.J. Loonen:** Conceptualization (equal); funding acquisition (equal); methodology (equal); resources (equal); writing – review and editing (equal). **Marry M. van den Heuvel‐Eibrink:** Conceptualization (equal); funding acquisition (equal); methodology (equal); resources (equal); writing – review and editing (equal). **Helena J.H. van der Pal:** Conceptualization (equal); funding acquisition (equal); methodology (equal); resources (equal); writing – review and editing (equal). **Wim J.E. Tissing:** Conceptualization (equal); funding acquisition (equal); methodology (equal); resources (equal); writing – review and editing (equal). **Dorine Bresters:** Conceptualization (equal); funding acquisition (equal); methodology (equal); resources (equal); writing – review and editing (equal). **A. Birgitta Versluys:** Conceptualization (equal); funding acquisition (equal); methodology (equal); resources (equal); writing – review and editing (equal). **Martha A. Grootenhuis:** Conceptualization (equal); methodology (equal); writing – review and editing (equal). **Marloes Louwerens:** Methodology (equal); writing – review and editing (equal). **Sebastian Neggers:** Conceptualization (equal); methodology (equal); writing – review and editing (equal). **H.M. van Santen:** Methodology (equal); writing – review and editing (equal). **Andrica de Vries:** Methodology (equal); writing – review and editing (equal). **Geert O.R. Janssens:** Methodology (equal); writing – review and editing (equal). **Jaap G. den Hartogh:** Conceptualization (equal); methodology (equal); writing – review and editing (equal). **Flora E. van Leeuwen:** Conceptualization (equal); methodology (equal); writing – review and editing (equal). **Nynke Hollema:** Methodology (equal); writing – review and editing (equal). **Nina Streefkerk:** Methodology (equal); writing – review and editing (equal). **Ellen Kilsdonk:** Methodology (equal); writing – review and editing (equal). **Margriet van der Heiden‐van der Loo:** Formal analysis (equal); investigation (equal); methodology (equal); supervision (equal); writing – review and editing (equal). **Eline van Dulmen‐den Broeder:** Conceptualization (equal); funding acquisition (equal); methodology (equal); resources (equal); writing – review and editing (equal). **Cecile M. Ronckers:** Conceptualization (equal); formal analysis (equal); funding acquisition (equal); investigation (equal); methodology (equal); writing – review and editing (equal). **Leontien C. M. Kremer:** Conceptualization (equal); formal analysis (equal); funding acquisition (equal); investigation (equal); methodology (equal); resources (equal); supervision (equal); writing – review and editing (equal)

## FUNDING INFORMATION

The DCCSS LATER 1 study was funded by Quality of Life GALA foundation (DCOG‐LATER Q2008 Childhood Cancer Survivor Studies, 2008–2013), Dutch Cancer Society/KiKa (SKION LATER registry. 2006–2010), Dutch Cancer Society (Grant No. DCOG2011‐5027 and UVA2012‐5517), European Union's Seventh Framework Programme for research, technological development and demonstration (Grant Agreement No. 257505; PanCareSurFup), and a PhD grant awarded by the Academic Medical Center Executive Board to Drs Kremer and Ronckers.

## CONFLICT OF INTEREST

The authors have declared no conflict of interest.

## ETHICS APPROVAL STATEMENT

The study protocol was declared exempt from review of medical intervention research by the institutional review boards of participating centers, because the survivors were not subjected to follow rules of behavior and the study was considered to not impact the physical and/or psychological integrity of the subjects, in compliance with Dutch law and regulations for health research involving human beings.

## PATIENT CONSENT STATEMENT

In total, 149 survivors declined registration in the DCCSS LATER registry and are not included in the studies. All questionnaire participants gave written informed consent for use of their data. For selected retrospective data collections and linkages, specific consent was not needed in accordance with Dutch legislation.

## Supporting information


Appendix S1

Figure S1

Table S1–S3

## Data Availability

For data and material request, an email can be addressed to the corresponding author.
