# Supplementary material for: Questionnaire‐ and linkage‐based outcomes in Dutch childhood cancer survivors: Methodology of the DCCSS LATER study part 1
Source: Cancer Med. 2022 Dec 15;12(6):7588–602. doi: 10.1002/cam4.5519 (PMC10067029; doi:10.1002/cam4.5519)
Supplement: Supplementary file 1 — Appendix S1 [file CAM4-12-7588-s002.doc]

**Supplementary Materials**

Different versions of the LATER questionnaire on medical history after cancer treatment, current disease symptoms, medication use, social and psychosexual outcomes, education, socioeconomic status, and lifestyle risk factors for chronic diseases including smoking, alcohol use, and physical activity (in Dutch)

- Pages 2-17: Questionnaire girls aged 12-17
- Pages 18-32: Questionnaire boys aged 12-17
- Pages 33-52: Questionnaire parents of girls and boys aged 12-17
- Pages 53-83 Questionnaire females aged 18+
- Pages 84-112: Questionnaire males aged 18+

**
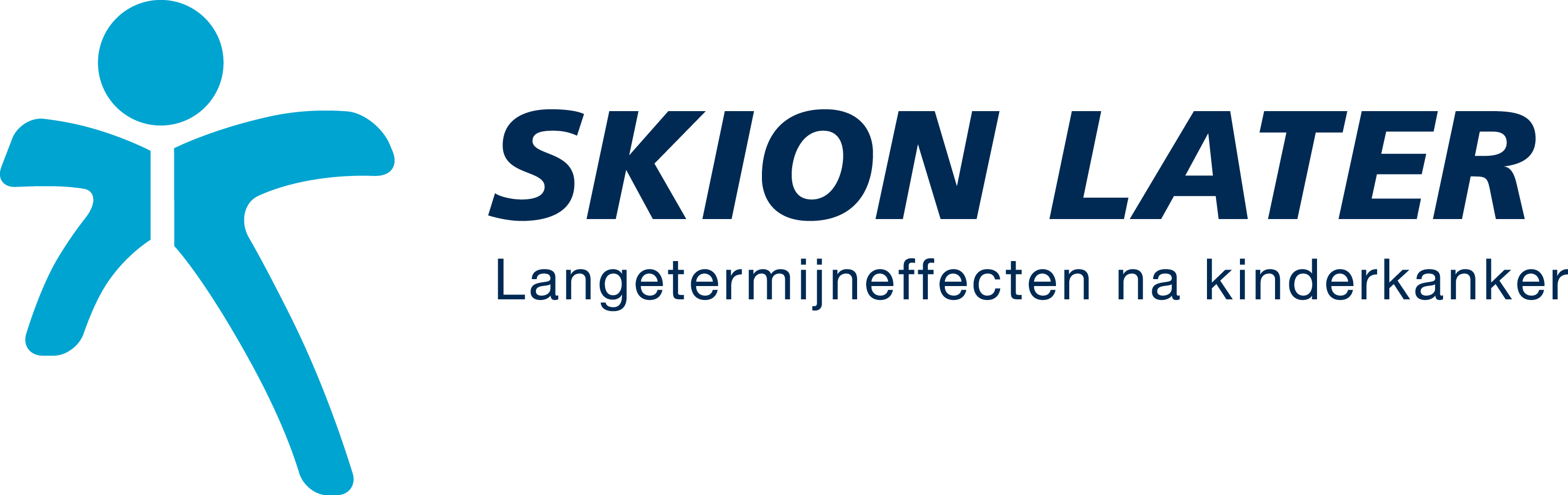
**

LATER VRAGENLIJST voor MEISJES 12 t/m 17 jr

Versie A1 MEISJES 12tm17 papier 2013 03 20

**VRAGENLIJST**

| LATER nummer  Datum invullen vragenlijst: | …………………………..……….…………..  …………………………..……….………….. |
| --- | --- |

**TOELICHTING BIJ HET INVULLEN VAN DE VRAGENLIJST**

- Het is de bedoeling dat deze vragenlijst ingevuld wordt door de persoon aan wie de vragenlijst is gestuurd; aan jou dus! Het gaat om je ***eigen*** antwoorden. Wanneer je de vragen zelf niet kunt lezen of invullen, kan iemand anders je helpen met het invullen van de vragen. Je moet wel zelf de antwoorden geven.
- Wij verzoeken je zoveel mogelijk alle vragen te beantwoorden. Soms kun je vragen overslaan, dit wordt dan duidelijk aangegeven bij de vraag. Kruis per vraag één antwoord aan. Als er meerdere antwoorden mogelijk zijn, dan wordt dat bij elke vraag apart aangegeven. Als je het antwoord op een vraag niet meer precies weet, bijvoorbeeld een leeftijd of een datum, probeer dan een zo goed mogelijke ***schatting*** te geven.
- Het kan voorkomen dat je in deze vragenlijst om (medische) informatie wordt gevraagd die jouw behandelend arts al heeft, maar de onderzoeker nog niet. Daarom hebben we deze vragen nogmaals opgenomen in de vragenlijst.
- Als je bij een vraag te weinig ruimte hebt kun je de rest van het antwoord vermelden bij het opmerkingen veld, op de laatste bladzijde. Schrijf dan alsjeblieft wel het nummer van de vraag op, waar het antwoord bij hoort.
- Probeer zoveel mogelijk binnen het hokje te blijven. Hieronder volgen een aantal voorbeelden van verschillende vragen.

Bij sommige vragen word je gevraagd het juiste hokje aan te kruisen:

Voorbeeld: Heeft een week 7 dagen? 🗷 ja

🞏 nee

Als je een antwoord wilt herstellen, kun je op de volgende manier voor het juiste antwoord een pijltje zetten:

Voorbeeld: Heeft een week 7 dagen? 🗷 ja

🗷 nee

Bij andere vragen is het de bedoeling dat je iets invult:

Voorbeeld: Hoeveel maanden heeft een jaar? ***12*** maanden

- Wij willen je nogmaals laten weten dat alle gegevens die je invult strikt vertrouwelijk behandeld worden. De gegevens worden opgeslagen met een code, dus zonder jouw naam en adres.

**Je kunt nu beginnen met de vragenlijst en we willen je nu vast bedanken voor het invullen !**

| **ALGEMEEN** | |
| --- | --- |
| 1. Op welke datum ben je geboren ? | ….… - …... - ….……. |
| 1. Wat is de geboorteplaats ? | geboorteplaats: ………………………………………………………… |
| 1. Ligt jouw geboorteplaats in Nederland? | - ja - nee |
| 1. Hoeveel broers en zussen heb je ?  *Halfzussen en halfbroers tellen niet mee.* | …... broers  …... zussen   - weet ik niet |
| 1. Zit je op school? | - ja - nee 🡺 *ga door met vraag A7* |
| 1. Zo ja, wat voor soort school? | - regulier basisonderwijs (lagere school) - speciaal basisonderwijs - voortgezet speciaal onderwijs - praktijkonderwijs - VMBO - middelbaar beroepsonderwijs (MBO) - HAVO - VWO, Gymnasium - hoger beroepsonderwijs (HBO) - anders, namelijk …………………………………..…………………. |
| 1. Wat wil je later worden? | …………………………………………………………………………… |

| **LICHAMELIJKE ACTIVITEITEN** |
| --- |
| **De volgende vragen gaan over dagelijkse bezigheden.**   1. Word je door je gezondheid de **afgelopen 4 weken** beperkt bij deze bezigheden. Zo ja, in welke mate?  |  | **Ja, heel erg beperkt** | **Ja, een beetje beperkt** | **Nee, helemaal niet beperkt** | | --- | --- | --- | --- | | - 1. **Forse inspanning**   (zoals hardlopen, zware voorwerpen tillen, inspannend sporten) | 🞎 | 🞎 | 🞎 | | - 1. **Matige inspanning**   (zoals het verplaatsen van een tafel, stofzuigen, fietsen) | 🞎 | 🞎 | 🞎 | | - 1. Tillen of boodschappentas dragen | 🞎 | 🞎 | 🞎 | | - 1. **Een paar** trappen oplopen | 🞎 | 🞎 | 🞎 | | - 1. **Eén** trap oplopen | 🞎 | 🞎 | 🞎 | | - 1. Buigen, knielen, of bukken | 🞎 | 🞎 | 🞎 | | - 1. **Meer dan een kilometer** lopen | 🞎 | 🞎 | 🞎 | | - 1. **Een halve kilometer** lopen | 🞎 | 🞎 | 🞎 | | - 1. **Honderd meter** lopen | 🞎 | 🞎 | 🞎 | | - 1. Jezelf wassen en aankleden | 🞎 | 🞎 | 🞎 | |

| 1. In hoeverre heeft je lichamelijke gezondheid of hebben emotionele problemen je de **afgelopen 4 weken** belemmerd in je normale sociale bezigheden met gezin, vrienden, buren of anderen? | - helemaal niet - een beetje - nogal - veel - heel erg veel |
| --- | --- |
| 1. **Hoe vaak** hebben je lichamelijke gezondheid of emotionele problemen gedurende de **afgelopen 4 weken** je sociale activiteiten (zoals bezoek aan vrienden of uitgaan) belemmerd? | - voortdurend - meestal - soms - zelden - nooit |
| 1. Hoeveel pijn had je de **afgelopen 4 weken**? | - geen - heel licht - licht - nogal - ernstig - heel ernstig |
| 1. In welke mate heeft pijn je de **afgelopen 4 weken** belemmerd bij je normale werkzaamheden (naar school gaan, huiswerk maken, baan)? | - helemaal niet - een klein beetje - nogal - veel - heel erg veel |

| 1. Kun je in onderstaande tabel aangeven hoeveel uur je ongeveer, in **de afgelopen zomer en winter**,   per week aan de volgende activiteiten hebt besteed?   - *Het gaat hier om activiteiten buiten je eventuele (betaalde) werk of school.* - *Vul ‘0’ in, Als een soort activiteit niet van toepassing is.* - *Rond ½-uren af naar boven (dus 1½ uur wordt 2 uur).*  | **soort activiteit in het afgelopen jaar** | **aantal uren per week** | | | --- | --- | --- | | **in de zomer** | **in de winter** | | 1. wandelen (incl. naar school of werk, boodschappen en vrije tijd) | **…** uur | **…** uur | | 1. fietsen (incl. naar school of werk, boodschappen en vrije tijd) | **…** uur | **…** uur | | 1. tuinieren / actief zijn in de natuur / buiten spelen | **…** uur | **…** uur | | 1. klussen/doe-het-zelven | **…** uur | **…** uur | | 1. sport en andere lichaamsbeweging (bv. zwemmen, joggen, tennissen, dans, gymles op school) | **…** uur | **…** uur | | 1. huishoudelijk werk (bv. je kamer opruimen, stofzuigen, bed opmaken) | **…** uur | **…** uur | | |
| --- | --- | --- | --- | --- | --- | --- | --- | --- | --- | --- | --- | --- | --- | --- | --- | --- | --- | --- | --- | --- | --- | --- | --- | --- |
| 1. In welke groep deel je, je activiteiten in het **afgelopen jaar**   in wat betreft lichaamsbeweging? | - hoofdzakelijk zittend (bv. bureauwerk of op school) - staand, soms lopend (bv. winkel, horeca, kappersbedrijf) - lopend met lichamelijke belasting (bv. verpleging) - zwaar lichamelijk werk (bv. schoonmaakwerk, werk op boerderij of tuinderij) - niet van toepassing (bv. arbeidsongeschikt) |

| 1. Wil je in onderstaand schema invullen welke sport(en) je **in de loop van je leven** hebt beoefend, of het in wedstrijdverband was,   hoeveel uur per week je aan die sport besteedde, en op welke leeftijd je dit deed?   - - - *Het gaat hier om activiteiten buiten je eventuele (betaalde) werk.*     - *Rond ½-uren af naar boven (dus 1 ½ uur wordt 2 uur).*     - *Bij een verandering in wedstrijdniveau of het aantal uren per week dat je een bepaalde sport beoefende dien je een nieuwe regel te gebruiken.*     - *Als je een sport momenteel nog beoefent, vul dan je huidige leeftijd in als eindleeftijd.*  |  |  | **wedstrijd** | | **hoeveel uur** | **leeftijd** | | | --- | --- | --- | --- | --- | --- | --- | |  | **sport** | **nee** | **ja** | **per week** | **van** | **tot** | | 1 |  | 🞎 | 🞎 | … uur | … jr | … jr | | 2 |  | 🞎 | 🞎 | … uur | … jr | … jr | | 3 |  | 🞎 | 🞎 | … uur | … jr | … jr | | 4 |  | 🞎 | 🞎 | … uur | … jr | … jr | | 5 |  | 🞎 | 🞎 | … uur | … jr | … jr | | 6 |  | 🞎 | 🞎 | … uur | … jr | … jr | | 7 |  | 🞎 | 🞎 | … uur | … jr | … jr | | 8 |  | 🞎 | 🞎 | … uur | … jr | … jr | |
| --- | --- | --- | --- | --- | --- | --- | --- | --- | --- | --- | --- | --- | --- | --- | --- | --- | --- | --- | --- | --- | --- | --- | --- | --- | --- | --- | --- | --- | --- | --- | --- | --- | --- | --- | --- | --- | --- | --- | --- | --- | --- | --- | --- | --- | --- | --- | --- | --- | --- | --- | --- | --- | --- | --- | --- | --- | --- | --- | --- | --- | --- | --- | --- | --- | --- | --- | --- | --- | --- | --- |

| **VERMOEIDHEID** | |
| --- | --- |
| **De vragen over dit onderwerp worden op 2 verschillende manieren gesteld. Lees alsjeblieft eerst de toelichting voordat je de vragen invult.**  Hieronder staan 4 uitspraken, waarmee je kunt aangeven hoe je je de **afgelopen 2 weken** hebt gevoeld. Je kunt elke vraag beantwoorden door in één van de zeven hokjes een kruisje te zetten. De plaats van het kruisje geeft aan in welke mate, dus hoe erg je vindt dat de uitspraak op je van toepassing is.  *Bijvoorbeeld, wanneer je vindt dat de uitspraak helemaal klopt, zet dan een kruisje in het linker hokje zo:*  Ja, dat klopt ☒□□□□□□ nee, dat klopt niet  *Wanneer je vindt dat het antwoord niet “ja, dat klopt”, maar ook niet “nee, dat klopt niet” is, zet dan een kruisje in het hokje dat het meeste overeenkomt met je gevoel. Bijvoorbeeld zo:*  Ja, dat klopt □□□□☒□□ nee, dat klopt niet | |
| 1. Ik voel me moe | Ja, dat klopt □□□□□□□ nee, dat klopt niet |
| 1. Ik ben gauw moe | Ja, dat klopt □□□□□□□ nee, dat klopt niet |
| 1. Ik voel me fit | Ja, dat klopt □□□□□□□ nee, dat klopt niet |
| 1. Lichamelijk voel ik me uitgeput | Ja, dat klopt □□□□□□□ nee, dat klopt niet |
| Neem in je gedachten **een normale week in de afgelopen maanden**: | |
| 1. Heb je vermoeidheidsklachten? | - ja - soms - nee *🡺 ga door naar vraag H1* |
| 1. Zo ja, hoe lang bestaat de vermoeidheid al?   *Je kunt dit in weken, maanden of jaren aangeven.* | …... weken, **of:** …… maanden, **of:** ….. jaren |

| 1. Is er volgens jou een aanwijsbare oorzaak voor de vermoeidheid (bijv. verhuizing, verandering van opleiding / werk)? | - ja, nl. ......................................................................................................   …………………………………………………………………………..   - nee - weet niet |
| --- | --- |

| **MENSTRUATIE**  *De volgende vragen gaan over jouw ontwikkeling van kind naar volwassene. De menstruatie (ongesteld zijn), het gebruik van de pil en hormonen spelen hierbij een belangrijke rol. Ook deze vragen zijn belangrijk voor ons onderzoek omdat we graag willen weten hoe die ontwikkeling bij jou verloopt.* | |
| --- | --- |
| 1. Op welke leeftijd of in welk jaar werd je voo**r** het eerst ongesteld? | - leeftijd : … jaar *of* : in het jaar ….. 🡺 *ga door met vraag H3* - ik ben (nog) nooit ongesteld geweest 🡺 *ga door met vraag I0* - weet niet 🡺 *ga door met vraag H2* |
| 1. Kun je aangeven hoe oud je ongeveer was toen je voor het eerst ongesteld werd? | - jonger dan 8 jaar - 8, 9, 10 of 11 jaar - 12, 13 of 14 jaar - 15 jaar of ouder |
| 1. Kun je aangeven of de eerste ongesteldheid spontaan kwam of pas nadat je medicijnen of hormonen gebruikte? | - spontaan, kwam vanzelf op gang - na gebruik van medicjinen of hormonen - weet niet |
| 1. Ben je de **afgelopen 12 maanden** tenminste 1 maal ongesteld geweest | - ja, datum laatste menstruatie: …/…/…… 🡺 *ga door met vraag H6* - nee, datum laatste menstruatie: …/…/…… |
| 1. Wat is de reden dat je in de **afgelopen 12 maanden** niet ongesteld bent geweest? | - spontaan, bleef vanzelf weg - door een operatie aan de geslachtsorganen - na het stoppen van de pil/prikpil - door chemotherapie of radiotherapie - door het gebruik van medicijnen (geen chemotherapie) - zwangerschap, borstvoeding - anders, namelijk………………………………………………………. - weet niet |
| 1. Wat is de gemiddelde lengte van je natuurlijke menstruele cyclus?   *De lengte van de cyclus wordt gerekend vanaf de eerste dag van de menstruatie tot de eerste dag van de volgende menstruatie.* Als je momenteel niet (meer) menstrueert of als je de pil gebruikt, gaat de vraag over het laatste jaar dat je normaal menstrueerde. | - minder dan 21 dagen - 21-25 dagen - 26-30 dagen - 31-35 dagen - 36-42 dagen - meer dan 42 dagen - heel onregelmatig - weet niet |

| **ANTICONCEPTIEPIL (*de* pil) en HORMOONGEBRUIK** |  |
| --- | --- |
| 1. Gebruik je de anticonceptiepil? | 🞎 ja *🡺 ga door met vraag I2*  🞎 nee |
| 1. Heb je ooit de anticonceptiepil gebruikt? | 🞎 ja  🞎 nee *🡺 ga door met vraag I4* |
| 1. Op welke leeftijd of in welk jaar heb je *voor het eerst* de anticonceptiepil gebruikt? | - leeftijd ……. jaar of in het jaar ………….. - weet niet |
| 1. Op welke leeftijd of in welk jaar heb je *voor het laatst* de anticonceptiepil gebruikt? Als je momenteel de anticonceptiepil gebruikt kun je, je huidige leeftijd invullen. | - leeftijd ……. jaar of in het jaar ………….. - weet niet |
| 1. Heb je ooit om botontkalking te voorkomen medicijnen met hormonen gebruikt of gebruik je deze momenteel? (Het gaat hierbij niet om een anticonceptiemiddel zoals de pil), | - ja - nee *🡺 ga door met vraag J1* |
| 1. Op welke leeftijd of in welk jaar heb je deze medicijnen met hormonen *voor het eerst* gebruikt? | - leeftijd ……. jaar of in het jaar ………….. - weet niet |
| 1. Op welke leeftijd of in welk jaar heb je deze medicijnen met hormonen *voor het laatst* gebruikt? Als je deze medicijnen momenteel gebruikt kunt je, je huidige leeftijd invullen. | - leeftijd ……. jaar of in het jaar ………….. - weet niet |

| **SEKSUALITEIT** *Seksualiteit hoort ook bij de normale ontwikkeling van kind naar volwassene. En omdat we graag willen weten of de ontwikkeling bij jou normaal verloopt willen we je ook wat vragen over seks.* | |
| --- | --- |
| 1. Ben je seksueel actief?   *Onder seksueel actief wordt verstaan strelen onder kleren, voorspel, masturbatie, geslachtsgemeenschap* of een combinatie.*  ** geslachtsgemeenschap = echt met elkaar naar bed gaan* | - nee *🡺 ga door met vraag J8* - ja |
| 1. Hoe oud was je toen je voor het eerst seksueel actief werd? | - leeftijd: … jaar - weet niet |
| 1. Het ging toen om: | - strelen onder kleren - voorspel - masturbatie - geslachtsgemeenschap *🡺 ga door met vraag J6* |
| 1. Heb je ooit geslachtsgemeenschap gehad? | - nee *🡺 ga door met vraag J6* - ja |
| 1. Hoe oud was je toen? | - leeftijd: … jaar - weet niet |
| 1. Heb je problemen op seksueel gebied? | - nee *🡺 ga door met vraag J8* - ja |
| 1. Welke problemen heb je hierbij ontdekt ? | ………………………………………………………………………  ………………………………………………………………………  ……………………………………………………………………… |
| 1. Ik voel me aangetrokken tot | - jongens - meisjes - beiden - weet niet |

| **ROKEN, ALCOHOL, DRUGS** | |
| --- | --- |
| 1. Heb je ooit gerookt of rook je nu? | - ja - nee *🡺 ga door met vraag L7* |
| 1. Heb je ooit, **langer dan een jaar**, minstens één sigaret per week gerookt? | - ja - nee *🡺 ga door met vraag L4* |
| 1. Op welke leeftijd of in welk jaar bent je begonnen met roken?   *Je hoeft maar één mogelijkheid in te vullen.* | - leeftijd: … jaar of: in het jaar: ….. |
| 1. Rook je **momenteel gemiddeld** meer dan één sigaret per week? | - ja , …… sigaretten per week *🡺 ga door met vraag L7* - nee, maar ik heb wel ooit gerookt |
| 1. Op welke leeftijd of in welk jaar ben je definitief gestopt met roken?   *Je hoeft maar één mogelijkheid in te vullen.* | - leeftijd: … jaar; of: in het jaar: ….. |
| 1. Hoeveel sigaretten rookte je gemiddeld per dag of per week het laatste jaar voordat je stopte met roken? | - … sigaretten per dag **of:** - … sigaretten per week |
| 1. Heb je ooit alcoholische drank gedronken? | - ja - nee *🡺 ga door met vraag L12* |
| 1. Heb je ooit , **langer dan een jaar**, minstens één glas alcoholische drank per week gebruikt (gemiddeld over de week)? | - ja - nee |
| 1. Drink je **momenteel** meer dan één glas alcoholische drank per week (gemiddeld)?   Zo ja, sinds wanneer is dit? | - ja, sinds leeftijd: … jaar of: sinds het jaar: ….. - nee 🡺 *ga door met vraag L12* |
| 1. Hoeveel glazen alcoholische drank drink je **momenteel** **gemiddeld** per dag doordeweeks? | …….. glazen per dag doordeweeks |
| 1. Hoeveel glazen alcoholische drank drink je **momenteel** **gemiddeld** per dag in het weekend? | …….. glazen per dag in het weekend |
| 1. Heb je ooit drugs gebruikt (zoals hasj, wiet, cocaïne, heroïne, XTC of andere drugs)? | - ja - nee *🡺 ga door met vraag O1* |
| 1. In de onderstaande tabel kun je invullen welke drugs je ooit hebt gebruikt, zoals hasj, wiet, paddo’s, cocaïne, uppers, pep, speed, XTC, MDMA, GHB, LSD, NSIC, heroïne, crack of andere drugs. Zo ja, op welke leeftijd je de genoemde drugs voor het eerst gebruikt hebt. Het tweede deel van de tabel vraagt of je deze drugs **in het afgelopen jaar** meer dan 1x gebruikt hebt en zo ja, hoe vaak je deze drugs gemiddeld per maand of per jaar gebruikte in **het afgelopen jaar**.  | **drugs** | **hoe oud was je de eerste keer?** | **minstens 1x gebruikt in afgelopen jaar ?** | **zo ja, hoe vaak gebruikte je deze drugs**  **in het afgelopen jaar?** | | --- | --- | --- | --- | | ……………………………………. | .…. jaar | 🞎 nee 🞎 ja | ….. per maand OF … per jaar | | ……………………………………. | .…. jaar | 🞎 nee 🞎 ja | ….. per maand OF … per jaar | | ……………………………………. | .…. jaar | 🞎 nee 🞎 ja | ….. per maand OF … per jaar | | ……………………………………. | .…. jaar | 🞎 nee 🞎 ja | ….. per maand OF … per jaar | | ……………………………………. | .…. jaar | 🞎 nee 🞎 ja | ….. per maand OF … per jaar | | |

**TOT SLOT**

We zijn nu aan het einde van de vragenlijst gekomen, maar willen je nog 3 algemene vragen stellen en daarna is er ruimte om nog opmerkingen in te vullen. Het kan zijn dat je op bepaalde vragen terug wilt komen. Als dat zo is, vergeet dan niet te vermelden over welke vraag je een opmerking plaatst in het kader hieronder. Het kan ook zijn dat je opmerkingen aan ons als onderzoekers wilt geven, daar kun je deze ruimte ook voor gebruiken.

| 1. Heb je bovenstaande vragen zelf ingevuld? | - ja, ik heb de vragenlijst alleen ingevuld 🡺 *ga door naar vraag O3* - nee, ik heb de vragen samen met iemand anders ingevuld - nee, iemand anders heeft de vragenlijst ingevuld |
| --- | --- |
| 1. Zo nee, wie was dat? | - ouder(s) - broer/zus - een vriend of vriendin - verzorger/persoonlijk begeleider - anders namelijk ……………………………………… |
| 1. Als we naar aanleiding van deze vragenlijst nog vragen hebben, vind je het dan goed dat we contact met je opnemen? Zo ja, kun je dan alsjeblieft je telefoonnummer en/of je email adres invullen? | - nee, ik wil niet dat u nog contact met mij opneemt over deze vragenlijst - ja, u mag contact met mij opnemen over deze vragenlijst   telefoonnummer …………………………………..  email adres ……………………………………….. |

| **Ruimte voor aanvullende opmerkingen** |
| --- |

**
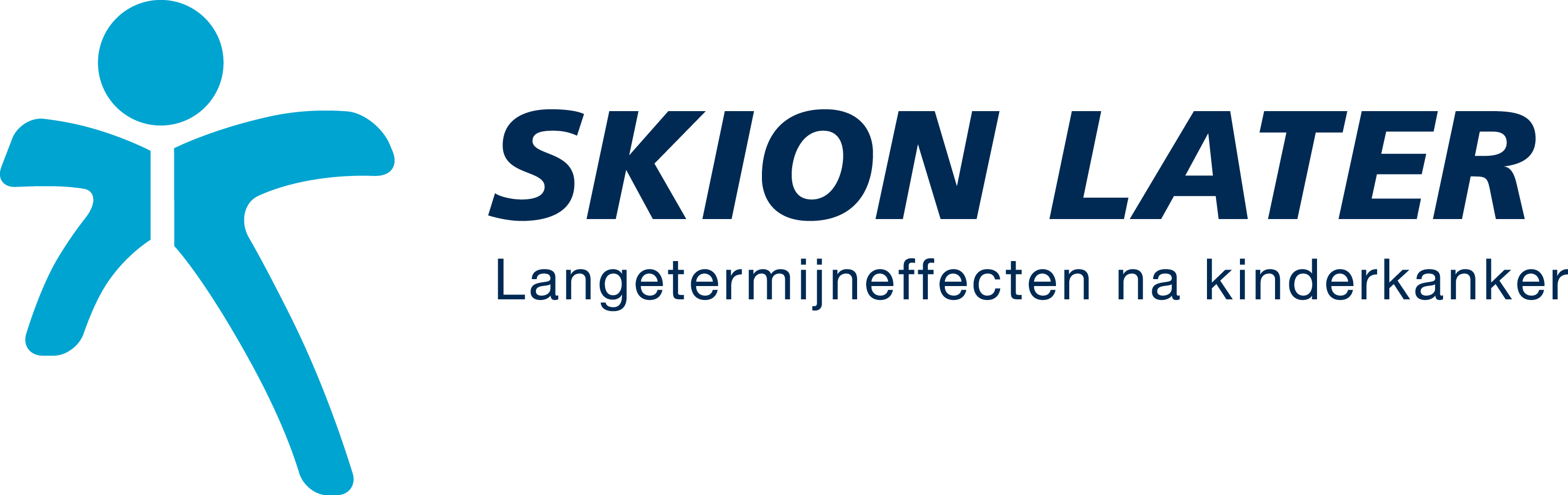
**

LATER VRAGENLIJST voor JONGENS 12 t/m 17 jr

Versie A2 JONGENS 12tm17 papier 2013 03 20

VRAGENLIJST

| LATER nummer  Datum invullen vragenlijst: | …………………………..……….…………..  …………………………..……….………….. |
| --- | --- |

**TOELICHTING BIJ HET INVULLEN VAN DE VRAGENLIJST**

- Het is de bedoeling dat deze vragenlijst ingevuld wordt door de persoon aan wie de vragenlijst is gestuurd; aan jou dus! Het gaat om je ***eigen*** antwoorden. Wanneer je de vragen zelf niet kunt lezen of invullen, kan iemand anders je helpen met het invullen van de vragen. Je moet wel zelf de antwoorden geven.
- Wij verzoeken je zoveel mogelijk alle vragen te beantwoorden. Soms kun je vragen overslaan, dit wordt dan duidelijk aangegeven bij de vraag. Kruis per vraag één antwoord aan. Als er meerdere antwoorden mogelijk zijn, dan wordt dat bij elke vraag apart aangegeven. Als je het antwoord op een vraag niet meer precies weet, bijvoorbeeld een leeftijd of een datum, probeer dan een zo goed mogelijke ***schatting*** te geven.
- Het kan voorkomen dat je in deze vragenlijst om (medische) informatie wordt gevraagd die jouw behandelend arts al heeft, maar de onderzoeker nog niet. Daarom hebben we deze vragen nogmaals opgenomen in de vragenlijst.
- Als je bij een vraag te weinig ruimte hebt kun je de rest van het antwoord vermelden bij het opmerkingen veld, op de laatste bladzijde. Schrijf dan alsjeblieft wel het nummer van de vraag op, waar het antwoord bij hoort.
- Probeer zoveel mogelijk binnen het hokje te blijven. Hieronder volgen een aantal voorbeelden van verschillende vragen.

Bij sommige vragen word je gevraagd het juiste hokje aan te kruisen:

Voorbeeld: Heeft een week 7 dagen? 🗷 ja

🞏 nee

Als je een antwoord wilt herstellen, kun je op de volgende manier voor het juiste antwoord een pijltje zetten:

Voorbeeld: Heeft een week 7 dagen? 🗷 ja

🗷 nee

Bij andere vragen is het de bedoeling dat je iets invult:

Voorbeeld: Hoeveel maanden heeft een jaar? ***12*** maanden

- Wij willen je nogmaals laten weten dat alle gegevens die je invult strikt vertrouwelijk behandeld worden. De gegevens worden opgeslagen met een code, dus zonder jouw naam en adres.

**Je kunt nu beginnen met de vragenlijst en we willen je nu vast bedanken voor het invullen !**

| **ALGEMEEN** | |
| --- | --- |
| 1. Op welke datum ben je geboren ? | ….… - …... - ….……. |
| 1. Wat is de geboorteplaats ? | geboorteplaats: ………………………………………………………… |
| 1. Ligt jouw geboorteplaats in Nederland? | - ja - nee |
| 1. Hoeveel broers en zussen heb je ?  *Halfzussen en halfbroers tellen niet mee.* | …... broers  …... zussen   - weet ik niet |
| 1. Zit je op school? | - ja - nee 🡺 *ga door met vraag A7* |
| 1. Zo ja, wat voor soort school? | - regulier basisonderwijs (lagere school) - speciaal basisonderwijs - voortgezet speciaal onderwijs - praktijkonderwijs - VMBO - middelbaar beroepsonderwijs (MBO) - HAVO - VWO, Gymnasium - hoger beroepsonderwijs (HBO) - anders, namelijk …………………………………..…………………. |
| 1. Wat wil je later worden? | …………………………………………………………………………… |

| **LICHAMELIJKE ACTIVITEITEN** |
| --- |
| **De volgende vragen gaan over dagelijkse bezigheden.**   1. Word je door je gezondheid de **afgelopen 4 weken** beperkt bij deze bezigheden. Zo ja, in welke mate?  |  | **Ja, heel erg beperkt** | **Ja, een beetje beperkt** | **Nee, helemaal niet beperkt** | | --- | --- | --- | --- | | 1. **Forse inspanning**   (zoals hardlopen, zware voorwerpen tillen, inspannend sporten) | 🞎 | 🞎 | 🞎 | | 1. **Matige inspanning**   (zoals het verplaatsen van een tafel, stofzuigen, fietsen) | 🞎 | 🞎 | 🞎 | | 1. Tillen of boodschappentas dragen | 🞎 | 🞎 | 🞎 | | 1. **Een paar** trappen oplopen | 🞎 | 🞎 | 🞎 | | 1. **Eén** trap oplopen | 🞎 | 🞎 | 🞎 | | 1. Buigen, knielen, of bukken | 🞎 | 🞎 | 🞎 | | 1. **Meer dan een kilometer** lopen | 🞎 | 🞎 | 🞎 | | 1. **Een halve kilometer** lopen | 🞎 | 🞎 | 🞎 | | 1. **Honderd meter** lopen | 🞎 | 🞎 | 🞎 | | 1. Jezelf wassen en aankleden | 🞎 | 🞎 | 🞎 | |

| 1. In hoeverre heeft je lichamelijke gezondheid of hebben emotionele problemen je de **afgelopen 4 weken** belemmerd in je normale sociale bezigheden met gezin, vrienden, buren of anderen? | - helemaal niet - een beetje - nogal - veel - heel erg veel |
| --- | --- |
| 1. **Hoe vaak** hebben je lichamelijke gezondheid of emotionele problemen gedurende de **afgelopen 4 weken** je sociale activiteiten (zoals bezoek aan vrienden of uitgaan) belemmerd? | - voortdurend - meestal - soms - zelden - nooit |
| 1. Hoeveel pijn had je de **afgelopen 4 weken**? | - geen - heel licht - licht - nogal - ernstig - heel ernstig |
| 1. In welke mate heeft pijn je de **afgelopen 4 weken** belemmerd bij je normale werkzaamheden (naar school gaan, huiswerk maken, baan)? | - helemaal niet - een klein beetje - nogal - veel - heel erg veel |

| 1. Kun je in onderstaande tabel aangeven hoeveel uur je ongeveer, in **de afgelopen zomer en winter**,   per week aan de volgende activiteiten hebt besteed?   - *Het gaat hier om activiteiten buiten je eventuele (betaalde) werk of school.* - *Vul ‘0’ in, Als een soort activiteit niet van toepassing is.* - *Rond ½-uren af naar boven (dus 1½ uur wordt 2 uur).*  | **soort activiteit in het afgelopen jaar** | **aantal uren per week** | | | --- | --- | --- | | **in de zomer** | **in de winter** | | 1. wandelen (incl. naar school of werk, boodschappen en vrije tijd) | **…** uur | **…** uur | | 1. fietsen (incl. naar school of werk, boodschappen en vrije tijd) | **…** uur | **…** uur | | 1. tuinieren / actief zijn in de natuur / buiten spelen | **…** uur | **…** uur | | 1. klussen/doe-het-zelven | **…** uur | **…** uur | | 1. sport en andere lichaamsbeweging (bv. zwemmen, joggen, tennissen, dans, gymles op school) | **…** uur | **…** uur | | 1. huishoudelijk werk (bv. je kamer opruimen, stofzuigen, bed opmaken) | **…** uur | **…** uur | | |
| --- | --- | --- | --- | --- | --- | --- | --- | --- | --- | --- | --- | --- | --- | --- | --- | --- | --- | --- | --- | --- | --- | --- | --- | --- |
| 1. In welke groep deel je, je activiteiten in het **afgelopen jaar**   in wat betreft lichaamsbeweging? | - hoofdzakelijk zittend (bv. bureauwerk of op school) - staand, soms lopend (bv. winkel, horeca, kappersbedrijf) - lopend met lichamelijke belasting (bv. verpleging) - zwaar lichamelijk werk (bv. schoonmaakwerk, werk op boerderij of tuinderij) - niet van toepassing (bv. arbeidsongeschikt) |

| 1. Wil je in onderstaand schema invullen welke sport(en) je **in de loop van je leven** hebt beoefend, of het in wedstrijdverband was,   hoeveel uur per week je aan die sport besteedde, en op welke leeftijd je dit deed?   - - - *Het gaat hier om activiteiten buiten je eventuele (betaalde) werk.*     - *Rond ½-uren af naar boven (dus 1 ½ uur wordt 2 uur).*     - *Bij een verandering in wedstrijdniveau of het aantal uren per week dat je een bepaalde sport beoefende dien je een nieuwe regel te gebruiken.*     - *Als je een sport momenteel nog beoefent, vul dan je huidige leeftijd in als eindleeftijd.*  |  |  | **wedstrijd** | | **hoeveel uur** | **leeftijd** | | | --- | --- | --- | --- | --- | --- | --- | |  | **sport** | **nee** | **ja** | **per week** | **van** | **tot** | | 1 |  | 🞎 | 🞎 | … uur | … jr | … jr | | 2 |  | 🞎 | 🞎 | … uur | … jr | … jr | | 3 |  | 🞎 | 🞎 | … uur | … jr | … jr | | 4 |  | 🞎 | 🞎 | … uur | … jr | … jr | | 5 |  | 🞎 | 🞎 | … uur | … jr | … jr | | 6 |  | 🞎 | 🞎 | … uur | … jr | … jr | | 7 |  | 🞎 | 🞎 | … uur | … jr | … jr | | 8 |  | 🞎 | 🞎 | … uur | … jr | … jr | |
| --- | --- | --- | --- | --- | --- | --- | --- | --- | --- | --- | --- | --- | --- | --- | --- | --- | --- | --- | --- | --- | --- | --- | --- | --- | --- | --- | --- | --- | --- | --- | --- | --- | --- | --- | --- | --- | --- | --- | --- | --- | --- | --- | --- | --- | --- | --- | --- | --- | --- | --- | --- | --- | --- | --- | --- | --- | --- | --- | --- | --- | --- | --- | --- | --- | --- | --- | --- | --- | --- | --- |

| **VERMOEIDHEID** | |
| --- | --- |
| **De vragen over dit onderwerp worden op 2 verschillende manieren gesteld. Lees alsjeblieft eerst de toelichting voordat je de vragen invult.**  Hieronder staan 4 uitspraken, waarmee je kunt aangeven hoe je je de **afgelopen 2 weken** hebt gevoeld. Je kunt elke vraag beantwoorden door in één van de zeven hokjes een kruisje te zetten. De plaats van het kruisje geeft aan in welke mate, dus hoe erg je vindt dat de uitspraak op je van toepassing is.  *Bijvoorbeeld, wanneer je vindt dat de uitspraak helemaal klopt, zet dan een kruisje in het linker hokje zo:*  Ja, dat klopt ☒□□□□□□ nee, dat klopt niet  *Wanneer je vindt dat het antwoord niet “ja, dat klopt”, maar ook niet “nee, dat klopt niet” is, zet dan een kruisje in het hokje dat het meeste overeenkomt met je gevoel. Bijvoorbeeld zo:*  Ja, dat klopt □□□□☒□□ nee, dat klopt niet | |
| 1. Ik voel me moe | Ja, dat klopt □□□□□□□ nee, dat klopt niet |
| 1. Ik ben gauw moe | Ja, dat klopt □□□□□□□ nee, dat klopt niet |
| 1. Ik voel me fit | Ja, dat klopt □□□□□□□ nee, dat klopt niet |
| 1. Lichamelijk voel ik me uitgeput | Ja, dat klopt □□□□□□□ nee, dat klopt niet |
| Neem in je gedachten **een normale week in de afgelopen maanden**: | |
| 1. Heb je vermoeidheidsklachten? | - ja - soms - nee *🡺 ga door naar vraag H1* |
| 1. Zo ja, hoe lang bestaat de vermoeidheid al?   *Je kunt dit in weken, maanden of jaren aangeven.* | …... weken, **of:** …… maanden, **of:** ….. jaren |

| 1. Is er volgens jou een aanwijsbare oorzaak voor de vermoeidheid (bijv. verhuizing, verandering van opleiding / werk)? | - ja, nl. ......................................................................................................   …………………………………………………………………………..   - nee - weet niet |
| --- | --- |

| **PUBERTEIT**  *De volgende vragen gaan over jouw ontwikkeling van kind naar volwassene. Ook deze vragen zijn belangrijk voor ons onderzoek omdat we graag willen weten hoe die ontwikkeling bij jou verloopt.* | |
| --- | --- |
| 1. Op welke leeftijd kwam je in de puberteit (kreeg je oksel- en schaambeharing)? | - leeftijd: ….. jaar 🡺 *ga door met vraag H3* - weet ik niet |
| 1. Kun je aangeven hoe oud je ongeveer was toen je in de puberteit kwam? | - - jonger dan 11 jaar   - 11, 12, 13, 14 of 15 jaar   - 16 jaar of ouder |
| 1. Heb je medicijnen gekregen om de puberteit op te wekken? | - - nee   - ja, namelijk het medicijn:…………………………….   - weet ik niet |
| **SEKSUALITEIT**  *Seksualiteit hoort ook bij de normale ontwikkeling van kind naar volwassene. En omdat we graag willen weten of de ontwikkeling bij jou normaal verloopt willen we je ook wat vragen over seks.* | |
| 1. Ben je seksueel actief?   *Onder seksueel actief wordt verstaan strelen onder kleren, voorspel, masturbatie, geslachtsgemeenschap* of een combinatie.*  ** geslachtsgemeenschap = echt met elkaar naar bed gaan* | - nee *🡺 ga door met vraag J8* - ja |
| 1. Hoe oud was je toen je voor het eerst seksueel actief werd? | - leeftijd ……. jaar - weet niet |
| 1. Het ging toen om: | - strelen onder kleren - voorspel - masturbatie - geslachtsgemeenschap *🡺 ga door met vraag J6* |
| 1. Heb je ooit geslachtsgemeenschap gehad? | - nee *🡺 ga door met vraag J6* - ja |
| 1. Hoe oud was je toen? | - leeftijd …….jaar - weet niet |
| 1. Ervaar je zelf problemen op seksueel gebied? | - nee *🡺 ga door met vraag J8* - ja |
| 1. Welke problemen ervaar je? | ………………………………………………………………………  ………………………………………………………………………  ……………………………………………………………………… |
| 1. Ik voel me aangetrokken tot | - meisjes - jongens - beiden - weet niet |

| **ROKEN, ALCOHOL, DRUGS** | |
| --- | --- |
| 1. Heb je ooit gerookt of rook je nu? | - ja - nee *🡺 ga door met vraag L7* |
| 1. Heb je ooit, **langer dan een jaar**, minstens één sigaret per week gerookt? | - ja - nee *🡺 ga door met vraag L4* |
| 1. Op welke leeftijd of in welk jaar ben je begonnen met roken?   *Je hoeft maar één mogelijkheid in te vullen.* | - leeftijd: … jaar of: in het jaar: ….. |
| 1. Rook je **momenteel gemiddeld** meer dan één sigaret per week? | - ja , …… sigaretten per week *🡺 ga door met vraag L7* - nee, maar ik heb wel ooit gerookt |
| 1. Op welke leeftijd of in welk jaar ben je definitief gestopt met roken?   *Je hoeft maar één mogelijkheid in te vullen.* | - leeftijd: … jaar; of: in het jaar: ….. |
| 1. Hoeveel sigaretten rookte je gemiddeld per dag of per week het laatste jaar voordat je stopte met roken? | - … sigaretten per dag **of:** - … sigaretten per week |
| 1. Heb je ooit alcoholische drank gedronken? | - ja - nee *🡺 ga door met vraag L12* |
| 1. Heb je ooit , **langer dan een jaar**, minstens één glas alcoholische drank per week gebruikt (gemiddeld over de week)? | - ja - nee |
| 1. Drink je **momenteel** meer dan één glas alcoholische drank per week (gemiddeld)?   Zo ja, sinds wanneer is dit? | - ja, sinds leeftijd: … jaar of: sinds het jaar: ….. - nee *🡺 ga door met vraag L12* |

| 1. Hoeveel glazen alcoholische drank drink je **momenteel** **gemiddeld** per dag doordeweeks? | …….. glazen per dag doordeweeks |
| --- | --- |
| 1. Hoeveel glazen alcoholische drank drink je **momenteel** **gemiddeld** per dag in het weekend? | …….. glazen per dag in het weekend |
| 1. Heb je ooit drugs gebruikt (zoals hasj, wiet, cocaïne, heroïne, XTC of andere drugs)? | - ja - nee *🡺 ga door met vraag O1* |
| 1. In de onderstaande tabel kun je invullen welke drugs je ooit hebt gebruikt, zoals hasj, wiet, paddo’s, cocaïne, uppers, pep, speed, XTC, MDMA, GHB, LSD, NSIC, heroïne, crack of andere drugs. Zo ja, op welke leeftijd heb je de genoemde drugs voor het eerst gebruikt? Het tweede deel van de tabel vraagt of je deze drugs **in het afgelopen jaar** meer dan 1x gebruikt hebt en zo ja, hoe vaak je deze drugs gemiddeld per maand of per jaar gebruikte in **het afgelopen jaar**. | |
| | **drugs** | **hoe oud was je de eerste keer?** | **minstens 1x gebruikt in afgelopen jaar ?** | **zo ja, hoe vaak gebruikte je deze drugs**  **in het afgelopen jaar?** |  | | --- | --- | --- | --- | --- | | ……………………………………. | .…. jaar | 🞎 nee 🞎 ja | ….. per maand OF … per jaar |  | | ……………………………………. | .…. jaar | 🞎 nee 🞎 ja | ….. per maand OF … per jaar |  | | ……………………………………. | .…. jaar | 🞎 nee 🞎 ja | ….. per maand OF … per jaar |  | | ……………………………………. | .…. jaar | 🞎 nee 🞎 ja | ….. per maand OF … per jaar |  | | ……………………………………. | .…. jaar | 🞎 nee 🞎 ja | ….. per maand OF … per jaar |  | | |
|  | |

**TOT SLOT**

We zijn nu aan het einde van de vragenlijst gekomen, maar willen je nog 3 algemene vragen stellen en daarna is er ruimte om nog opmerkingen in te vullen. Het kan zijn dat je op bepaalde vragen terug wilt komen. Als dat zo is, vergeet dan niet te vermelden over welke vraag je een opmerking plaatst in het kader hieronder. Het kan ook zijn dat je opmerkingen aan ons als onderzoekers wilt geven, daar kun je deze ruimte ook voor gebruiken.

| 1. Heb je bovenstaande vragen zelf ingevuld? | - ja, ik heb de vragenlijst alleen ingevuld *🡺 ga door naar vraag O3* - nee, ik heb de vragen samen met iemand anders ingevuld - nee, iemand anders heeft de vragenlijst ingevuld |
| --- | --- |
| 1. Zo nee, wie was dat? | - ouder (s) - broer/ zus - een vriend of vriendin - verzorger/ persoonlijk begeleider - anders namelijk ……………………………………… |
| 1. Als we naar aanleiding van deze vragenlijst nog vragen hebben, vind je het dan goed dat we contact met je opnemen? Zo ja, kun je dan s.v.p. je telefoonnummer en/of je email adres invullen? | - nee, ik wil niet dat u nog contact met mij opneemt over deze vragenlijst - ja, u mag contact met mij opnemen over deze vragenlijst   telefoonnummer …………………………………..  email adres ……………………………………….. |

| **Ruimte voor aanvullende opmerkingen** |
| --- |

**
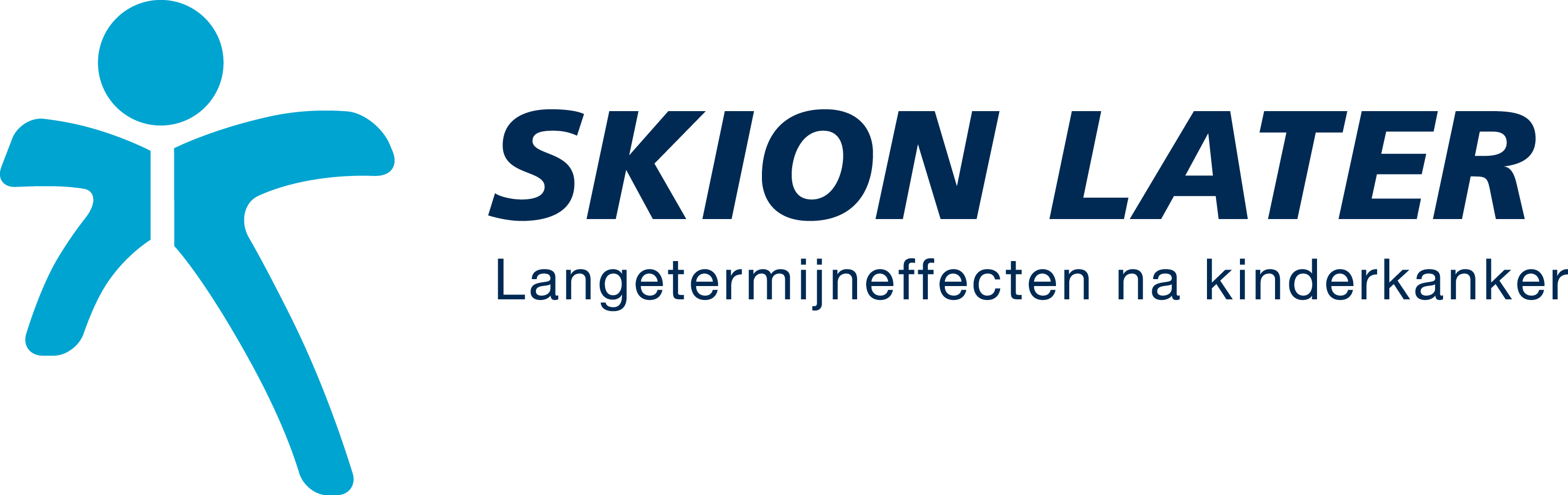
**

LATER VRAGENLIJST voor OUDERS/ VERZORGERS van kinderen van 12 t/m 17 jr

Versie A5 OUDERS 12tm17 papier 2013 03 20

VRAGENLIJST

| LATER nummer  Datum invullen vragenlijst: | …………………………..……….…………..  …………………………..……….………….. |
| --- | --- |

**TOELICHTING BIJ HET INVULLEN VAN DE VRAGENLIJST**

- Het is de bedoeling dat deze vragenlijst ingevuld wordt door de ouders of verzorgers van het kind dat op jonge leeftijd is behandeld voor kanker of een aanverwante aandoening. Wanneer u de vragen zelf niet kunt lezen of invullen, kan iemand anders u helpen met het invullen van de vragen.

U moet wel zelf de antwoorden geven.

- Wij verzoeken u zoveel mogelijk alle vragen te beantwoorden. Soms kunt u vragen overslaan, dit wordt dan ter plaatse duidelijk aangegeven. Als er meerdere antwoorden mogelijk zijn, dan wordt dat bij elke vraag afzonderlijk vermeld. Als u het antwoord op een vraag niet meer precies weet, bijvoorbeeld een leeftijd of een datum, probeert u dan een zo goed mogelijke ***schatting*** te geven.
- Enkele vragen hebben betrekking op uzelf en op uw kind toen uw kind nog een baby was. Informatie van een zwangerschapskaart of het groeiboekje van het consultatiebureau kunnen u wellicht helpen met het beantwoorden van de vragen.
- Het kan voorkomen dat u in deze vragenlijst om (medische) informatie wordt gevraagd die de behandelend arts van uw kind al heeft, maar de onderzoeker nog niet. Daarom hebben we deze vragen nogmaals opgenomen in de vragenlijst.
- Als u bij een vraag te weinig ruimte heeft kunt u de rest van het antwoord vermelden bij het opmerkingen veld, op de laatste bladzijde. Vermeldt u dan s.v.p. het nummer van de vraag waar het antwoord bij hoort.
- Wanneer er in de vragenlijst wordt gesproken over ‘uw kind’, dan bedoelen we de jongere die in het verleden is behandeld voor kanker of een aanverwante aandoening. De vragen over de vader en moeder hebben betrekking op de biologische vader en moeder
- Probeer zoveel mogelijk binnen het hokje te blijven. Hieronder volgen een aantal voorbeelden van verschillende vragen.

Bij sommige vragen wordt u gevraagd het juiste hokje aan te kruisen:

Voorbeeld: Heeft een week 7 dagen? 🗷 ja

🞏 nee

Als u een antwoord wilt herstellen, kunt u op de volgende manier voor het juiste antwoord een pijltje zetten:

Voorbeeld: Heeft een week 7 dagen? 🗷 ja

🗷 nee

Bij andere vragen is het de bedoeling dat u iets invult:

Voorbeeld: Hoeveel maanden heeft een jaar? ***12*** maanden

- Wij willen u er nogmaals op wijzen dat alle gegevens van u en uw kind strikt vertrouwelijk behandeld worden. Alle onderzoeksgegevens vallen onder de Nederlandse privacywetgeving. De gegevens worden opgeslagen met een code, dus zonder naam. De gegevens van u en uw kind zijn voor onderzoekers dus anoniem, dat wil zeggen, niet direct te herleiden tot uw persoon of die van uw kind.

**BIJ VOORBAAT DANK VOOR HET INVULLEN VAN DE VRAGENLIJST!**

| **ALGEMEEN** | |
| --- | --- |
| 1. Wat is de geboortedatum van uw kind? | ….… - …... - ….……. |
| 1. Na hoeveel weken en dagen zwangerschap is uw kind geboren?   *Kunt u aangeven of dit de precieze duur was of dat u het ongeveer geschat hebt* | …… weken en …… dagen   - precies - geschat |
| 1. Hoe zwaar was uw kind bij de geboorte?   *Kunt u aangeven of dit het precieze gewicht was of dat u het ongeveer geschat hebt* | …… gram   - precies - geschat |
| 1. Wat is de geboorteplaats van uw kind? | geboorteplaats: ………………………………………………………… |
| 1. Ligt de geboorteplaats van uw kind in Nederland? | - ja *🡺 ga door met vraag A7* - nee |
| 1. In welk land is uw kind geboren? | land: ……………………..………………………. |
| 1. Welke nationaliteit(en) heeft uw kind? | - - Nederlands   - anders, namelijk …………………………… |
| 1. Hoeveel broers en zussen heeft uw kind (gehad) met dezelfde   biologische ouders? | ….. broers  …... zussen   - - onbekend |

| 1. Is uw kind een deel van een tweeling/meerling? | - - nee   - ja, hij/zij heeft een tweelingbroer (gehad)   - ja, hij/zij heeft een tweelingzus (gehad)   - ja, hij/ zij is een deel van een drie-of vierling   - onbekend |
| --- | --- |
| 1. Wat is de huidige woonsituatie van uw kind? | - - - bij ouder(s)/ verzorgers wonend     - alleenwonend     - samenwonend met anderen, nl. ………………………………….     - niet-zelfstandig wonend, buiten het gezin ( bijvoorbeeld tehuis voor gehandicapten,   begeleid zelfstandig wonen)   - - - anders, namelijk: ……………………………………………………………………………   ……………………………………………………………………………. |
| 1. Welke van de volgende situaties is op uw kind van toepassing?   *Er zijn meerdere antwoorden mogelijk.* | - hij/zij heeft betaald werk, werkt ……uur per week als …………………….(beroep) sinds het jaar ..….. - hij/ zij volgt onderwijs/studeert sinds het jaar ..….. - hij/ zij is werkzoekende en/of ontvangt wachtgeld (RWW, WW, WWV) sinds het jaar ..….. - hij/ zij is arbeidsongeschikt, hij/zij ontvangt voor …. % een WAO of WIA uitkering sinds het jaar ..….. - hij/ zij ontvangt een Wajong uitkering, voor ….. % sinds het jaar ..….. - hij/ zij ontvangt een bijstandsuitkering sinds het jaar ..….. - hij/zij werkt niet en ontvangt geen uitkering sinds het jaar ..….. - anders, namelijk …………………………………..…………………………………   …………………………………………………………………… |
| 1. Volgt uw kind op dit moment een opleiding? | - ja - nee *🡺 ga door met vraag A15* |

| 1. Welke opleiding volgt uw kind op dit moment? | - regulier basisonderwijs (lagere school) - speciaal basisonderwijs - voortgezet speciaal onderwijs - praktijkonderwijs - VMBO - middelbaar beroepsonderwijs (MBO) - HAVO - VWO, Gymnasium - hoger beroeps onderwijs (HBO) - anders, namelijk …………………………………..…………………. |
| --- | --- |
| 1. Wat is de hoogst vorm van onderwijs die uw kind heeft afgerond? | - regulier basisonderwijs (lagere school) - speciaal basisonderwijs - voortgezet speciaal onderwijs - praktijkonderwijs - VMBO - middelbaar beroepsonderwijs (MBO) - HAVO - VWO, Gymnasium - hoger beroepsonderwijs (HBO) - anders, namelijk …………………………………..…………………. |
| 1. Wat is de hoogste vorm van onderwijs die de vader van uw kind heeft afgerond? | - regulier basisonderwijs (lagere school) - speciaal basisonderwijs - voortgezet speciaal onderwijs - praktijkonderwijs - lager beroepsonderwijs (LBO, LTS, LEAO, huishoudschool) - VMBO/ MAVO - middelbaar beroepsonderwijs (MBO, MLO, MEAO, MTS) - HAVO - VWO, Gymnasium - hoger beroeps onderwijs (HBO, HTS, HEAO) - universiteit - anders, namelijk …………………………………..………………… |
| 1. Wat is de hoogste vorm van onderwijs die de moeder van uw kind heeft afgerond? | - regulier basisonderwijs (lagere school) - speciaal basisonderwijs - voortgezet speciaal onderwijs - praktijkonderwijs - lager beroepsonderwijs (LBO, LTS, LEAO, huishoudschool) - VMBO/ MAVO - middelbaar beroepsonderwijs (MBO, MLO, MEAO, MTS) - HAVO - VWO, Gymnasium - hoger beroeps onderwijs (HBO, HTS, HEAO) - universiteit - anders, namelijk …………………………………..…………………. |
| 1. Wat is het beroep van de vader/ moeder van uw kind?   *Als de ouders niet meer werken of niet meer in leven zijn, kunt u het beroep invullen dat ze als laatste hebben gehad.*  *Als de ouders geen betaald werk hebben verricht*  *Kunt u “niet van toepassing” kiezen* | beroep vader:  …………………………………..………………….   - niet van toepassing   beroep moeder:  …………………………………..………………….   - niet van toepassing |
| 1. Hoe lang is uw kind op dit moment? | lengte …………….. cm |
| 1. Wat is het huidige gewicht van uw kind? | gewicht ……………. kg |
| 1. Hoe lang was de moeder van uw kind, als jonge vrouw (maximale lengte)? | lengte …………….. cm   - onbekend |
| 1. Hoe lang was de vader van uw kind, als jonge man   (maximale lengte)? | lengte …………….. cm   - onbekend |

| **MEDISCHE INFORMATIE** | | | | | | | | | | | | | | | |
| --- | --- | --- | --- | --- | --- | --- | --- | --- | --- | --- | --- | --- | --- | --- | --- |
| 1. Uw kind is op kinderleeftijd (0 t/m17 jr) behandeld voor kanker of een aanverwante aandoening. Heeft uw kind daarna nog een andere vorm van kanker, leukemie of een tumor gekregen of heeft uw kind deze op dit moment? (*deze vraag betreft een nieuwe, andere vorm van kanker; als dit een terugkeren van de eerdere kindertumor of leukemie was, mag u deze vraag met NEE beantwoorden en hoeft u het schema B2 niet in te vullen*)  - ja - nee *🡺 ga door met vraag B3* | | | | | | | | | | | | | | | |
| 1. Kunt u dan voor elke nieuwe tumor in het onderstaande schema aangeven in welk orgaan/deel van het lichaam deze zich bevond, welk soort tumor het was en in welk jaar of op welke leeftijd de diagnose gesteld werd? | | | | | | | | | | | | | | | |
|  | |  | **orgaan/lichaamsdeel** | | **soort tumor** | | | | **leeftijd bij diagnose** | | **OF** | | | **jaar van diagnose** | |
|  | | bij*voorbeeld* | *huid van de onderbuik, links* | | *melanoom* | | | | *9 jr ∕* | | | | | | |
|  | | 1 |  | |  | | | |  | | | | | | |
|  | | 2 |  | |  | | | |  | | | | | | |
|  | | 3 |  | |  | | | |  | | | | | | |
|  | | 4 |  | |  | | | |  | | | | | | |
| 1. Zou u in onderstaand schema willen invullen of uw kind nu of in het verleden één of meer van de onderstaande aandoeningen heeft gehad? Zo ja, kunt u dan s.v.p. ook schatten hoe oud uw kind was bij de diagnose of in welk jaar dat was en of uw kind hier **op dit moment** medicijnen voor gebruikt? *Op de stippellijnen kunt u toelichting geven over de precieze aandoening, Als u voor één of meerdere aandoeningen in schema B3 heeft aangegeven dat uw kind nu medicatie gebruikt, kunt u dan voor elk van deze aandoeningen bij B4 invullen hoe dat medicijn heet en op welke leeftijd of in welk jaar uw kind begonnen is met het gebruik?* | | | | | | | | | | | | | | | |
|  | | | | **gehad of nu aanwezig?** | | | | **leeftijd bij diagnose** | | **OF** | | **jaar van diagnose** | **medicijnen**  **op dit moment?** | | |
| 1. Hartinfarct | | | | 🞎 nee 🞎 ja | | | | ………… jr of …………… | | | | | 🞎 nee 🞎 ja | | |
| 1. Pijn op de borst (bij inspanning en/of rust) | | | | 🞎 nee 🞎 ja | | | | ………… jr of …………… | | | | | 🞎 nee 🞎 ja | | |
| 1. Hartklepafwijking | | | | 🞎 nee 🞎 ja | | | | ………… jr of …………… | | | | | 🞎 nee 🞎 ja | | |
| 1. Ontsteking van het hartzakje (pericarditis) | | | | 🞎 nee 🞎 ja | | | | ………… jr of …………… | | | | | 🞎 nee 🞎 ja | | |
|  | ***(1^e^ vervolg vraag B3 aandoeningen)*** | | | **gehad of nu aanwezig?** | | | | **leeftijd bij of: jaar**  **diagnose van diagnose** | | | | | **medicijnen**  **op dit moment?** | | |
|  | 1. Zwakke hartspier (cardiomyopathie) | | | 🞎 nee | | 🞎 ja | | ………… jr of …………… | | | | | 🞎 nee | | 🞎 ja |
|  | 1. Hartfalen | | | 🞎 nee | | 🞎 ja | | ………… jr of …………… | | | | | 🞎 nee | | 🞎 ja |
|  | 1. Hartritmestoornissen | | | 🞎 nee | | 🞎 ja | | ………… jr of …………… | | | | | 🞎 nee | | 🞎 ja |
|  | 1. Aangeboren hartafwijking nl……………………………………………... | | | 🞎 nee | | | 🞎 ja | ………… jr of …………… | | | | | 🞎 nee | | 🞎 ja |
|  | 1. Andere hartziekte, nl.: …………………………..………………………. | | | 🞎 nee | | 🞎 ja | | ………… jr of …………… | | | | | 🞎 nee | | 🞎 ja |
|  | 1. Beroerte (CVA / herseninfarct/hersenbloeding) | | | 🞎 nee | | 🞎 ja | | ………… jr of …………… | | | | | 🞎 nee | | 🞎 ja |
|  | 1. TIA (beroerte binnen 24 uur hersteld) | | | 🞎 nee | | 🞎 ja | | ………… jr of …………… | | | | | 🞎 nee | | 🞎 ja |
|  | 1. Vaatafwijkingen, nl.: ……………………………………………….……. | | | 🞎 nee | | 🞎 ja | | ………… jr of …………… | | | | | 🞎 nee | | 🞎 ja |
|  | 1. Een aandoening die een verhoogde stollingsneiging (trombose) veroorzaakt   Indien ja, welke ? 🞎 proteïne C deficiëntie  🞎 proteïne S deficiëntie  🞎 factor V Leiden mutatie  🞎 overig nl……………………………………. | | | 🞎 nee | | 🞎 ja | | ………… jr of ……………  ………… jr of ……………  ………… jr of ……………  ………… jr of …………… | | | | | 🞎 nee  🞎 nee  🞎 nee  🞎 nee | | 🞎 ja  🞎 ja  🞎 ja  🞎 ja |
|  | 1. Hoge bloeddruk (hypertensie) | | | 🞎 nee | | 🞎 ja | | ………… jr of …………… | | | | | 🞎 nee | | 🞎 ja |
|  | 1. Hoog cholesterol | | | 🞎 nee | | 🞎 ja | | ………… jr of …………… | | | | | 🞎 nee | | 🞎 ja |
|  | 1. Problemen met de maag of darmen | | | 🞎 nee | | 🞎 ja | | ………… jr of …………… | | | | | 🞎 nee | | 🞎 ja |
|  | ***(2^e^ vervolg vraag B3 aandoeningen)*** | | | **gehad of nu aanwezig?** | | | | **leeftijd bij of: jaar**  **diagnose van diagnose** | | | | | **medicijnen**  **op dit moment?** | | |
|  | 1. Longaandoeningen, nl.: ……………………………………………………… | | | 🞎 nee | | 🞎 ja | | ………… jr of ……………  ………… jr of …………… | | | | | 🞎 nee | | 🞎 ja |
|  | 1. Heeft uw kind in het **afgelopen jaar** een periode gehad waarin uw kind meer dan 6 weken aaneengesloten hoestte? | | | 🞎 nee | | 🞎 ja | | niet van toepassing (n.v.t) | | | | | 🞎 nee | | 🞎 ja |
|  | 1. Heeft uw kind meer dan 3x per jaar een infectie van de luchtwegen? | | | 🞎 nee | | 🞎 ja | | n.v.t | | | | | 🞎 nee | | 🞎 ja |
|  | 1. Heeft uw kind wel eens last (gehad) van urineweginfecties met koorts (nierbekkenontsteking)?   Indien ja, hoe vaak ? 🞎 1 keer  🞎 2-5 keer  🞎 meer dan 5 keer | | | 🞎 nee | | 🞎 ja | | n.v.t | | | | | n.v.t. | | |
|  | 1. Heeft uw kind andere problemen met de nieren (bijvoorbeeld slecht werkende nieren, nierstenen, teveel eiwit in de urine, cystes)?   Indien ja, welke …………………………..……………………………………  …………………………..…………………………………… | | | 🞎 nee | | 🞎 ja | | n.v.t    ………… jr of ……………  ………… jr of …………… | | | | | n.v.t.  🞎 nee 🞎 ja  🞎 nee 🞎 ja | | |
|  | 1. Problemen met de bijnieren, nl............................................................... | | | 🞎 nee | | 🞎 ja | | ………… jr of …………… | | | | | 🞎 nee | | 🞎 ja |
|  | 1. Leverproblemen, nl …………………………………………………………... | | | 🞎 nee | | 🞎 ja | | ………… jr of …………… | | | | | 🞎 nee | | 🞎 ja |
|  | 1. Problemen met het bewegingsapparaat (bijvoorbeeld arm/been/   elleboog/knie) nl : …………………………………………………………… | | | 🞎 nee | | 🞎 ja | | ………… jr of …………… | | | | | 🞎 nee | | 🞎 ja |
|  | 1. Suikerziekte (diabetes mellitus) | | | 🞎 nee | | 🞎 ja | | ………… jr of …………… | | | | | 🞎 nee | | 🞎 ja |
|  | ***(3^e^ vervolg vraag B3 aandoeningen)*** | | | **gehad of nu aanwezig?** | | | | **leeftijd bij of: jaar**  **diagnose van diagnose** | | | | | **medicijnen**  **op dit moment?** | | |
|  | 1. Epilepsieaanvallen | | | 🞎 nee | | 🞎 ja | | ………… jr of …………… | | | | | 🞎 nee | | 🞎 ja |
|  | 1. Is er bij uw kind door een oogarts staar geconstateerd? | | | 🞎 nee | | 🞎 ja | | ………… jr of …………… | | | | | n.v.t. | | |
|  | 1. Heeft uw kind een gehoorapparaat? | | | 🞎 nee | | 🞎 ja | | ………… jr of …………… | | | | | n.v.t. | | |
|  | 1. Heeft uw kind last van oorsuizen? | | | 🞎 nee | | 🞎 ja | | ………… jr of …………… | | | | | 🞎 nee | | 🞎 ja |
|  | 1. Is er bij uw kind sprake (geweest) van verminderde lengtegroei   (korte lichaamslengte)? | | | 🞎 nee | | 🞎 ja | | ………… jr of …………… | | | | | 🞎 nee | | 🞎 ja |
|  | 1. Verminderde schildklierfunctie (hypothyreoïdie) | | | 🞎 nee | | 🞎 ja | | ………… jr of …………… | | | | | 🞎 nee | | 🞎 ja |
|  | 1. Verhoogde schildklierfunctie (hyperthyreoïdie) | | | 🞎 nee | | 🞎 ja | | ………… jr of …………… | | | | | 🞎 nee | | 🞎 ja |
|  | 1. Schildklierknobbel (schildkliernodus) | | | 🞎 nee | | 🞎 ja | | ………… jr of …………… | | | | | 🞎 nee | | 🞎 ja |
|  | 1. Andere schildklieraandoening, nl. ………………………………………… | | | 🞎 nee | | 🞎 ja | | ………… jr of …………… | | | | | 🞎 nee | | 🞎 ja |
|  | 1. Andere probleem met hormonenregulatie, namelijk. ............................   ................................................................................................................... | | | 🞎 nee | | 🞎 ja | | ………… jr of …………… | | | | | 🞎 nee | | 🞎 ja |
| . | jj. Andere aandoening: ……………………………………………………….  ................................................................................................................. | | | 🞎 nee | | 🞎 ja | | ………… jr of …………… | | | | | 🞎 nee | | 🞎 ja |

| 1. Wilt u aangeven welke medicijnen uw kind op dit moment gebruikt voor de aandoeningen genoemd bij vraag B3 en op welke leeftijd of in welk jaar uw kind begonnen is met het gebruik? Als u niet weet hoe het medicijn heet, vult u dan s.v.p. de naam van de aandoening uit B3 in gevolgd door "weet niet". | | | | | | | | | | | | | | | | |
| --- | --- | --- | --- | --- | --- | --- | --- | --- | --- | --- | --- | --- | --- | --- | --- | --- |
|  | **naam medicijn** | | **aandoening** | | | | | | **begonnen op leeftijd** | | **of** | | **in het jaar** | | |  |
|  | bijvoorbeeld Thyroxine (Thyrax) | | Verminderde schildklierfunctie | | | | | |  | |  | | 2000 | | |  |
|  | 1 | |  | | | | | |  | |  | |  | | |  |
|  | 2 | |  | | | | | |  | |  | |  | | |  |
|  | 3 | |  | | | | | |  | |  | |  | | |  |
|  | 4 | |  | | | | | |  | |  | |  | | |  |
|  | 5 | |  | | | | | |  | |  | |  | | |  |
|  | 6 | |  | | | | | |  | |  | |  | | |  |
|  | 7 | |  | | | | | |  | |  | |  | | |  |
|  | 8 | |  | | | | | |  | |  | |  | | |  |
|  |  | |  | | | | | |  | |  | |  | | |  |
| 1. Gebruikt uw kind **op dit moment** naast de medicijnen die u eventueel hierboven heeft genoemd, nog andere medicijnen of injecties? Denk daarbij bijvoorbeeld aan pijnstillers die u vaker dan 1x per week gebruikt (bijv. aspirine, paracetamol, Ibuprofen) of hormonen. | | | | - ja - nee *🡺 ga door met vraag B7* | | | | | | | | | | | | |
| 1. Zo ja, welke medicijnen zijn dit en op welke leeftijd of in welk jaar is uw kind begonnen met het nemen van deze medicijnen? | | | | | | | | | | | | | | | | |
|  | | **naam medicijn** | | | **begonnen op leeftijd** | | **of** | | | **in het jaar** | | | | |  | |
|  | | 1 | | |  | |  | | |  | | | | |  | |
|  | | 2 | | |  | |  | | |  | | | | |  | |
|  | | ***(vervolg vraag B6 andere medicijnen)***  **naam medicijn** | | | **begonnen op leeftijd** | | **of** | | | **in het jaar** | | | | |  | |
|  | | 3 | | |  | |  | | |  | | | | |  | |
|  | | 4 | | |  | |  | | |  | | | | |  | |
|  | | 5 | | |  | |  | | |  | | | | |  | |
|  | | 6 | | |  | |  | | |  | | | | |  | |
|  | | 7 | | |  | |  | | |  | | | | |  | |
|  | | 8 | | |  | |  | | |  | | | | |  | |
|  | |  | | |  | | | | | | | | | | | |
| B7 Heeft uw kind ooit de volgende behandeling of operatie ondergaan? | | | | | **gehad?** | | | **leeftijd bij behandeling/ operatie** | | | | **of** | | **jaar van**  **behandeling/**  **operatie** | | |
| a. Heeft uw kind ooit een vervanging van een hartklep gehad? | | | | | 🞎 nee | 🞎 ja | | ………… jr | | | | of | | …………… | | |
| b. Heeft uw kind ooit een andere operatie aan zijn/haar hart gehad (inclusief dotteren)?  nl………………………………………………………………………………. | | | | | 🞎 nee | 🞎 ja | | ………… jr | | | | of | | …………… | | |
| c. Heeft uw kind ooit een pacemaker/ ICD gekregen? | | | | | 🞎 nee | 🞎 ja | | ………… jr | | | | of | | …………… | | |
| d. Heeft uw kind ooit een orgaantransplantatie ondergaan?    Zo ja, welk orgaan ………………………………………………………… | | | | | 🞎 nee | 🞎 ja | | ………… jr | | | | of | | …………… | | |
| e. Is er bij uw kind ooit een heel orgaan of een arm of been verwijderd?  Zo ja, welk orgaan/ ledemaat (arm/been) ..……………………………… | | | | | 🞎 nee | 🞎 ja | | ………… jr | | | | of | | …………… | | |
| 1. Gaan u en uw kind ermee akkoord dat wij bij de huisarts/specialist van uw kind, over de door u bij de vragen B2 en B3 gerapporteerde ziekten/aandoeningen eventueel aanvullende informatie opvragen?  - nee *🡺 ga door naar de vraag op de volgende bladzijde* - ja   **Zo ja**, zet dan hieronder samen met uw kind een handtekening:  Handtekening jongere ………………………………………………………….  Handtekening ouder/ verzorger 1:………………………………………………………  Handtekening ouder/ verzorger 2:………………………………………………………  **Zo ja**, wilt u dan zo vriendelijk zijn hieronder de adresgegevens van de huisarts te vermelden?  Naam huisarts …………………………………………………………………………………………….  Adres …………………………………………………………………………………………….    Postcode/Woonplaats …………………………………………………………………………………………….  Telefoon …………………………………………………………………………………………….  **Zo ja**, wilt u dan zo vriendelijk zijn hieronder per door u gerapporteerde ziekte/aandoening de adresgegevens van de betreffende specialist te vermelden?  *Als u meer ruimte nodig heeft kunt u verdere namen en adressen vermelden bij het opmerkingenveld op de laatste pagina van de vragenlijst*  1. Soort ziekte/aandoening: …………………………………………………………………………………………….  Naam specialist …………………………………………………………………………………………….  Ziekenhuis en afdeling …………………………………………………………………………………………….  2. Soort ziekte/aandoening: …………………………………………………………………………………………….  Naam specialist …………………………………………………………………………………………….  Ziekenhuis en afdeling ……………………………………………………………………………………………. | | | | | | | | | | | | | | | | |

| **ZIEKTES IN DE FAMILIE** |  |
| --- | --- |
| 1. Zijn er mensen in de familie van uw kind die kanker hebben gehad?   *Met familie bedoelen we biologische vader/ moeder/ grootvader/ grootmoeder/ broer(s)/ zus(sen)en uw kind(eren). Graag zowel het kalenderjaar als de leeftijd bij diagnose invullen. Als u het niet precies weet, wilt u dan een schatting geven, bijvoorbeeld, jonger dan 20 jaar, tussen 40 en 50 jaar, of bijvoorbeeld tussen 1970 en 1980.* | - ja *🡺 vul s.v.p. de onderstaande tabel in* - nee *🡺 ga door naar vraag D2* - weet niet *🡺 ga door naar vraag D2* |
| |  | **relatie tot familielid**  **(bijv. broer, moeder)** | **soort kanker** | **kalenderjaar van diagnose** | **leeftijd bij diagnose** | **als in leven,**  **hoe oud zijn ze nu** | **als overleden,**  **leeftijd bij overlijden** | | --- | --- | --- | --- | --- | --- | --- | | 1 |  |  |  |  |  |  | | 2 |  |  |  |  |  |  | | 3 |  |  |  |  |  |  | | 4 |  |  |  |  |  |  | | |
| 1. Zijn er mensen in de familie van uw kind die een hart- en vaatziekte hebben (gehad), bijvoorbeeld een hartinfarct, beroerte, hersenbloeding, hartfalen, familiaire hypercholesterolemie, hoge bloeddruk, familiaire ritmestoornis, stollingsziekte of suikerziekte/diabetes? | - ja *🡺 vul s.v.p. de onderstaande tabel in* - nee *🡺 ga door naar vraag E1* - weet niet *🡺 ga door naar vraag E1* |
| |  | **relatie tot familielid**  **(bijv. broer, moeder)** | **soort ziekte** | **kalenderjaar van diagnose** | **leeftijd bij diagnose** | **als in leven,**  **hoe oud zijn ze nu** | **als overleden,**  **leeftijd bij overlijden** | | --- | --- | --- | --- | --- | --- | --- | | 1 |  |  |  |  |  |  | | 2 |  |  |  |  |  |  | | 3 |  |  |  |  |  |  | | 4 |  |  |  |  |  |  | | |

| **BAAN en PSYCHOSOCIALE HULP** |  |
| --- | --- |
| 1. Heeft uw kind wel eens een (bij) baan gezocht? | - nee - ja |
| 1. Heeft uw kind wel eens problemen gehad bij het vinden van een   (bij) baan of opleiding in verband met de ziektegeschiedenis? | - nee *🡺 ga door met vraag F1* - ja |
| 1. Zo ja, in welk jaar was dat ongeveer en welke problemen betrof het? | jaar …………….  probleem: …………………………………………………………………………. |
| 1. Heeft uw kind wel eens problemen gehad bij het verkrijgen van een verzekering in verband met zijn/haar ziektegeschiedenis? | - nee *🡺 ga door met vraag G1* - ja |
| 1. Zo ja, in welk jaar was dat ongeveer en welke problemen betrof het? | jaar …………….  probleem: ………………………………………………………………………… |

| 1. Heeft uw kind ooit psychosociale hulp ontvangen of krijgt uw kind op dit moment psychosociale hulp? | | | - nee *🡺 ga door naar O2* - ja | | |
| --- | --- | --- | --- | --- | --- |
| 1. Zo ja, kunt u s.v.p. in het schema invullen wanneer uw kind hulp kreeg/krijgt, van wie uw kind hulp kreeg/krijgt, de reden voor de hulp en hoeveel maanden   de hulp geduurd heeft? | | | | | |
|  | **wanneer (jaren)** | **soort hulpverlener (maatschappelijk werker, psycholoog, psychiater)** | **reden** | **duur** |  |
|  | Van 2010 tot 2011 | Psycholoog | Leren omgaan met ziekteverleden | 8 maanden |  |
|  | 1. van ……… tot ………… |  |  | ………. maanden |  |
|  | 1. van ……… tot ………… |  |  | ………. maanden |  |
|  | 1. van ……… tot ………… |  |  | ………. maanden |  |
|  |  |  |  |  |  |

| **VRAGEN OVER U en/of de moeder van het kind over wie u de vragenlijst heeft ingevuld** |  |
| --- | --- |
| O2 Wat is uw relatie tot het kind over wie u de vragenlijst heeft ingevuld? | - vader - moeder - verzorger(s) - anders, namelijk…………………………………….. |
| 1. Indien u de moeder bent, wat is dan uw huidige leeftijd?   Indien u niet de moeder bent; leeft de moeder nog? | …………………………..jaar   - - ja, haar huidige leeftijd is ……….. jaar   - nee   - onbekend |
| 1. Is zij of bent u (moeder) in de overgang (menopauze) gekomen?   Zo ja, kunt u aangeven wanneer u / moeder in de overgang bent / is gekomen? | - - nee, de moeder is (nog) niet in de overgang gekomen 🡺 *Ga door met vraag P1*   - ja, ik ben / moeder is in de overgang gekomen. 🡺 *Ga door met vraag H3* - onbekend 🡺 *Ga door met vraag P1* - de baarmoeder en/of eierstokken van mij / moeder is / zijn verwijderd voordat ik / zij in de overgang kwam     🡺 *Ga door met vraag P1* |
| 1. Op welke leeftijd bent u / is moeder in de overgang gekomen? | - leeftijd : ………jaar - ik weet het niet precies, maar ongeveer:   - jonger dan 30 jaar   - 30 - 34 jaar   - 35 - 39 jaar   - 40 - 44 jaar   - 45 - 49 jaar   - 50 jaar of ouder   - ik weet de leeftijd helemaal niet |

| **BROERS/ ZUSSEN VOOR DE CONTROLEGROEP** | |
| --- | --- |
| 1. Heeft uw zoon/dochter één of meerdere (half)broers of (half)zussen? | - nee *🡺 ga door naar vraag O1* - ja, hij/zij heeft ….. broer(s)/ halfbroer(s) (svp aantal invullen) - ja, hij/zij heeft ….. zus(sen)/ halfzus(sen) (svp aantal invullen) |
| *Om het huidige vragenlijst-onderzoek goed te kunnen uitvoeren, is het belangrijk dat de gegevens van uw zoon/dochter vergeleken worden met gegevens van personen die in het verleden niet voor kinderkanker behandeld zijn. Broers en zussen zijn daar erg geschikt voor. U heeft aangegeven dat uw zoon/dochter broers en/of zussen heeft. Met de volgende vraag willen we u daarom vragen of we hen mogen benaderen om ook deze vragenlijst in te vullen.* ***Het invullen van deze vraag is niet verplicht****. Bovendien, als u ons toestemming geeft om (één van) de broers en/of zussen van uw zoon/dochter te benaderen, kunnen zij vervolgens zelf besluiten of zij WEL OF NIET willen deelnemen aan dit vragenlijst-onderzoek.* | |
| 1. Geeft u ons toestemming om de (half)broer(s) en/of (half)zus(sen) van uw zoon/dochter een uitnodiging te sturen voor deelname aan dit vragenlijst-onderzoek? | - ja - nee *🡺 ga naar door vraag O1* |
| De gegevens van de (half)broers of (half)zussen, die mogen worden benaderd, zijn:     1. Naam: …………………………………………………………..……..….….… Geslacht: m / v *     Adres: ……………………………………………………………..……..…………..………..…………    Postcode: …………………………. Woonplaats: ………............………………..….………………  Geboortedatum: …………………. Evt. emailadres/ telefoonnummer: ……..……………………     1. Naam: …………………………………………………………..……..….….… Geslacht: m / v *     Adres: ……………………………………………………………..……..…………..………..…………    Postcode: …………………………. Woonplaats: ………............………………..….………………  Geboortedatum: …………………. Evt. emailadres/ telefoonnummer: ……..……………………     1. Naam: …………………………………………………………..……..….….… Geslacht: m / v *     Adres: ……………………………………………………………..……..…………..………..…………    Postcode: …………………………. Woonplaats: ………............………………..….………………  Geboortedatum: …………………. Evt. emailadres/ telefoonnummer: ……..…………………… | |

**TOT SLOT**

| 1. Heeft u de vragen in deze vragenlijst zelf ingevuld?   *Dit geldt niet voor de vragen die gaan over uw familie of geboorte.* | - ja, ik heb de vragenlijst alleen ingevuld *🡺 ga door naar vraag O9* - nee, ik heb de vragen samen met iemand anders ingevuld - nee, iemand anders heeft de vragenlijst ingevuld |
| --- | --- |
| 1. Zo nee, (met) wie was dat? | - ouder (s) - broer/ zus - mijn partner/ echtgenoot - een vriend of vriendin - verzorger/ persoonlijk begeleider - anders namelijk ……………………………………… |
| O9  Indien we naar aanleiding van deze vragenlijst nog vragen hebben, vindt u het dan goed dat we contact met u opnemen? Zo ja, kunt u dan s.v.p. uw telefoonnummer en/of uw email adres invullen? | - nee, ik wil niet dat u nog contact met mij opneemt over deze vragenlijst - ja, u mag contact met mij opnemen over deze vragenlijst   telefoonnummer ……………..………………………………..  email adres …………………………………………………….. |

| **Ruimte voor aanvullende opmerkingen** |
| --- |

**
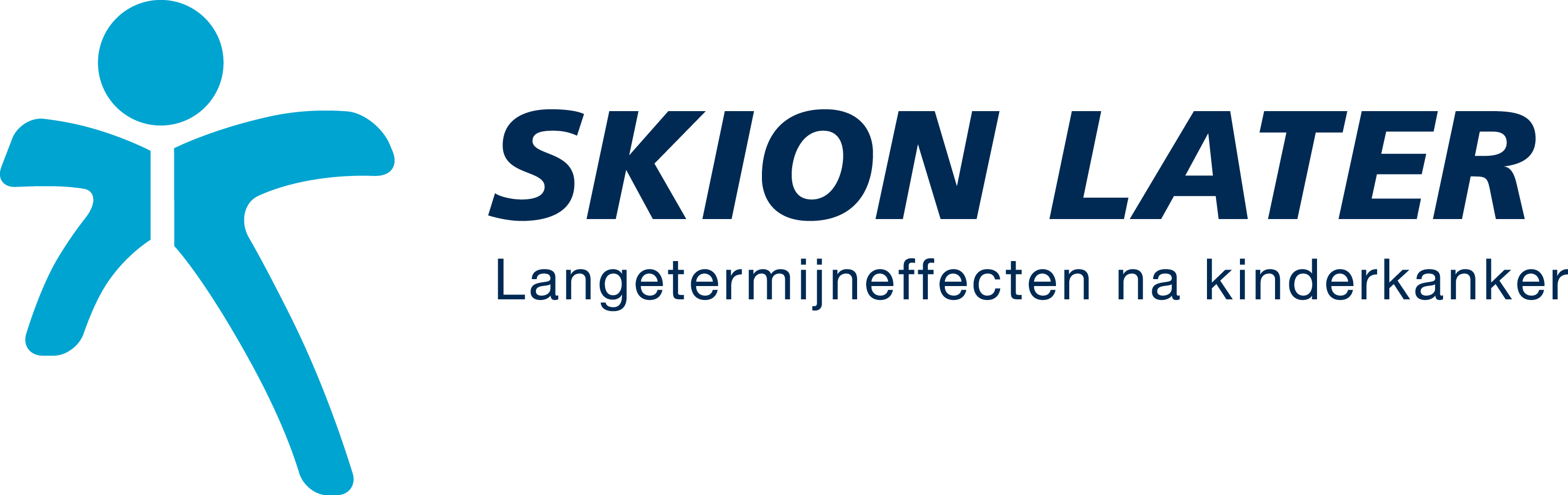
**

LATER VRAGENLIJST voor vrouwen

versie A3 VROUW - papier │ 20 maart 2013

VRAGENLIJST

| LATER nummer  Datum invullen vragenlijst: | …………………………..……….…………..  …………………………..……….………….. |
| --- | --- |

**TOELICHTING BIJ HET INVULLEN VAN DE VRAGENLIJST**

- Het is de bedoeling dat deze vragenlijst ingevuld wordt door de persoon aan wie de vragenlijst is gericht. Het gaat om uw ***eigen*** antwoorden. Wanneer u de vragen zelf niet kunt lezen of invullen, kan iemand anders u helpen met het invullen van de vragen. U moet wel zelf de antwoorden geven.
- Wij verzoeken u zoveel mogelijk alle vragen te beantwoorden. Soms kunt u vragen overslaan, dit wordt dan ter plaatse duidelijk aangegeven. Als er meerdere antwoorden mogelijk zijn, dan wordt dat bij elke vraag afzonderlijk vermeld. Als u het antwoord op een vraag niet meer precies weet, bijvoorbeeld een leeftijd of een datum, probeert u dan een zo goed mogelijke ***schatting*** te geven.
- Enkele vragen hebben betrekking op familieleden en op uzelf toen u nog een baby was. Indien uw ouders nog in leven zijn, kunnen zij u wellicht helpen om deze vragen te beantwoorden, bijvoorbeeld met informatie van een zwangerschapskaart of het groeiboekje van het consultatiebureau.
- Het kan voorkomen dat u in deze vragenlijst om (medische) informatie wordt gevraagd die uw behandelend arts al heeft, maar de onderzoeker nog niet. Daarom hebben we deze vragen nogmaals opgenomen in de vragenlijst.
- Als u bij een vraag te weinig ruimte heeft kunt u de rest van het antwoord vermelden bij het opmerkingen veld, op de laatste bladzijde. Vermeldt u dan s.v.p. het nummer van de vraag waar het antwoord bij hoort.

.

- Uw antwoorden worden strikt vertrouwelijk behandeld. Alle onderzoeksgegevens vallen onder de Nederlandse privacywetgeving. De gegevens worden opgeslagen met een code, dus zonder uw naam. Uw gegevens zijn voor onderzoekers dus anoniem, dat wil zeggen, niet direct te herleiden tot uw persoon.

- Probeert u zoveel mogelijk binnen het hokje te blijven. Hieronder volgen een aantal voorbeelden van verschillende vragen.

Bij sommige vragen wordt u gevraagd het juiste hokje aan te kruisen:

Voorbeeld: Heeft een week 7 dagen? 🗷 ja

🞏 nee

Als u een antwoord wilt herstellen, kunt u op de volgende manier voor het juiste antwoord een pijltje zetten:

Voorbeeld: Heeft een week 7 dagen? 🗷 ja

🗷 nee

Bij andere vragen is het de bedoeling dat u iets invult:

Voorbeeld: Hoeveel maanden heeft een jaar? ***12*** maanden

**BIJ VOORBAAT DANK VOOR HET INVULLEN VAN DE VRAGENLIJST!**

| **ALGEMEEN** | |
| --- | --- |
| 1. Wat is uw geboortedatum? | ….… - …... - ….……. |
| 1. Bij welke zwangerschapsduur bent u geboren?   *Kunt u aangeven of dit de precieze duur was of dat u het ongeveer geschat heeft* | …… weken en …… dagen   - precies - geschat |
| 1. Hoe zwaar was u bij de geboorte?   *Kunt u aangeven of dit het precieze gewicht was of dat u het ongeveer geschat heeft* | …… gram   - precies - geschat |
| 1. Wat is uw geboorteplaats? | geboorteplaats: ………………………………………………………… |
| 1. Ligt uw geboorteplaats in Nederland? | - ja *🡺 ga door met vraag A7* - nee |
| 1. In welk land bent u geboren? | land: ……………………..………………………. |
| 1. Welke nationaliteit(en) heeft u? | - - Nederlands   - anders, namelijk …………………………… |
| 1. Hoeveel broers en zussen heeft u (gehad) met dezelfde   biologische ouders als u? | ….. broers  …... zussen   - - onbekend |
| 1. Bent u deel van een tweeling/meerling? | - - nee   - ja, ik heb een tweelingbroer (gehad)   - ja, ik heb een tweelingzus (gehad)   - ja, ik ben deel van een drie-of vierling   - onbekend |
| 1. Wat is uw huidige woonsituatie? | - - bij ouder(s) wonend   - alleenwonend   - samenwonend met partner en/of kinderen   - samenwonend met anderen, nl. ………………………………….   - niet-zelfstandig wonend, buiten het gezin ( bijvoorbeeld tehuis voor gehandicapten, begeleid zelfstandig wonen)   - anders, namelijk: ……………………………………………………     …………………………………………………… |
| 1. Wat is uw huidige burgerlijke staat? | - - - ongehuwd, geen (vaste) relatie     - ongehuwd, wel (vaste) relatie     - gehuwd     - gescheiden     - weduwe     - anders, namelijk: …………………………………………………… |
| 1. Welke van de volgende situaties is op u van toepassing?   *Er zijn meerdere antwoorden mogelijk.* | - ik heb betaald werk, ik werk ……uur per week als …………………….(beroep) sinds het jaar ..….. - ik ben zelfstandig ondernemer, ik werk circa……uur per week als ……………………(beroep) sinds het jaar ..….. - ik volg onderwijs/studeer sinds het jaar ..….. - ik ben fulltime huisvrouw sinds het jaar ..….. - ik ben werkzoekende en/of ontvang wachtgeld (RWW, WW, WWV) sinds het jaar ..….. - ik ben arbeidsongeschikt, ik ben voor …. % afgekeurd en ontvang sinds het jaar ..….. een WAO of WIA uitkering - ik ontvang een Wajong uitkering, voor ….. % sinds het jaar ..….. - ik ontvang een bijstandsuitkering sinds het jaar ..….. - ik werk niet en ontvang geen uitkering sinds het jaar ..….. - anders, namelijk …………………………………..………………….   …………………………………..…………………. |

| 1. Volgt u op dit moment een opleiding? | - ja - nee *🡺 ga door met vraag A15* |
| --- | --- |
| 1. Welke opleiding volgt u op dit moment? | - voortgezet speciaal onderwijs - praktijkonderwijs - VMBO - middelbaar beroepsonderwijs (MBO, MLO, MEAO, MTS) - HAVO - VWO, Gymnasium - hoger beroeps onderwijs (HBO, HTS, HEAO) - universiteit - anders, namelijk …………………………………..…………………. |
| 1. Wat is de hoogst genoten opleiding die u heeft afgerond? | - regulier basisonderwijs (lagere school) - speciaal basisonderwijs - voortgezet speciaal onderwijs - praktijkonderwijs - lager beroepsonderwijs (LBO, LTS, LEAO, huishoudschool) - VMBO/ MAVO - middelbaar beroepsonderwijs (MBO, MLO, MEAO, MTS) - HAVO - VWO, Gymnasium - hoger beroeps onderwijs (HBO, HTS, HEAO) - universiteit - anders, namelijk …………………………………..…………………. |
| 1. Wat is de hoogste vorm van onderwijs die uw vader heeft afgerond? | - regulier basisonderwijs (lagere school) - speciaal basisonderwijs - voortgezet speciaal onderwijs - praktijkonderwijs - lager beroepsonderwijs (LBO, LTS, LEAO, huishoudschool) - VMBO/ MAVO - middelbaar beroepsonderwijs (MBO, MLO, MEAO, MTS) - HAVO - VWO, Gymnasium - hoger beroeps onderwijs (HBO, HTS, HEAO) - universiteit - anders, namelijk …………………………………..………………… |
| 1. Wat is de hoogste vorm van onderwijs die uw moeder heeft afgerond? | - regulier basisonderwijs (lagere school) - speciaal basisonderwijs - voortgezet speciaal onderwijs - praktijkonderwijs - lager beroepsonderwijs (LBO, LTS, LEAO, huishoudschool) - VMBO/ MAVO - middelbaar beroepsonderwijs (MBO, MLO, MEAO, MTS) - HAVO - VWO, Gymnasium - hoger beroeps onderwijs (HBO, HTS, HEAO) - universiteit - anders, namelijk …………………………………..…………………. |
| 1. Wat is het beroep van uw ouders?   *Indien uw ouders niet meer werken of niet meer in leven zijn, kunt u het beroep invullen dat ze als laatste hebben gehad. Indien uw ouders geen betaald werk hebben verricht kunt u “niet van toepassing” kiezen.* | beroep vader: …………………………………..………………….   - niet van toepassing   beroep moeder: …………………………………..………………….   - niet van toepassing |
| 1. Hoe lang bent u op dit moment? | lengte …………….. cm |
| 1. Wat is uw huidige gewicht? | gewicht ……………. kg |
| 1. Hoe lang was uw moeder als jonge vrouw (maximale lengte)? | lengte …………….. cm   - onbekend |
| 1. Hoe lang was uw vader als jonge man (maximale lengte)? | lengte …………….. cm   - onbekend |

| **MEDISCHE INFORMATIE** | | | | | | | | | | | | | | | | |
| --- | --- | --- | --- | --- | --- | --- | --- | --- | --- | --- | --- | --- | --- | --- | --- | --- |
| 1. U bent in uw jeugd behandeld voor kinderkanker of een aanverwante aandoening. Heeft u daarna nog een andere vorm van kanker, leukemie of een tumor gekregen of heeft u deze nu? (*deze vraag betreft een nieuwe, andere vorm van kanker; als dit een terugkeren van de eerdere kindertumor of leukemie was, mag u deze vraag met NEE beantwoorden en hoeft u het schema B2 niet in te vullen*)  - ja - nee *🡺 ga door met vraag B3* | | | | | | | | | | | | | | | | |
| 1. Kunt u dan voor elke nieuwe tumor in het onderstaande schema aangeven in welk orgaan/deel van het lichaam deze zich bevond, welk soort tumor het was en in welk jaar of op welke leeftijd de diagnose gesteld werd? | | | | | | | | | | | | | | | | |
|  |  | **orgaan/lichaamsdeel** | **soort tumor** | | | | **leeftijd bij diagnose** | | **OF** | | **jaar van diagnose** | | | |  | |
|  | bijvoorbeeld | Huid van de onderbuik links | melanoom | | | | 25 jaar | |  | | / | | | |  | |
|  | 1 |  |  | | | |  | |  | |  | | | |  | |
|  | 2 |  |  | | | |  | |  | |  | | | |  | |
|  | 3 |  |  | | | |  | |  | |  | | | |  | |
|  | 4 |  |  | | | |  | |  | |  | | | |  | |
| 1. Zou u in onderstaand schema willen invullen of u nu of in het verleden één of meer van de onderstaande aandoeningen heeft gehad? Zo ja, kunt u dan s.v.p. ook schatten hoe oud u was bij de diagnose of in welk jaar dat was en of u hier **op dit moment** medicijnen voor gebruikt? *Op de stippellijnen kunt u toelichting geven over de precieze aandoening, Indien u voor één of meerdere aandoeningen in schema B3 heeft aangegeven dat u nu medicatie gebruikt, kunt u dan voor elk van deze aandoeningen bij B4 invullen hoe dat medicijn heet en op welke leeftijd of in welk jaar u begonnen bent met het gebruik?* | | | | | | | | | | | | | | | | |
|  | | | | **gehad of nu aanwezig?** | | **leeftijd bij diagnose** | | **OF** | | **jaar van diagnose** | | **medicijnen**  **op dit moment?** | | | | |
| 1. Hartinfarct | | | | 🞎 nee | 🞎 ja | ………… jr | | of | | …………… | | 🞎 nee | | 🞎 ja | | |
| 1. Pijn op de borst (bij inspanning en/of rust) | | | | 🞎 nee | 🞎 ja | ………… jr | | of | | …………… | | 🞎 nee | | 🞎 ja | | |
| 1. Hartklepafwijking | | | | 🞎 nee | 🞎 ja | ………… jr | | of | | …………… | | 🞎 nee | | 🞎 ja | | |
| 1. Ontsteking van het hartzakje (pericarditis) | | | | 🞎 nee | 🞎 ja | ………… jr | | of | | …………… | | 🞎 nee | | 🞎 ja | | |
| ***(1^e^ vervolg vraag B3 aandoeningen)*** | | | | **gehad of nu aanwezig?** | | **leeftijd bij diagnose** | | **OF** | | **jaar van diagnose** | | **medicijnen op dit moment?** | | | | |
| 1. Zwakke hartspier (cardiomyopathie) | | | | 🞎 nee | 🞎 ja | ………… jr | | of | | …………… | | 🞎 nee | | 🞎 ja | | |
| 1. Hartfalen | | | | 🞎 nee | 🞎 ja | ………… jr | | of | | …………… | | 🞎 nee | | 🞎 ja | | |
| 1. Hartritmestoornissen | | | | 🞎 nee | 🞎 ja | ………… jr | | of | | …………… | | 🞎 nee | | 🞎 ja | | |
| 1. Aangeboren hartafwijking nl…………………………………………………… | | | | 🞎 nee | 🞎 ja | ………… jr | | of | | …………… | | 🞎 nee | | 🞎 ja | | |
| 1. Andere hartziekte, nl.: …………………………..……………………………… | | | | 🞎 nee | 🞎 ja | ………… jr | | of | | …………… | | 🞎 nee | | 🞎 ja | | |
| 1. Beroerte (CVA / herseninfarct/hersenbloeding) | | | | 🞎 nee | 🞎 ja | ………… jr | | of | | …………… | | 🞎 nee | | 🞎 ja | | |
| 1. TIA (beroerte binnen 24 uur hersteld) | | | | 🞎 nee | 🞎 ja | ………… jr | | of | | …………… | | 🞎 nee | | 🞎 ja | | |
| 1. Vaatafwijkingen, nl.: ……………………………………………….…………… | | | | 🞎 nee | 🞎 ja | ………… jr | | of | | …………… | | 🞎 nee | | 🞎 ja | | |
| 1. Een aandoening die een verhoogde stollingsneiging (trombose) veroorzaakt   Indien ja, welke? 🞎 proteïne C deficiëntie  🞎 proteïne S deficiëntie  🞎 factor V Leiden mutatie  🞎 overig nl……………………………………………. | | | | 🞎 nee | 🞎 ja | ………… jr  ………… jr  ………… jr  ………… jr | | of | | ……………  ……………  ……………  …………… | | 🞎 nee  🞎 nee  🞎 nee  🞎 nee | 🞎 ja  🞎 ja  🞎 ja  🞎 ja | | | |
| 1. Hoge bloeddruk (hypertensie) | | | | 🞎 nee | 🞎 ja | ………… jr | | of | | …………… | | 🞎 nee | 🞎 ja | | | |
| 1. Hoog cholesterol | | | | 🞎 nee | 🞎 ja | ………… jr | | of | | …………… | | 🞎 nee | 🞎 ja | | | |
| 1. Problemen met de maag of darmen | | | | 🞎 nee | 🞎 ja | ………… jr | | of | | …………… | | 🞎 nee | 🞎 ja | | | |
| ***(2^e^ vervolg vraag B3 aandoeningen)*** | | | | **gehad of nu aanwezig?** | | **leeftijd bij diagnose** | | **OF** | | **jaar van diagnose** | | **medicijnen op dit moment?** | | | | |
| 1. Longaandoeningen, nl.: ………………………………………………………... | | | | 🞎 nee | 🞎 ja | ………… jr | | of | | …………… | | 🞎 nee | | | | 🞎 ja |
| 1. Heeft u in het **afgelopen** jaar een periode gehad waarin u meer dan 6 weken aaneengesloten hoestte? | | | | 🞎 nee | 🞎 ja | niet van toepassing (n.v.t) | | | | | | 🞎 nee | | | | 🞎 ja |
| 1. Heeft u meer dan 3x per jaar een infectie van de luchtwegen? | | | | 🞎 nee | 🞎 ja | n.v.t. | | | | | | 🞎 nee | | | | 🞎 ja |
| 1. Heeft u wel eens last (gehad) van urineweginfecties met koorts (nierbekken- ontsteking)?   Indien ja, hoe vaak ? 🞎 1 keer  🞎 2-5 keer  🞎 meer dan 5 keer | | | | 🞎 nee | 🞎 ja | n.v.t. | | | | | | n.v.t. | | | | |
| 1. Heeft u andere problemen met uw nieren (bijvoorbeeld slecht werkende nieren, nierstenen, teveel eiwit in de urine, cystes)? | | | | 🞎 nee | 🞎 ja | n.v.t. | | | | | | n.v.t. | | | | |
| Indien ja, welke …………………………………………………………………    ………………………………………………………………… | | | |  |  | ………… jr  ………… jr | | of  of | | ……………  …………… | | 🞎 nee  🞎 nee | | | | 🞎 ja  🞎 ja |
| 1. Problemen met de bijnieren, nl ………………………………………………. | | | | 🞎 nee | 🞎 ja | ………… jr | | of | | …………… | | 🞎 nee | | | | 🞎 ja |
| 1. Leverproblemen, nl …………………………………………………………… | | | | 🞎 nee | 🞎 ja | ………… jr | | of | | …………… | | 🞎 nee | | | | 🞎 ja |
| 1. Problemen met het bewegingsapparaat (bijvoorbeeld arm/been/ elleboog/knie) nl : ………………….………................................................. | | | | 🞎 nee | 🞎 ja | ………… jr | | of | | …………… | | 🞎 nee | | | | 🞎 ja |
| 1. Suikerziekte (diabetes mellitus) | | | | 🞎 nee | 🞎 ja | ………… jr | | of | | …………… | | 🞎 nee | | | | 🞎 ja |
| ***(3^e^ vervolg vraag B3 aandoeningen)*** | | | | **gehad of nu aanwezig?** | | **leeftijd bij diagnose** | | **OF** | | **jaar van diagnose** | | **medicijnen**  **op dit moment?** | | | | |
| 1. Epilepsieaanvallen | | | | 🞎 nee | 🞎 ja | ………… jr | | of | | …………… | | 🞎 nee | | | | 🞎 ja |
| 1. Is er bij u door een oogarts staar geconstateerd? | | | | 🞎 nee | 🞎 ja | ………… jr | | of | | …………… | | n.v.t. | | | | |
| 1. Heeft u een gehoorapparaat? | | | | 🞎 nee | 🞎 ja | ………… jr | | of | | …………… | | n.v.t. | | | | |
| 1. Heeft u last van oorsuizen? | | | | 🞎 nee | 🞎 ja | ………… jr | | of | | …………… | | 🞎 nee | | | | 🞎 ja |
| 1. Is er bij u sprake (geweest) van verminderde lengtegroei (korte lichaamslengte)? | | | | 🞎 nee | 🞎 ja | ………… jr | | of | | …………… | | 🞎 nee | | | | 🞎 ja |
| 1. Verminderde schildklierfunctie (hypothyreoïdie) | | | | 🞎 nee | 🞎 ja | ………… jr | | of | | …………… | | 🞎 nee | | | | 🞎 ja |
| 1. Verhoogde schildklierfunctie (hyperthyreoïdie) | | | | 🞎 nee | 🞎 ja | ………… jr | | of | | …………… | | 🞎 nee | | | | 🞎 ja |
| 1. Schildklierknobbel (schildkliernodus) | | | | 🞎 nee | 🞎 ja | ………… jr | | of | | …………… | | 🞎 nee | | | | 🞎 ja |
| 1. Andere schildklieraandoening, nl ………………………………………........ | | | | 🞎 nee | 🞎 ja | ………… jr | | of | | …………… | | 🞎 nee | | | | 🞎 ja |
| 1. Ander probleem met hormonenregulatie, nl …………………………………   …………………………………………………………………………………… | | | | 🞎 nee | 🞎 ja | ………… jr | | of | | …………… | | 🞎 nee | | | | 🞎 ja |
| 1. Andere aandoening, nl ………………………………………………………..   …………………………………………………………………………………… | | | | 🞎 nee | 🞎 ja | ………… jr | | of | | …………… | | 🞎 nee | | | | 🞎 ja |

| 1. Wilt u aangeven welke medicijnen u op dit moment gebruikt voor de aandoeningen genoemd bij vraag B3 en op welke leeftijd of in welk jaar u begonnen bent met het gebruik? Als u niet weet hoe het medicijn heet, vult u dan s.v.p. de naam van de aandoening uit B3 in gevolgd door "weet niet". | | | | | | | | | | |
| --- | --- | --- | --- | --- | --- | --- | --- | --- | --- | --- |
|  | **naam medicijn** | **aandoening** | | | **begonnen op leeftijd** | | | **of** | **in het jaar** |  |
|  | bijvoorbeeld Thyroxine (Thyrax) | Verminderde schildklierfunctie | | |  | | |  | 1986 |  |
|  | 1 |  | | |  | | |  |  |  |
|  | 2 |  | | |  | | |  |  |  |
|  | 3 |  | | |  | | |  |  |  |
|  | 4 |  | | |  | | |  |  |  |
|  | 5 |  | | |  | | |  |  |  |
|  | 6 |  | | |  | | |  |  |  |
|  | 7 |  | | |  | | |  |  |  |
|  | 8 |  | | |  | | |  |  |  |
| 1. Gebruikt u **op dit moment** naast de medicijnen die u eventueel hierboven heeft genoemd, nog andere medicijnen of injecties? Denk daarbij bijvoorbeeld aan pijnstillers die u vaker dan 1x per week gebruikt (bijv. aspirine, paracetamol, Ibuprofen) of hormonen. | | | - ja - nee *🡺 ga door met vraag B7* | | | | | | | |
| 1. Zo ja, welke medicijnen zijn dit en op welke leeftijd of in welk jaar bent u begonnen met het nemen van deze medicijnen? | | | | | | | | | | |
|  | **naam medicijn** | | | **begonnen op leeftijd** | | **of** | **in het jaar** | | |  |
|  | 1 | | |  | |  |  | | |  |
|  | 2 | | |  | |  |  | | |  |

|  | ***(vervolg vraag B6 andere medicijnen)***  **naam medicijn** | **begonnen op leeftijd** | | | **of** | | **in het jaar** | |  |
| --- | --- | --- | --- | --- | --- | --- | --- | --- | --- |
|  | 3 |  | | |  | |  | |  |
|  | 4 |  | | |  | |  | |  |
|  | 5 |  | | |  | |  | |  |
|  | 6 |  | | |  | |  | |  |
|  | 7 |  | | |  | |  | |  |
|  | 8 |  | | |  | |  | |  |
| B7 Heeft u ooit de volgende behandeling of operatie ondergaan? | | **gehad?** | | **leeftijd bij behandeling/ operatie** | | **of:** | | **jaar van behandeling/ operatie** |  |
| a. Heeft u ooit een vervanging van een hartklep gehad? | | 🞎 nee | 🞎 ja | ………… jr | | of | | …………… |  |
| b. Heeft u ooit een andere operatie aan uw hart gehad (inclusief  dotteren)? nl………………………………………………………. | | 🞎 nee | 🞎 ja | ………… jr | | of | | …………… |  |
| c. Heeft u ooit een pacemaker/ ICD gekregen? | | 🞎 nee | 🞎 ja | ………… jr | | of | | …………… |  |
| d. Heeft u ooit een orgaantransplantatie ondergaan?    Zo ja, welk orgaan ………………………………………………… | | 🞎 nee | 🞎 ja | ………… jr | | of | | …………… |  |
| e. Is er bij u ooit een heel orgaan of een arm of been verwijderd?  Zo ja, welk orgaan/ ledemaat (arm/been) ……………………… | | 🞎 nee | 🞎 ja | ………… jr | | of | | …………… |  |
| 1. Gaat u ermee akkoord dat wij bij uw huisarts/specialist over de door u bij de vragen B2 en B3 gerapporteerde ziekten/aandoeningen eventueel aanvullende informatie opvragen?  - nee *🡺 ga door naar de vraag op de volgende bladzijde* - ja   **Zo ja**, wilt u dan hier uw handtekening zetten? ……………………………………………………………………………..  **Zo ja**, wilt u dan zo vriendelijk zijn hieronder de adresgegevens van uw huisarts te vermelden?  Naam huisarts …………………………………………………………………………………………….  Adres …………………………………………………………………………………………….    Postcode/Woonplaats …………………………………………………………………………………………….  Telefoon …………………………………………………………………………………………….  **Zo ja**, wilt u dan zo vriendelijk zijn hieronder per door u gerapporteerde ziekte/aandoening de adresgegevens van de betreffende specialist te vermelden?  *Indien u meer ruimte nodig heeft kunt u verdere namen en adressen vermelden bij het opmerkingenveld op de laatste pagina van de vragenlijst*  1. Soort ziekte/aandoening: …………………………………………………………………………………………….  Naam specialist …………………………………………………………………………………………….  Ziekenhuis en afdeling …………………………………………………………………………………………….  2. Soort ziekte/aandoening: …………………………………………………………………………………………….  Naam specialist …………………………………………………………………………………………….  Ziekenhuis en afdeling …………………………………………………………………………………………….  3. Soort ziekte/aandoening: …………………………………………………………………………………………….  Naam specialist …………………………………………………………………………………………….  Ziekenhuis en afdeling ……………………………………………………………………………………………. | | | | | | | | | |

| **ZIEKTES IN DE FAMILIE** | | | |  | | | | |
| --- | --- | --- | --- | --- | --- | --- | --- | --- |
| 1. Zijn er mensen in uw familie die kanker hebben gehad?     *Met familie bedoelen we uw biologische vader/ moeder/ grootvader/ grootmoeder/ broer(s)/ zus(sen) en uw kind(eren). Graag zowel het kalenderjaar als de leeftijd bij diagnose invullen. U mag ook een schatting geven.* | | | | - ja *🡺 vul s.v.p. de onderstaande tabel in* - nee *🡺 ga door naar vraag D2* - weet niet *🡺 ga door naar vraag D2* | | | | |
|  |  | **relatie tot familielid**  **(bijv. broer, moeder)** | **soort kanker** | **kalenderjaar van diagnose** | **leeftijd bij diagnose** | **indien in leven,**  **huidige leeftijd** | **indien overleden,**  **leeftijd bij overlijden** |  |
|  | 1 |  |  |  |  |  |  |  |
|  | 2 |  |  |  |  |  |  |  |
|  | 3 |  |  |  |  |  |  |  |
|  | 4 |  |  |  |  |  |  |  |
| 1. Zijn er mensen in de familie die een hart- en vaatziekte hebben (gehad), bijvoorbeeld een hartinfarct, beroerte, hersenbloeding, hartfalen, familiaire hypercholesterolemie, hoge bloeddruk, familiaire ritmestoornis, stollingsziekte of suikerziekte/diabetes? | | | | - ja *🡺 vul s.v.p. de onderstaande tabel in* - nee *🡺 ga door naar vraag E1* - weet niet *🡺 ga door naar vraag E1* | | | | |
|  |  | **relatie tot familielid**  **(bijv. broer, moeder)** | **soort ziekte** | **kalenderjaar van diagnose** | **leeftijd bij diagnose** | **indien in leven,**  **huidige leeftijd** | **indien overleden,**  **leeftijd bij overlijden** |  |
|  | 1 |  |  |  |  |  |  |  |
|  | 2 |  |  |  |  |  |  |  |
|  | 3 |  |  |  |  |  |  |  |
|  | 4 |  |  |  |  |  |  |  |
|  |  |  |  |  |  |  |  |  |

| **KEURING, BAAN en PSYCHOSOCIALE HULP** |  |
| --- | --- |
| 1. Heeft u wel eens problemen gehad bij een keuring in verband met   uw ziektegeschiedenis? | - nee *🡺 ga door met vraag E3* - ja - niet van toepassing *🡺 ga door met vraag E3* |
| 1. Zo ja, in welk jaar was dat ongeveer en op welke wijze? | jaar …………….  probleem: ………………………………………………………………………… |
| 1. Heeft u wel eens problemen gehad bij het vinden van een baan in verband met uw ziektegeschiedenis? | - nee *🡺 ga door met vraag F1* - ja |
| 1. Zo ja, in welk jaar was dat ongeveer en op welke wijze? | jaar …………….  probleem: …………………………………………………………………………. |
| 1. Heeft u wel eens problemen gehad bij het verkrijgen van een verzekering in verband met uw ziektegeschiedenis? | - nee *🡺 ga door met vraag G1* - ja |
| 1. Zo ja, in welk jaar was dat ongeveer en op welke wijze? | jaar …………….  probleem: ………………………………………………………………………… |

| 1. Heeft u ooit psychosociale hulp ontvangen of heeft u op dit moment hulp? | | | - nee *🡺 ga door met vraag H1* - ja | | |
| --- | --- | --- | --- | --- | --- |
| 1. Zo ja, kunt u s.v.p. in het schema invullen wanneer u hulp kreeg/krijgt, van wie u hulp kreeg, de reden voor de hulp en hoeveel maanden de hulp geduurd heeft? | | | | | |
|  | **wanneer (jaren)** | **soort hulpverlener (maatschappelijk werk, psycholoog, psychiater)** | **reden** | **duur** |  |
|  | Van 1995 tot 1996 | Psycholoog | Leren omgaan met ziekteverleden | 8 maanden |  |
|  | 1. van ……… tot ………… |  |  | ………. maanden |  |
|  | 1. van ……… tot ………… |  |  | ………. maanden |  |
|  | 1. van ……… tot ………… |  |  | ………. maanden |  |
|  |  |  |  |  | |

| **MENSTRUATIE EN ZWANGERSCHAP** |  |
| --- | --- |
| 1. Op welke leeftijd of in welk jaar werd u voor het eerst ongesteld? | - leeftijd : . . jaar of : in het jaar . . . . .. 🡺 ga door met vraag H3 - ik ben (nog) nooit ongesteld geweest 🡺 ga door met vraag H7 - weet niet 🡺 ga door met vraag H2 |
| 1. Kunt u aangeven hoe oud u ongeveer was toen u voor het eerst ongesteld werd? | - jonger dan 8 jaar - 8, 9, 10 of 11 jaar - 12, 13 of 14 jaar - 15 jaar of ouder |
| 1. Kunt u aangeven of de eerste ongesteldheid spontaan kwam of pas nadat u medicijnen of hormonen gebruikte? | - spontaan, kwam vanzelf op gang - na gebruik van medicjinen of hormonen - weet niet |
| 1. Bent u de **afgelopen** 12 maanden tenminste 1 maal ongesteld geweest? | - ja, datum laatste menstruatie: . . / . . / . . . . 🡺 g*a door met vraag H6* - nee, datum laatste menstruatie: . . / . . / . . . . |
| 1. Wat is de reden dat u in de **afgelopen 12 maanden** niet ongesteld bent geweest? | - - zwangerschap   - spontaan, bleef vanzelf weg   - door een operatie aan de geslachtsorganen   - na het stoppen van de pil/prikpil   - na borstvoeding   - door chemotherapie of radiotherapie   - door het gebruik van medicijnen (geen chemotherapie)   - anders, namelijk…………………………………………………………………..   - weet niet |
| 1. Wat is de gemiddelde lengte van uw natuurlijke menstruele cyclus? Als u momenteel niet (meer) menstrueert of als u de pil gebruikt, gaat de vraag over het laatste jaar dat u normaal menstrueerde.   *De lengte van de cyclus wordt gerekend vanaf de eerste dag van de menstruatie tot de eerste dag van de volgende menstruatie.* | - minder dan 21 dagen - 21-25 dagen - 26-30 dagen - 31-35 dagen - 36-42 dagen - meer dan 42 dagen - heel onregelmatig - weet niet |

| 1. Leeft uw moeder nog? | - ja, haar huidige leeftijd is ……….. jaar - nee - onbekend |
| --- | --- |
| 1. Is uw moeder in de overgang (menopauze) gekomen?   Zo ja, kunt u aangeven wanneer uw moeder in de overgang is gekomen? | - nee, mijn moeder is (nog)niet in de overgang gekomen🡺 Ga door met vraag H10 - ja, mijn moeder is in de overgang gekomen. 🡺 Ga door met vraag H9 - onbekend 🡺 *Ga door met vraag H10* - de baarmoeder en/of eierstokken van mijn moeder is/zijn verwijderd voordat zij in de overgang kwam     🡺 *Ga door met vraag H10* |
| 1. Op welke leeftijd is uw moeder in de overgang gekomen? | - leeftijd: ………jaar - ik weet het niet precies, maar ongeveer:   - jonger dan 30 jaar   - 30 - 34 jaar   - 35 - 39 jaar   - 40 - 44 jaar   - 45 - 49 jaar   - 50 jaar of ouder - ik weet de leeftijd helemaal niet |
| 1. Bent u ooit zwanger geweest? | - ja, aantal keren: ……………… - nee 🡺 *ga door met vraag H14* |
| 1. Zijn er levende kinderen geboren uit deze zwangerschap(pen)? | - ja, aantal: ……………… - nee 🡺 *ga door met vraag H13* |
| 1. Hoe oud was u bij de geboorte van uw eerste levend geboren kind of in welk jaar was dit?   *U hoeft maar één mogelijkheid in te vullen.* | leeftijd : . . jaar *of* : in het jaar . . . . .. |
| 1. Heeft u ooit een miskraam gehad? | - nee - ja |

| 1. Heeft u ooit een vruchtbaarheidskliniek of gynaecoloog /fertiliteitstarts bezocht omdat het niet lukte om zwanger te worden? | - nee 🡺 ga door met vraag H17 - ja |
| --- | --- |
| 1. Heeft u ooit medicijnen gebruikt die de eisprong opwekken? | - nee - ja, nl.: a. medicijn: ………………………….   b. aantal cycli: ………………………… |
| 1. Heeft u ooit een IVF / ICSI (= reageerbuisbevruchting) behandeling ondergaan? | - nee - ja, aantal keren: . . |
| 1. Heeft u op dit moment de wens om zwanger te worden? | 🞎 nee  🞎 ja |
| **ANTICONCEPTIE en HORMOONGEBRUIK** |  |
| 1. Heeft u ooit de anticonceptiepil gebruikt of gebruikt u deze momenteel? | 🞎 ja  🞎 nee *🡺 ga door met vraag I4* |
| 1. Op welke leeftijd of in welk jaar heeft u *voor het eerst* de anticonceptiepil gebruikt? | - leeftijd ……. jaar of in het jaar ………….. - weet niet |
| 1. Op welke leeftijd of in welk jaar heeft u *voor het laatst* de anticonceptiepil gebruikt? Als u momenteel de anticonceptiepil gebruikt kunt u uw huidige leeftijd invullen | - leeftijd ……. jaar of in het jaar ………….. - weet niet |
| 1. Heeft u ooit om overgangsklachten of botontkalking te voorkomen medicijnen met hormonen gebruikt of gebruikt u deze momenteel? (Het gaat hierbij niet om een anticonceptiemiddel zoals de pil.) | - ja - nee *🡺 ga door met vraag J1* |

| 1. Op welke leeftijd of in welk jaar heeft u deze medicijnen met hormonen *voor het eerst* gebruikt? | - leeftijd ……. jaar of in het jaar ………….. - weet niet |
| --- | --- |
| 1. Op welke leeftijd of in welk jaar heeft u deze medicijnen met hormonen *voor het laatst* gebruikt? Als u deze medicijnen momenteel gebruikt kunt u uw huidige leeftijd invullen | - leeftijd ……. jaar of in het jaar ………….. - weet niet |
| **SEKSUALITEIT** |  |
| 1. Ik voel me aangetrokken tot | - mannen - vrouwen - beiden |
| 1. Ervaart u problemen op seksueel gebied? | - nee *🡺 ga door met vraag J4* - ja |
| 1. Welke problemen ervaart u?   *Er zijn meerdere antwoorden mogelijk* | - seksuele behoefte/verlangen is verminderd (licht verminderd/erg verminderd/ afwezig - problemen met opgewonden raken tijdens seksuele activiteit - problemen met “vochtig” worden tijdens seksuele activiteit - problemen met klaarkomen - ongemakkelijk gevoel of pijn tijdens het binnendringen van de penis - ongemakkelijk gevoel of pijn na diepe penetratie van de penis (zgn. doorstootpijn) - anders, namelijk ……………………………………………………………………..   …………………………………………………………………….. |
| 1. Hoe oud was u toen u voor het eerst seksueel actief werd?   *Onder seksueel actief wordt verstaan strelen onder kleren, voorspel, masturbatie, geslachtsgemeenschap of een combinatie* | - leeftijd ……. jaar - weet niet |

| 1. Het ging toen om:   *Er zijn meerdere antwoorden mogelijk.* | | - strelen onder kleren - voorspel - masturbatie - geslachtsgemeenschap *🡺 ga door met vraag J7* |
| --- | --- | --- |
| 1. Heeft u ooit geslachtsgemeenschap gehad? | | - nee *🡺 ga door met vraag K1* - ja |
| 1. Hoe oud was u toen u voor het eerst geslachtsgemeenschap had? | | - leeftijd …….jaar - weet niet |
| **MAMMOGRAFIEËN** | |  |
| 1. Is bij u ooit een mammogram gemaakt, d.w.z. een speciale röntgenfoto van de borsten? | | - nee *🡺 ga door met vraag L2* - ja |
| 1. Hoeveel mammogrammen heeft u ooit gehad? | | - 1 - 2-5 - 6-10 - meer dan 10 - weet niet |
| 1. Laat u regelmatig een mammogram maken, d.w.z., eens per 2 jaar of elk jaar? | | - nee *🡺 ga door met vraag L2* - ja |
| 1. Vanaf welke leeftijd heeft u regelmatig een mammogram gehad? | | ……………. Jaar |
| 1. Wat is de reden dat u regelmatig mammogrammen heeft (gehad)?   *Er zijn meerdere antwoorden mogelijk* | | - via de polikliniek late effecten kindertumoren (PLEK/KLEP/LATER) - in verband met borstkanker in de familie - omdat ik ooit een knobbeltje in de borst heb gehad - andere reden, nl …………………………………………………………. - weet niet |
| **ROKEN, ALCOHOL, DRUGS** | | |
| 1. Heeft u ooit, **langer dan een jaar**, minstens één sigaret per week gerookt? | - ja - nee *🡺 ga door met vraag L8* | |
| 1. Op welke leeftijd of in welk jaar bent u begonnen met roken?   *U hoeft maar één mogelijkheid in te vullen.* | - leeftijd: … jaar of: in het jaar: ….. | |
| 1. Rookt u **momenteel gemiddeld** meer dan één sigaret per week? | - ja , …… sigaretten per week *🡺 ga door met vraag L8* - nee, maar ik heb wel ooit gerookt - nee, ik heb nooit gerookt *🡺 ga door met vraag L8* | |
| 1. Op welke leeftijd of in welk jaar bent u definitief gestopt met roken?   *U hoeft maar één mogelijkheid in te vullen.* | - leeftijd: … jaar; of: in het jaar: ….. | |
| 1. Hoeveel sigaretten rookte u gemiddeld per dag of per week het laatste jaar voordat u stopte met roken? | - … sigaretten per dag **of:** - … sigaretten per week | |
| 1. Heeft u ooit, **langer dan een jaar**, minstens één glas alcoholische drank per week gebruikt (gemiddeld over de week)? | - ja - nee | |
| 1. Drinkt u **momenteel** meer dan één glas alcoholische drank per week (gemiddeld)?   Zo ja, sinds wanneer is dit? | - ja, sinds leeftijd: … jaar of: sinds het jaar: ….. - nee *🡺 ga door met vraag L12* | |
| 1. Hoeveel glazen alcoholische drank drinkt u **momenteel** **gemiddeld** per dag doordeweeks? | …….. glazen per dag doordeweeks | |

| 1. Hoeveel glazen alcoholische drank drinkt u **momenteel** **gemiddeld** per dag in het weekend? | | | …….. glazen per dag in het weekend | | | | |
| --- | --- | --- | --- | --- | --- | --- | --- |
| 1. Heeft u ooit drugs gebruikt (zoals hasj, wiet, cocaïne, heroïne, XTC of andere drugs)? | | | - ja - nee *🡺 ga door met vraag M1* | | | | |
| 1. In de onderstaande tabel kunt u invullen welke drugs u ooit heeft gebruikt, zoals hasj, wiet, paddo’s, cocaïne, uppers, pep, speed, XTC, MDMA, GHB, LSD, NSIC, heroïne, crack of andere drugs. Zo ja, op welke leeftijd heeft u de genoemde drugs voor het eerst gebruikt? Het tweede deel van de tabel vraagt of u deze drugs **in het afgelopen jaar** meer dan 1x gebruikt heeft en zo ja, hoe vaak u deze drugs gemiddeld per maand of per jaar gebruikte in **het afgelopen jaar**. | | | | | | | |
|  | **naam drug(s)** | **hoe oud was u de eerste keer?** | **minstens 1x gebruikt in afgelopen jaar ?** | **zo ja, hoe vaak gebruikte u deze drugs in het afgelopen jaar** | | |  |
|  | ……………………………………. | .…. jaar | 🞎 nee 🞎 ja | ….. per maand | OF | … per jaar |  |
|  | ……………………………………. | .…. jaar | 🞎 nee 🞎 ja | ….. per maand | OF | … per jaar |  |
|  | ……………………………………. | .…. jaar | 🞎 nee 🞎 ja | ….. per maand | OF | … per jaar |  |
|  | ……………………………………. | .…. jaar | 🞎 nee 🞎 ja | ….. per maand | OF | … per jaar |  |
|  | ……………………………………. | .…. jaar | 🞎 nee 🞎 ja | ….. per maand | OF | … per jaar |  |
|  |  |  |  |  |  |  |  |

| **VERMOEIDHEID** | |
| --- | --- |
| **De vragen over dit onderwerp worden op 2 verschillende manieren gesteld. Lees s.v.p. eerst de toelichting voordat u de vragen invult.**  Hieronder staan 4 uitspraken, waarmee u kunt aangeven hoe u zich de **afgelopen 2 weken** heeft gevoeld. U kunt elke vraag beantwoorden door in één van de zeven hokjes een kruisje te zetten. De plaats van het kruisje geeft aan in welke mate u vindt dat de uitspraak op u van toepassing is. | |
| 1. Ik voel me moe | Ja, dat klopt □□□□□□□ nee, dat klopt niet |
| 1. Ik ben gauw moe | Ja, dat klopt □□□□□□□ nee, dat klopt niet |
| 1. Ik voel me fit | Ja, dat klopt □□□□□□□ nee, dat klopt niet |
| 1. Lichamelijk voel ik me uitgeput | Ja, dat klopt □□□□□□□ nee, dat klopt niet |
| Neem in uw gedachten **een normale week in de afgelopen maanden**: | |
| 1. Heeft u vermoeidheidsklachten? | - ja - soms - nee *🡺 ga door naar vraag N1* |
| 1. Zo ja, hoe lang bestaat de vermoeidheid al?   *U kunt dit in weken, maanden of jaren aangeven.* | …... weken, **of:** …… maanden, **of:** ….. jaren |
| 1. Is er volgens u een aanwijsbare oorzaak voor de vermoeidheid (bijv. verhuizing, verandering opleiding / werk, geboorte van een kind)? | - ja, nl. ......................................................................................................   …………………………………………………………………………..   - nee - weet niet |

| **LICHAMELIJKE ACTIVITEITEN** | | | | | |
| --- | --- | --- | --- | --- | --- |
| **De volgende vragen gaan over dagelijkse bezigheden.**   1. Wordt u door uw gezondheid de **afgelopen 4 weken** beperkt bij deze bezigheden. Zo ja, in welke mate? | | | | | |
|  |  | **Ja, heel erg beperkt** | **Ja, een beetje beperkt** | **Nee, helemaal niet beperkt** |  |
|  | 1. **Forse inspanning**   (zoals hardlopen, zware voorwerpen tillen, inspannend sporten) | 🞎 | 🞎 | 🞎 |  |
|  | 1. **Matige inspanning**   (zoals het verplaatsen van een tafel, fstofzuigen, fietsen) | 🞎 | 🞎 | 🞎 |  |
|  | 1. Tillen of boodschappentas dragen | 🞎 | 🞎 | 🞎 |  |
|  | 1. **Een paar** trappen oplopen | 🞎 | 🞎 | 🞎 |  |
|  | 1. **Eén** trap oplopen | 🞎 | 🞎 | 🞎 |  |
|  | 1. Buigen, knielen, of bukken | 🞎 | 🞎 | 🞎 |  |
|  | 1. **Meer dan een kilometer** lopen | 🞎 | 🞎 | 🞎 |  |
|  | 1. **Een halve kilometer** lopen | 🞎 | 🞎 | 🞎 |  |
|  | 1. **Honderd meter** lopen | 🞎 | 🞎 | 🞎 |  |
|  | 1. Uzelf wassen en aankleden | 🞎 | 🞎 | 🞎 |  |
|  |  |  |  |  |  |

| 1. In hoeverre heeft uw lichamelijke gezondheid of hebben uw emotionele problemen u de **afgelopen 4 weken** belemmerd in uw normale sociale bezigheden met gezin, vrienden, buren of anderen? | - helemaal niet - enigszins - nogal - veel - heel erg veel |
| --- | --- |
| 1. **Hoe vaak** hebben uw lichamelijke gezondheid of emotionele problemen gedurende de **afgelopen 4 weken** uw sociale activiteiten (zoals bezoek aan vrienden of naaste familieleden) belemmerd? | - voortdurend - meestal - soms - zelden - nooit |
| 1. Hoeveel pijn had u de **afgelopen 4 weken**? | - geen - heel licht - licht - nogal - ernstig - heel ernstig |
| 1. In welke mate heeft pijn u de **afgelopen 4 weken** belemmerd bij uw normale werkzaamheden (zowel werk buitenshuis als huishoudelijk werk)? | - helemaal niet - een klein beetje - nogal - veel - heel erg veel |

| 1. Kunt u in onderstaande tabel aangeven hoeveel uur u ongeveer, in **de afgelopen zomer en winter**,   per week aan de volgende activiteiten heeft besteed?   - *Het gaat hier om activiteiten buiten uw eventuele (betaalde) werk.* - *Vul ‘0’ in, indien een soort activiteit niet van toepassing is.* - *Rond ½-uren af naar boven (dus 1½ uur wordt 2 uur).* | | | | | |
| --- | --- | --- | --- | --- | --- |
|  | **soort activiteit in het afgelopen jaar** | | **aantal uren per week** | |  |
|  |  |  | **in de zomer** | **in de winter** |  |
|  | 1. wandelen (incl. naar werk, boodschappen en vrije tijd) | | **…** uur | **…** uur |  |
|  | 1. fietsen (incl. naar werk, boodschappen en vrije tijd) | | **…** uur | **…** uur |  |
|  | 1. tuinieren | | **…** uur | **…** uur |  |
|  | 1. klussen/doe-het-zelven | | **…** uur | **…** uur |  |
|  | 1. sport en andere lichaamsbeweging (bv. zwemmen, joggen, tennissen, dans) | | **…** uur | **…** uur |  |
|  | 1. huishoudelijk werk (bv. de was, schoonmaken, koken, zorg kinderen) | | **…** uur | **…** uur |  |
|  | | | | | |
| 1. In welke groep deelt u uw werk in het **afgelopen jaar**   in wat betreft lichaamsbeweging? | | - hoofdzakelijk zittend (bv. bureauwerk) - staand, soms lopend (bv. winkel, horeca, kappersbedrijf) - lopend met lichamelijke belasting (bv. verpleging) - zwaar lichamelijk werk (bv. schoonmaakwerk, werk op boerderij of tuinderij) - niet van toepassing (bv. pensioen of arbeidsongeschikt) | | | |

| 1. Wilt u in onderstaand schema invullen welke sport(en) u **in de loop van uw leven** hebt beoefend, of het in wedstrijdverband was,   hoeveel uur per week u aan die sport besteedde, en op welke leeftijd u dit deed?   - - - *Het gaat hier om activiteiten buiten uw eventuele (betaalde) werk.*     - *Rond ½-uren af naar boven (dus 1 ½ uur wordt 2 uur).*     - *Bij een verandering in wedstrijdniveau of het aantal uren per week dat u een bepaalde sport beoefende dient u een nieuwe regel te gebruiken.*     - *Als u een sport momenteel nog beoefent, vul dan uw huidige leeftijd in als eindleeftijd.* | | | | | | | | |
| --- | --- | --- | --- | --- | --- | --- | --- | --- |
|  |  | **sport** | **wedstrijd** | | **hoeveel uur**  **per week** | **leeftijd** | |  |
|  |  |  | **nee** | **ja** |  | **van** | **tot** |  |
|  | 1 |  | 🞎 | 🞎 | … uur | … jr | … jr |  |
|  | 2 |  | 🞎 | 🞎 | … uur | … jr | … jr |  |
|  | 3 |  | 🞎 | 🞎 | … uur | … jr | … jr |  |
|  | 4 |  | 🞎 | 🞎 | … uur | … jr | … jr |  |
|  | 5 |  | 🞎 | 🞎 | … uur | … jr | … jr |  |
|  | 6 |  | 🞎 | 🞎 | … uur | … jr | … jr |  |
|  | 7 |  | 🞎 | 🞎 | … uur | … jr | … jr |  |
|  | 8 |  | 🞎 | 🞎 | … uur | … jr | … jr |  |
|  |  |  |  |  |  |  |  |  |

| **BROERS/ ZUSSEN VOOR DE CONTROLEGROEP** | |
| --- | --- |
| 1. Heeft u één of meerdere (half)broers of (half)zussen? | - nee *🡺 ga naar vraag O1* - ja, ik heb ….. broer(s)/ halfbroer(s) (svp aantal invullen) - ja, ik heb ….. zus(sen)/ halfzus(sen) (svp aantal invullen) |
| *Om het huidige vragenlijst-onderzoek goed te kunnen uitvoeren, is het belangrijk dat uw gegevens vergeleken worden met gegevens van personen die in het verleden niet voor kinderkanker behandeld zijn. Broers en zussen zijn daar erg geschikt voor. U heeft aangegeven dat u broers of zussen heeft. Met de volgende vraag willen we u daarom vragen of we hen mogen benaderen om ook deze vragenlijst in te vullen.* ***Het invullen van deze vraag is niet verplicht****. Bovendien, als u ons toestemming geeft om (één van) uw broers of zussen te benaderen, zullen zij vervolgens zelf kunnen besluiten of zij WEL OF NIET willen deelnemen aan dit vragenlijst-onderzoek.* | |
| 1. Geeft u ons toestemming om uw (half)broer(s) en/of (half)zus(sen) een uitnodiging te sturen voor deelname aan dit vragenlijst-onderzoek? | - ja - nee *🡺 ga naar vraag O1* |
| De gegevens van mijn (half)broers of (half)zussen, die mogen worden benaderd, zijn:     1. Naam: …………………………………………………………..……..….….… Geslacht: m / v *     Adres: ……………………………………………………………..……..…………..………..…………    Postcode: …………………………. Woonplaats: ………............………………..….………………  Geboortedatum: …………………. Evt. emailadres/ telefoonnummer: ……..……………………     1. Naam: …………………………………………………………..……..….….… Geslacht: m / v *     Adres: ……………………………………………………………..……..…………..………..…………    Postcode: …………………………. Woonplaats: ………............………………..….………………  Geboortedatum: …………………. Evt. emailadres/ telefoonnummer: ……..……………………       1. Naam: …………………………………………………………..……..….….… Geslacht: m / v *     Adres: ……………………………………………………………..……..…………..………..…………    Postcode: …………………………. Woonplaats: ………............………………..….………………  Geboortedatum: …………………. Evt. emailadres/ telefoonnummer: ……..…………………… | |

**TOT SLOT**

| 1. Heeft u de vragen in deze vragenlijst zelf ingevuld?   *Dit geldt niet voor de vragen die gaan over uw familie of geboorte.* | - ja, ik heb de vragenlijst alleen ingevuld *🡺 ga door naar vraag O9* - nee, ik heb de vragen samen met iemand anders ingevuld - nee, iemand anders heeft de vragenlijst ingevuld |
| --- | --- |
| 1. Zo nee, (met) wie was dat? 2. Indien we naar aanleiding van deze vragenlijst nog vragen hebben, vindt u het dan goed dat we contact met u opnemen? Zo ja, kunt u dan s.v.p. uw telefoonnummer en/of uw email adres invullen? | - ouder (s) - broer/ zus - mijn partner/ echtgenoot - een vriend of vriendin - verzorger/ persoonlijk begeleider - anders namelijk ……………………………………… - nee, ik wil niet dat u nog contact met mij opneemt over deze vragenlijst - ja, u mag contact met mij opnemen over deze vragenlijst   telefoonnummer ……………..………………………………..  email adres …………………………………………………….. |
|  |  |

| **Ruimte voor aanvullende opmerkingen** |
| --- |

**
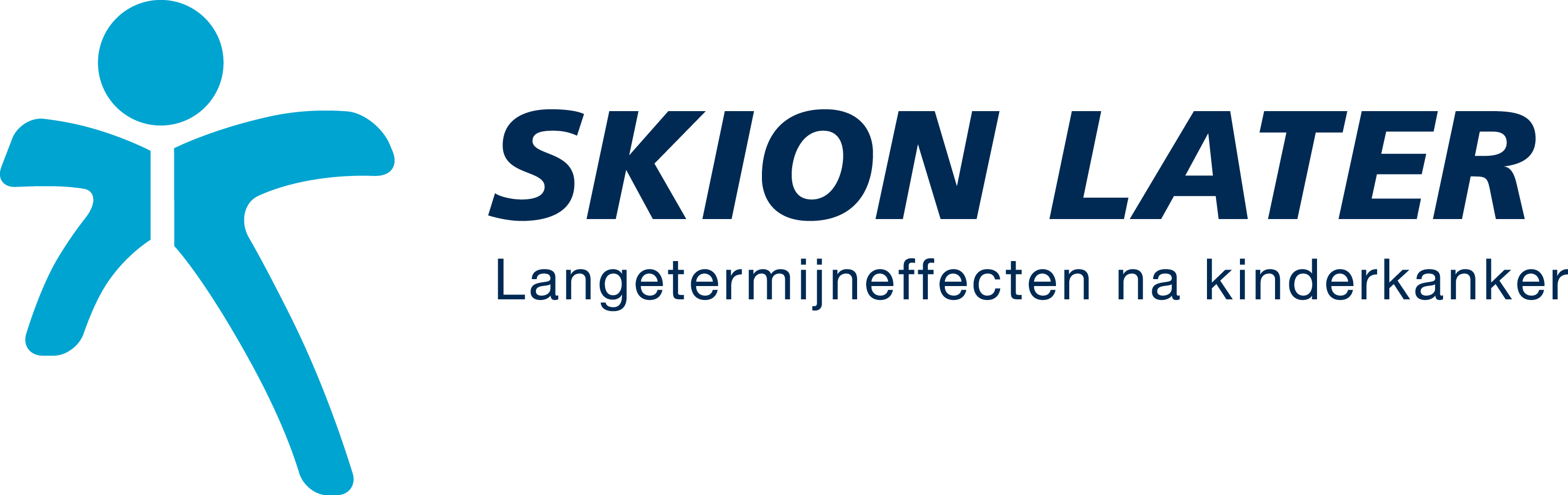
**

LATER VRAGENLIJST voor mannen

versie A4 MAN - papier │ 20 maart 2013

VRAGENLIJST

| LATER nummer  Datum invullen vragenlijst: | …………………………..……….…………..  …………………………..……….………….. |
| --- | --- |

**TOELICHTING BIJ HET INVULLEN VAN DE VRAGENLIJST**

- Het is de bedoeling dat deze vragenlijst ingevuld wordt door de persoon aan wie de vragenlijst is gericht. Het gaat om uw ***eigen*** antwoorden. Wanneer u de vragen zelf niet kunt lezen of invullen, kan iemand anders u helpen met het invullen van de vragen. U moet wel zelf de antwoorden geven.
- Wij verzoeken u zoveel mogelijk alle vragen te beantwoorden. Soms kunt u vragen overslaan, dit wordt dan ter plaatse duidelijk aangegeven. Als er meerdere antwoorden mogelijk zijn, dan wordt dat bij elke vraag afzonderlijk vermeld. Als u het antwoord op een vraag niet meer precies weet, bijvoorbeeld een leeftijd of een datum, probeert u dan een zo goed mogelijke ***schatting*** te geven.
- Enkele vragen hebben betrekking op familieleden en op uzelf toen u nog een baby was. Indien uw ouders nog in leven zijn, kunnen zij u wellicht helpen om deze vragen te beantwoorden, bijvoorbeeld met informatie van een zwangerschapskaart of het groeiboekje van het consultatiebureau.
- Het kan voorkomen dat u in deze vragenlijst om (medische) informatie wordt gevraagd die uw behandelend arts al heeft, maar de onderzoeker nog niet. Daarom hebben we deze vragen nogmaals opgenomen in de vragenlijst.
- Als u bij een vraag te weinig ruimte heeft kunt u de rest van het antwoord vermelden bij het opmerkingen veld, op de laatste bladzijde. Vermeldt u dan s.v.p. het nummer van de vraag waar het antwoord bij hoort.

.

- Uw antwoorden worden strikt vertrouwelijk behandeld. Alle onderzoeksgegevens vallen onder de Nederlandse privacywetgeving. De gegevens worden opgeslagen met een code, dus zonder uw naam. Uw gegevens zijn voor onderzoekers dus anoniem, dat wil zeggen, niet direct te herleiden tot uw persoon.

- Probeert u zoveel mogelijk binnen het hokje te blijven. Hieronder volgen een aantal voorbeelden van verschillende vragen.

Bij sommige vragen wordt u gevraagd het juiste hokje aan te kruisen:

Voorbeeld: Heeft een week 7 dagen? 🗷 ja

🞏 nee

Als u een antwoord wilt herstellen, kunt u op de volgende manier voor het juiste antwoord een pijltje zetten:

Voorbeeld: Heeft een week 7 dagen? 🗷 ja

🗷 nee

Bij andere vragen is het de bedoeling dat u iets invult:

Voorbeeld: Hoeveel maanden heeft een jaar? ***12*** maanden

**BIJ VOORBAAT DANK VOOR HET INVULLEN VAN DE VRAGENLIJST!**

| **ALGEMEEN** | |
| --- | --- |
| 1. Wat is uw geboortedatum? | ….… - …... - ….……. |
| 1. Bij welke zwangerschapsduur bent u geboren?   *Kunt u aangeven of dit de precieze duur was of dat u het ongeveer geschat heeft* | …… weken en …… dagen   - precies - geschat |
| 1. Hoe zwaar was u bij de geboorte?   *Kunt u aangeven of dit het precieze gewicht was of dat u het ongeveer geschat heeft* | …… gram   - precies - geschat |
| 1. Wat is uw geboorteplaats? | geboorteplaats: ………………………………………………………… |
| 1. Ligt uw geboorteplaats in Nederland? | - ja *🡺 ga door met vraag A7* - nee |
| 1. In welk land bent u geboren? | land: ……………………..………………………. |
| 1. Welke nationaliteit(en) heeft u? | - - Nederlands   - anders, namelijk …………………………… |
| 1. Hoeveel broers en zussen heeft u (gehad) met dezelfde   biologische ouders als u? | ….. broers  …... zussen   - - onbekend |
| 1. Bent u deel van een tweeling/meerling? | - - nee   - ja, ik heb een tweelingbroer (gehad)   - ja, ik heb een tweelingzus (gehad)   - ja, ik ben deel van een drie-of vierling   - onbekend |
| 1. Wat is uw huidige woonsituatie? | - - bij ouder(s) wonend   - alleenwonend   - samenwonend met partner en/of kinderen   - samenwonend met anderen, nl. ………………………………….   - niet-zelfstandig wonend, buiten het gezin ( bijvoorbeeld tehuis voor gehandicapten, begeleid zelfstandig wonen)   - anders, namelijk: ……………………………………………………     …………………………………………………… |
| 1. Wat is uw huidige burgerlijke staat? | - - - ongehuwd, geen (vaste) relatie     - ongehuwd, wel (vaste) relatie     - gehuwd     - gescheiden     - weduwe     - anders, namelijk: …………………………………………………… |
| 1. Welke van de volgende situaties is op u van toepassing?   *Er zijn meerdere antwoorden mogelijk.* | - ik heb betaald werk, ik werk ……uur per week als …………………….(beroep) sinds het jaar ..….. - ik ben zelfstandig ondernemer, ik werk circa……uur per week als ……………………(beroep) sinds het jaar ..….. - ik volg onderwijs/studeer sinds het jaar ..….. - ik ben fulltime huisvrouw sinds het jaar ..….. - ik ben werkzoekende en/of ontvang wachtgeld (RWW, WW, WWV) sinds het jaar ..….. - ik ben arbeidsongeschikt, ik ben voor …. % afgekeurd en ontvang sinds het jaar ..….. een WAO of WIA uitkering - ik ontvang een Wajong uitkering, voor ….. % sinds het jaar ..….. - ik ontvang een bijstandsuitkering sinds het jaar ..….. - ik werk niet en ontvang geen uitkering sinds het jaar ..….. - anders, namelijk …………………………………..………………….   …………………………………..…………………. |

| 1. Volgt u op dit moment een opleiding? | - ja - nee *🡺 ga door met vraag A15* |
| --- | --- |
| 1. Welke opleiding volgt u op dit moment? | - voortgezet speciaal onderwijs - praktijkonderwijs - VMBO - middelbaar beroepsonderwijs (MBO, MLO, MEAO, MTS) - HAVO - VWO, Gymnasium - hoger beroeps onderwijs (HBO, HTS, HEAO) - universiteit - anders, namelijk …………………………………..…………………. |
| 1. Wat is de hoogst genoten opleiding die u heeft afgerond? | - regulier basisonderwijs (lagere school) - speciaal basisonderwijs - voortgezet speciaal onderwijs - praktijkonderwijs - lager beroepsonderwijs (LBO, LTS, LEAO, huishoudschool) - VMBO/ MAVO - middelbaar beroepsonderwijs (MBO, MLO, MEAO, MTS) - HAVO - VWO, Gymnasium - hoger beroeps onderwijs (HBO, HTS, HEAO) - universiteit - anders, namelijk …………………………………..…………………. |
| 1. Wat is de hoogste vorm van onderwijs die uw vader heeft afgerond? | - regulier basisonderwijs (lagere school) - speciaal basisonderwijs - voortgezet speciaal onderwijs - praktijkonderwijs - lager beroepsonderwijs (LBO, LTS, LEAO, huishoudschool) - VMBO/ MAVO - middelbaar beroepsonderwijs (MBO, MLO, MEAO, MTS) - HAVO - VWO, Gymnasium - hoger beroeps onderwijs (HBO, HTS, HEAO) - universiteit - anders, namelijk …………………………………..………………… |
| 1. Wat is de hoogste vorm van onderwijs die uw moeder heeft afgerond? | - regulier basisonderwijs (lagere school) - speciaal basisonderwijs - voortgezet speciaal onderwijs - praktijkonderwijs - lager beroepsonderwijs (LBO, LTS, LEAO, huishoudschool) - VMBO/ MAVO - middelbaar beroepsonderwijs (MBO, MLO, MEAO, MTS) - HAVO - VWO, Gymnasium - hoger beroeps onderwijs (HBO, HTS, HEAO) - universiteit - anders, namelijk …………………………………..…………………. |
| 1. Wat is het beroep van uw ouders?   *Indien uw ouders niet meer werken of niet meer in leven zijn, kunt u het beroep invullen dat ze als laatste hebben gehad. Indien uw ouders geen betaald werk hebben verricht kunt u “niet van toepassing” kiezen.* | beroep vader: …………………………………..………………….   - niet van toepassing   beroep moeder: …………………………………..………………….   - niet van toepassing |
| 1. Hoe lang bent u op dit moment? | lengte …………….. cm |
| 1. Wat is uw huidige gewicht? | gewicht ……………. kg |
| 1. Hoe lang was uw moeder als jonge vrouw (maximale lengte)? | lengte …………….. cm   - onbekend |
| 1. Hoe lang was uw vader als jonge man (maximale lengte)? | lengte …………….. cm   - onbekend |

| **MEDISCHE INFORMATIE** | | | | | | | | | | | | | | | | |
| --- | --- | --- | --- | --- | --- | --- | --- | --- | --- | --- | --- | --- | --- | --- | --- | --- |
| 1. U bent in uw jeugd behandeld voor kinderkanker of een aanverwante aandoening. Heeft u daarna nog een andere vorm van kanker, leukemie of een tumor gekregen of heeft u deze nu? (*deze vraag betreft een nieuwe, andere vorm van kanker; als dit een terugkeren van de eerdere kindertumor of leukemie was, mag u deze vraag met NEE beantwoorden en hoeft u het schema B2 niet in te vullen*)  - ja - nee *🡺 ga door met vraag B3* | | | | | | | | | | | | | | | | |
| 1. Kunt u dan voor elke nieuwe tumor in het onderstaande schema aangeven in welk orgaan/deel van het lichaam deze zich bevond, welk soort tumor het was en in welk jaar of op welke leeftijd de diagnose gesteld werd? | | | | | | | | | | | | | | | | |
|  |  | **orgaan/lichaamsdeel** | **soort tumor** | | | | **leeftijd bij diagnose** | | **OF** | | **jaar van diagnose** | | | |  | |
|  | bijvoorbeeld | Huid van de onderbuik links | melanoom | | | | 25 jaar | |  | | / | | | |  | |
|  | 1 |  |  | | | |  | |  | |  | | | |  | |
|  | 2 |  |  | | | |  | |  | |  | | | |  | |
|  | 3 |  |  | | | |  | |  | |  | | | |  | |
|  | 4 |  |  | | | |  | |  | |  | | | |  | |
| 1. Zou u in onderstaand schema willen invullen of u nu of in het verleden één of meer van de onderstaande aandoeningen heeft gehad? Zo ja, kunt u dan s.v.p. ook schatten hoe oud u was bij de diagnose of in welk jaar dat was en of u hier **op dit moment** medicijnen voor gebruikt? *Op de stippellijnen kunt u toelichting geven over de precieze aandoening, Indien u voor één of meerdere aandoeningen in schema B3 heeft aangegeven dat u nu medicatie gebruikt, kunt u dan voor elk van deze aandoeningen bij B4 invullen hoe dat medicijn heet en op welke leeftijd of in welk jaar u begonnen bent met het gebruik?* | | | | | | | | | | | | | | | | |
|  | | | | **gehad of nu aanwezig?** | | **leeftijd bij diagnose** | | **OF** | | **jaar van diagnose** | | **medicijnen**  **op dit moment?** | | | | |
| 1. Hartinfarct | | | | 🞎 nee | 🞎 ja | ………… jr | | of | | …………… | | 🞎 nee | | 🞎 ja | | |
| 1. Pijn op de borst (bij inspanning en/of rust) | | | | 🞎 nee | 🞎 ja | ………… jr | | of | | …………… | | 🞎 nee | | 🞎 ja | | |
| 1. Hartklepafwijking | | | | 🞎 nee | 🞎 ja | ………… jr | | of | | …………… | | 🞎 nee | | 🞎 ja | | |
| 1. Ontsteking van het hartzakje (pericarditis) | | | | 🞎 nee | 🞎 ja | ………… jr | | of | | …………… | | 🞎 nee | | 🞎 ja | | |
| ***(1^e^ vervolg vraag B3 aandoeningen)*** | | | | **gehad of nu aanwezig?** | | **leeftijd bij diagnose** | | **OF** | | **jaar van diagnose** | | **medicijnen op dit moment?** | | | | |
| 1. Zwakke hartspier (cardiomyopathie) | | | | 🞎 nee | 🞎 ja | ………… jr | | of | | …………… | | 🞎 nee | | 🞎 ja | | |
| 1. Hartfalen | | | | 🞎 nee | 🞎 ja | ………… jr | | of | | …………… | | 🞎 nee | | 🞎 ja | | |
| 1. Hartritmestoornissen | | | | 🞎 nee | 🞎 ja | ………… jr | | of | | …………… | | 🞎 nee | | 🞎 ja | | |
| 1. Aangeboren hartafwijking nl…………………………………………………… | | | | 🞎 nee | 🞎 ja | ………… jr | | of | | …………… | | 🞎 nee | | 🞎 ja | | |
| 1. Andere hartziekte, nl.: …………………………..……………………………… | | | | 🞎 nee | 🞎 ja | ………… jr | | of | | …………… | | 🞎 nee | | 🞎 ja | | |
| 1. Beroerte (CVA / herseninfarct/hersenbloeding) | | | | 🞎 nee | 🞎 ja | ………… jr | | of | | …………… | | 🞎 nee | | 🞎 ja | | |
| 1. TIA (beroerte binnen 24 uur hersteld) | | | | 🞎 nee | 🞎 ja | ………… jr | | of | | …………… | | 🞎 nee | | 🞎 ja | | |
| 1. Vaatafwijkingen, nl.: ……………………………………………….…………… | | | | 🞎 nee | 🞎 ja | ………… jr | | of | | …………… | | 🞎 nee | | 🞎 ja | | |
| 1. En aandoening die een verhoogde stollingsneiging (trombose) veroorzaakt   Indien ja, welke? 🞎 proteïne C deficiëntie  🞎 proteïne S deficiëntie  🞎 factor V Leiden mutatie  🞎 overig nl……………………………………………. | | | | 🞎 nee | 🞎 ja | ………… jr  ………… jr  ………… jr  ………… jr | | of | | ……………  ……………  ……………  …………… | | 🞎 nee  🞎 nee  🞎 nee  🞎 nee | 🞎 ja  🞎 ja  🞎 ja  🞎 ja | | | |
| 1. Hoge bloeddruk (hypertensie) | | | | 🞎 nee | 🞎 ja | ………… jr | | of | | …………… | | 🞎 nee | 🞎 ja | | | |
| 1. Hoog cholesterol | | | | 🞎 nee | 🞎 ja | ………… jr | | of | | …………… | | 🞎 nee | 🞎 ja | | | |
| 1. Problemen met de maag of darmen | | | | 🞎 nee | 🞎 ja | ………… jr | | of | | …………… | | 🞎 nee | 🞎 ja | | | |
| ***(2^e^ vervolg vraag B3 aandoeningen)*** | | | | **gehad of nu aanwezig?** | | **leeftijd bij diagnose** | | **OF** | | **jaar van diagnose** | | **medicijnen op dit moment?** | | | | |
| 1. Longaandoeningen, nl.: ………………………………………………………... | | | | 🞎 nee | 🞎 ja | ………… jr | | of | | …………… | | 🞎 nee | | | | 🞎 ja |
| 1. Heeft u in het **afgelopen** jaar een periode gehad waarin u meer dan 6 weken aaneengesloten hoestte? | | | | 🞎 nee | 🞎 ja | niet van toepassing (n.v.t) | | | | | | 🞎 nee | | | | 🞎 ja |
| 1. Heeft u meer dan 3x per jaar een infectie van de luchtwegen? | | | | 🞎 nee | 🞎 ja | n.v.t. | | | | | | 🞎 nee | | | | 🞎 ja |
| 1. Heeft u wel eens last (gehad) van urineweginfecties met koorts (nierbekken- ontsteking)?   Indien ja, hoe vaak ? 🞎 1 keer  🞎 2-5 keer  🞎 meer dan 5 keer | | | | 🞎 nee | 🞎 ja | n.v.t. | | | | | | n.v.t. | | | | |
| 1. Heeft u andere problemen met uw nieren (bijvoorbeeld slecht werkende nieren, nierstenen, teveel eiwit in de urine, cystes)? | | | | 🞎 nee | 🞎 ja | n.v.t. | | | | | | n.v.t. | | | | |
| Indien ja, welke …………………………………………………………………    ………………………………………………………………… | | | |  |  | ………… jr  ………… jr | | of  of | | ……………  …………… | | 🞎 nee  🞎 nee | | | | 🞎 ja  🞎 ja |
| 1. Problemen met de bijnieren, nl ………………………………………………. | | | | 🞎 nee | 🞎 ja | ………… jr | | of | | …………… | | 🞎 nee | | | | 🞎 ja |
| 1. Leverproblemen, nl …………………………………………………………… | | | | 🞎 nee | 🞎 ja | ………… jr | | of | | …………… | | 🞎 nee | | | | 🞎 ja |
| 1. Problemen met het bewegingsapparaat (bijvoorbeeld arm/been/ elleboog/knie) nl : ………………….………................................................. | | | | 🞎 nee | 🞎 ja | ………… jr | | of | | …………… | | 🞎 nee | | | | 🞎 ja |
| 1. Suikerziekte (diabetes mellitus) | | | | 🞎 nee | 🞎 ja | ………… jr | | of | | …………… | | 🞎 nee | | | | 🞎 ja |
| ***(3^e^ vervolg vraag B3 aandoeningen)*** | | | | **gehad of nu aanwezig?** | | **leeftijd bij diagnose** | | **OF** | | **jaar van diagnose** | | **medicijnen**  **op dit moment?** | | | | |
| 1. Epilepsieaanvallen | | | | 🞎 nee | 🞎 ja | ………… jr | | of | | …………… | | 🞎 nee | | | | 🞎 ja |
| 1. Is er bij u door een oogarts staar geconstateerd? | | | | 🞎 nee | 🞎 ja | ………… jr | | of | | …………… | | n.v.t. | | | | |
| 1. Heeft u een gehoorapparaat? | | | | 🞎 nee | 🞎 ja | ………… jr | | of | | …………… | | n.v.t. | | | | |
| 1. Heeft u last van oorsuizen? | | | | 🞎 nee | 🞎 ja | ………… jr | | of | | …………… | | 🞎 nee | | | | 🞎 ja |
| 1. Is er bij u sprake (geweest) van verminderde lengtegroei (korte lichaamslengte)? | | | | 🞎 nee | 🞎 ja | ………… jr | | of | | …………… | | 🞎 nee | | | | 🞎 ja |
| 1. Verminderde schildklierfunctie (hypothyreoïdie) | | | | 🞎 nee | 🞎 ja | ………… jr | | of | | …………… | | 🞎 nee | | | | 🞎 ja |
| 1. Verhoogde schildklierfunctie (hyperthyreoïdie) | | | | 🞎 nee | 🞎 ja | ………… jr | | of | | …………… | | 🞎 nee | | | | 🞎 ja |
| 1. Schildklierknobbel (schildkliernodus) | | | | 🞎 nee | 🞎 ja | ………… jr | | of | | …………… | | 🞎 nee | | | | 🞎 ja |
| 1. Andere schildklieraandoening, nl ………………………………………........ | | | | 🞎 nee | 🞎 ja | ………… jr | | of | | …………… | | 🞎 nee | | | | 🞎 ja |
| 1. Ander probleem met hormonenregulatie, nl …………………………………   …………………………………………………………………………………… | | | | 🞎 nee | 🞎 ja | ………… jr | | of | | …………… | | 🞎 nee | | | | 🞎 ja |
| 1. Andere aandoening, nl ………………………………………………………..   …………………………………………………………………………………… | | | | 🞎 nee | 🞎 ja | ………… jr | | of | | …………… | | 🞎 nee | | | | 🞎 ja |

| 1. Wilt u aangeven welke medicijnen u op dit moment gebruikt voor de aandoeningen genoemd bij vraag B3 en op welke leeftijd of in welk jaar u begonnen bent met het gebruik? Als u niet weet hoe het medicijn heet, vult u dan s.v.p. de naam van de aandoening uit B3 in gevolgd door "weet niet". | | | | | | | | | | |
| --- | --- | --- | --- | --- | --- | --- | --- | --- | --- | --- |
|  | **naam medicijn** | **aandoening** | | | **begonnen op leeftijd** | | | **of** | **in het jaar** |  |
|  | bijvoorbeeld Thyroxine (Thyrax) | Verminderde schildklierfunctie | | |  | | |  | 1986 |  |
|  | 1 |  | | |  | | |  |  |  |
|  | 2 |  | | |  | | |  |  |  |
|  | 3 |  | | |  | | |  |  |  |
|  | 4 |  | | |  | | |  |  |  |
|  | 5 |  | | |  | | |  |  |  |
|  | 6 |  | | |  | | |  |  |  |
|  | 7 |  | | |  | | |  |  |  |
|  | 8 |  | | |  | | |  |  |  |
| 1. Gebruikt u **op dit moment** naast de medicijnen die u eventueel hierboven heeft genoemd, nog andere medicijnen of injecties? Denk daarbij bijvoorbeeld aan pijnstillers die u vaker dan 1x per week gebruikt (bijv. aspirine, paracetamol, Ibuprofen) of hormonen. | | | - ja - nee *🡺 ga door met vraag B7* | | | | | | | |
| 1. Zo ja, welke medicijnen zijn dit en op welke leeftijd of in welk jaar bent u begonnen met het nemen van deze medicijnen? | | | | | | | | | | |
|  | **naam medicijn** | | | **begonnen op leeftijd** | | **of** | **in het jaar** | | |  |
|  | 1 | | |  | |  |  | | |  |
|  | 2 | | |  | |  |  | | |  |

|  | ***(vervolg vraag B6 andere medicijnen)***  **naam medicijn** | **begonnen op leeftijd** | | | **of** | | **in het jaar** | |  |
| --- | --- | --- | --- | --- | --- | --- | --- | --- | --- |
|  | 3 |  | | |  | |  | |  |
|  | 4 |  | | |  | |  | |  |
|  | 5 |  | | |  | |  | |  |
|  | 6 |  | | |  | |  | |  |
|  | 7 |  | | |  | |  | |  |
|  | 8 |  | | |  | |  | |  |
| B7 Heeft u ooit de volgende behandeling of operatie ondergaan? | | **gehad?** | | **leeftijd bij behandeling/ operatie** | | **of:** | | **jaar van behandeling/ operatie** |  |
| a. Heeft u ooit een vervanging van een hartklep gehad? | | 🞎 nee | 🞎 ja | ………… jr | | of | | …………… |  |
| b. Heeft u ooit een andere operatie aan uw hart gehad (inclusief  dotteren)? nl………………………………………………………. | | 🞎 nee | 🞎 ja | ………… jr | | of | | …………… |  |
| c. Heeft u ooit een pacemaker/ ICD gekregen? | | 🞎 nee | 🞎 ja | ………… jr | | of | | …………… |  |
| d. Heeft u ooit een orgaantransplantatie ondergaan?    Zo ja, welk orgaan ………………………………………………… | | 🞎 nee | 🞎 ja | ………… jr | | of | | …………… |  |
| e. Is er bij u ooit een heel orgaan of een arm of been verwijderd?  Zo ja, welk orgaan/ ledemaat (arm/been) ……………………… | | 🞎 nee | 🞎 ja | ………… jr | | of | | …………… |  |
| 1. Gaat u ermee akkoord dat wij bij uw huisarts/specialist over de door u bij de vragen B2 en B3 gerapporteerde ziekten/aandoeningen eventueel aanvullende informatie opvragen?  - nee *🡺 ga door naar de vraag op de volgende bladzijde* - ja   **Zo ja**, wilt u dan hier uw handtekening zetten? ……………………………………………………………………………..  **Zo ja**, wilt u dan zo vriendelijk zijn hieronder de adresgegevens van uw huisarts te vermelden?  Naam huisarts …………………………………………………………………………………………….  Adres …………………………………………………………………………………………….    Postcode/Woonplaats …………………………………………………………………………………………….  Telefoon …………………………………………………………………………………………….  **Zo ja**, wilt u dan zo vriendelijk zijn hieronder per door u gerapporteerde ziekte/aandoening de adresgegevens van de betreffende specialist te vermelden?  *Indien u meer ruimte nodig heeft kunt u verdere namen en adressen vermelden bij het opmerkingenveld op de laatste pagina van de vragenlijst*  1. Soort ziekte/aandoening: …………………………………………………………………………………………….  Naam specialist …………………………………………………………………………………………….  Ziekenhuis en afdeling …………………………………………………………………………………………….  2. Soort ziekte/aandoening: …………………………………………………………………………………………….  Naam specialist …………………………………………………………………………………………….  Ziekenhuis en afdeling …………………………………………………………………………………………….  3. Soort ziekte/aandoening: …………………………………………………………………………………………….  Naam specialist …………………………………………………………………………………………….  Ziekenhuis en afdeling ……………………………………………………………………………………………. | | | | | | | | | |

| **ZIEKTES IN DE FAMILIE** | | | |  | | | | |
| --- | --- | --- | --- | --- | --- | --- | --- | --- |
| 1. Zijn er mensen in uw familie die kanker hebben gehad?     *Met familie bedoelen we uw biologische vader/ moeder/ grootvader/ grootmoeder/ broer(s)/ zus(sen) en uw kind(eren). Graag zowel het kalenderjaar als de leeftijd bij diagnose invullen. U mag ook een schatting geven.* | | | | - ja *🡺 vul s.v.p. de onderstaande tabel in* - nee *🡺 ga door naar vraag D2* - weet niet *🡺 ga door naar vraag D2* | | | | |
|  |  | **relatie tot familielid**  **(bijv. broer, moeder)** | **soort kanker** | **kalenderjaar van diagnose** | **leeftijd bij diagnose** | **indien in leven,**  **huidige leeftijd** | **indien overleden,**  **leeftijd bij overlijden** |  |
|  | 1 |  |  |  |  |  |  |  |
|  | 2 |  |  |  |  |  |  |  |
|  | 3 |  |  |  |  |  |  |  |
|  | 4 |  |  |  |  |  |  |  |
| 1. Zijn er mensen in de familie die een hart- en vaatziekte hebben (gehad), bijvoorbeeld een hartinfarct, beroerte, hersenbloeding, hartfalen, familiaire hypercholesterolemie, hoge bloeddruk, familiaire ritmestoornis, stollingsziekte of suikerziekte/diabetes? | | | | - ja *🡺 vul s.v.p. de onderstaande tabel in* - nee *🡺 ga door naar vraag E1* - weet niet *🡺 ga door naar vraag E1* | | | | |
|  |  | **relatie tot familielid**  **(bijv. broer, moeder)** | **soort ziekte** | **kalenderjaar van diagnose** | **leeftijd bij diagnose** | **indien in leven,**  **huidige leeftijd** | **indien overleden,**  **leeftijd bij overlijden** |  |
|  | 1 |  |  |  |  |  |  |  |
|  | 2 |  |  |  |  |  |  |  |
|  | 3 |  |  |  |  |  |  |  |
|  | 4 |  |  |  |  |  |  |  |
|  |  |  |  |  |  |  |  |  |

| **KEURING, BAAN en PSYCHOSOCIALE HULP** |  |
| --- | --- |
| 1. Heeft u wel eens problemen gehad bij een keuring in verband met   uw ziektegeschiedenis? | - nee *🡺 ga door met vraag E3* - ja - niet van toepassing *🡺 ga door met vraag E3* |
| 1. Zo ja, in welk jaar was dat ongeveer en op welke wijze? | jaar …………….  probleem: ………………………………………………………………………… |
| 1. Heeft u wel eens problemen gehad bij het vinden van een baan in verband met uw ziektegeschiedenis? | - nee *🡺 ga door met vraag F1* - ja |
| 1. Zo ja, in welk jaar was dat ongeveer en op welke wijze? | jaar …………….  probleem: …………………………………………………………………………. |
| 1. Heeft u wel eens problemen gehad bij het verkrijgen van een verzekering in verband met uw ziektegeschiedenis? | - nee *🡺 ga door met vraag G1* - ja |
| 1. Zo ja, in welk jaar was dat ongeveer en op welke wijze? | jaar …………….  probleem: ………………………………………………………………………… |

| 1. Heeft u ooit psychosociale hulp ontvangen of heeft u op dit moment hulp? | | | - nee *🡺 ga door met vraag H1* - ja | | |
| --- | --- | --- | --- | --- | --- |
| 1. Zo ja, kunt u s.v.p. in het schema invullen wanneer u hulp kreeg/krijgt, van wie u hulp kreeg, de reden voor de hulp en hoeveel maanden de hulp geduurd heeft? | | | | | |
|  | **wanneer (jaren)** | **soort hulpverlener (maatschappelijk werk, psycholoog, psychiater)** | **reden** | **duur** |  |
|  | Van 1995 tot 1996 | Psycholoog | Leren omgaan met ziekteverleden | 8 maanden |  |
|  | 1. van ……… tot ………… |  |  | ………. maanden |  |
|  | 1. van ……… tot ………… |  |  | ………. maanden |  |
|  | 1. van ……… tot ………… |  |  | ………. maanden |  |
|  |  |  |  |  | |

| **PUBERTEIT** |  |
| --- | --- |
| 1. Op welke leeftijd kwam u in de puberteit (kreeg u oksel- en schaambeharing)? | - leeftijd: ….. jaar 🡺 *ga door met vraag H3* - weet ik niet |
| 1. Kunt u aangeven hoe oud u ongeveer was toen u in de puberteit kwam? | - - jonger dan 11 jaar   - 11, 12, 13, 14 of 15 jaar   - 16 jaar of ouder |
| 1. Heeft u medicijnen gekregen om de puberteit op te wekken? | - - nee   - ja, namelijk het medicijn:…………………………….   - weet ik niet |
| **KINDEREN EN KINDERWENS** |  |
| 1. Is er voordat u behandeld werd met chemotherapie of bestraling voor de kinderkanker of aanverwante aandoening, zaad van u ingevroren? | - nee - ja |
| 1. Is een vrouw ooit zwanger geweest van u?   *Hiermee bedoelen we een positieve zwangerschapstest nadat uw partner “over tijd” was.* | - ja, aantal keren: ……………… - nee 🡺 *ga door met vraag I5* |
| 1. Heeft u biologisch eigen kinderen? | - ja, aantal: ……………… - nee 🡺 *ga door met vraag I5* |
| 1. Heeft u één of meerdere biologisch eigen kind(eren) met een aangeboren afwijking of ernstige gezondheidsproblemen?   *(Met aangeboren afwijkingen bedoelen we onder andere: hazenlip, open ruggetje, hartaandoening, enz.; met ernstige gezondheidsproblemen bedoelen we onder andere: astma, spierziekte, enz )* | - nee - ja, namelijk ……………………………………………………………………   ……………………………………………………………………. |

| 1. Heeft u (nog) een kinderwens? | - ja, mijn partner en ik zijn actief bezig om zwanger te worden 🡪 ga door met vraag 10 - ja, maar op dit moment nog niet 🡪 ga door met vraag 10 - ik heb er nog niet over nagedacht 🡪 ga door met vraag 10 - nee, niet meer 🡪 ga door met vraag 10 - nee, nooit gehad 🡺 *ga door met vraag J1*   🡪 u bent klaar met deze vragenlijst |
| --- | --- |
| 1. Heeft u ooit een vruchtbaarheidskliniek of gynaecoloog /fertiliteitstarts bezocht omdat het u en uw partner niet lukte om zwanger te worden? | - - nee   - ja |
| 1. Hebben u en/of uw partner ooit medicijnen gekregen en/of een behandeling ondergaan in verband met het uitblijven van een spontane zwangerschap? | - - nee 🡺 *ga door met vraag J1*   - ja |
| 1. Heeft u ooit medicijnen gebruikt om de kwaliteit van het sperma te verbeteren? | - - nee - ja, namelijk medicijn: …………………………. |
| 1. Heeft uw vrouwelijke partner hormoonbehandelingen ondergaan? | - - nee   - ja, aantal behandelingen … |
| 1. Hebben u en uw partner ooit de volgende behandelingen ondergaan?    - ja , namelijk ( *Deze behandelingen kunnen met of zonder hormoonbehandeling zijn ondergaan. Er zijn meerdere keuzes mogelijk*)    - kunstmatige inseminatie met eigen zaad (IUI), aantal behandelingen …….    - kunstmatige inseminatie met donorzaad (KID), aantal behandelingen …….    - IVF (in vitro fertilisatie), aantal behandelingen …….    - ICSI (intracytoplasmatische sperma injectie), aantal behandelingen …….    - Chirurgisch verkregen zaad uit de bijbal (PESA) inclusief ICSI, aantal behandelingen …….    - Chirurgisch verkregen zaad uit de zaadbal (TESE) inclusief ICSI, aantal behandelingen …….  - nee, we hebben geen van de genoemde behandelingen ondergaan | |

| **SEKSUALITEIT** |  |
| --- | --- |
| 1. Ik voel me aangetrokken tot | - vrouwen - mannen - beiden |
| 1. Ervaart u problemen op seksueel gebied? | - nee *🡺 ga door met vraag J4* - ja |
| 1. Welke problemen ervaart u?   *Er zijn meerdere antwoorden mogelijk* | - erectiestoornissen - problemen met het krijgen van een zaadlozing - seksuele behoefte/verlangen is verminderd - seksuele behoefte/verlangen is verhoogd - anders, namelijk …………………………………………………………………….     ……………………………………………………………………. |
| 1. Hoe oud was u toen u voor het eerst seksueel actief werd?   *Onder seksueel actief wordt verstaan strelen onder kleren, voorspel, masturbatie, geslachtsgemeenschap of een combinatie* | - leeftijd …….jaar - weet niet |
| 1. Het ging toen om:   *Er zijn meerdere antwoorden mogelijk* | - strelen onder kleren - voorspel - masturbatie - geslachtsgemeenschap *🡺 ga door met vraag J7* |
| 1. Heeft u ooit geslachtsgemeenschap gehad? | - nee *🡺 ga door met vraag L2* - ja |
| 1. Hoe oud was u toen u voor het eerst geslachtsgemeenschap had? | - leeftijd …….jaar - weet niet |

| **ROKEN, ALCOHOL, DRUGS** | |
| --- | --- |
| 1. Heeft u ooit, **langer dan een jaar**, minstens één sigaret per week gerookt? | - ja - nee *🡺 ga door met vraag L8* |
| 1. Op welke leeftijd of in welk jaar bent u begonnen met roken?   *U hoeft maar één mogelijkheid in te vullen.* | - leeftijd: … jaar of: in het jaar: ….. |
| 1. Rookt u **momenteel gemiddeld** meer dan één sigaret per week? | - ja , …… sigaretten per week *🡺 ga door met vraag L8* - nee, maar ik heb wel ooit gerookt - nee, ik heb nooit gerookt *🡺 ga door met vraag L8* |
| 1. Op welke leeftijd of in welk jaar bent u definitief gestopt met roken?   *U hoeft maar één mogelijkheid in te vullen.* | - leeftijd: … jaar; of: in het jaar: ….. |
| 1. Hoeveel sigaretten rookte u gemiddeld per dag of per week het laatste jaar voordat u stopte met roken? | - … sigaretten per dag **of:** - … sigaretten per week |
| 1. Heeft u ooit, **langer dan een jaar**, minstens één glas alcoholische drank per week gebruikt (gemiddeld over de week)? | - ja - nee |
| 1. Drinkt u **momenteel** meer dan één glas alcoholische drank per week (gemiddeld)?   Zo ja, sinds wanneer is dit? | - ja, sinds leeftijd: … jaar of: sinds het jaar: ….. - nee *🡺 ga door met vraag L12* |
| 1. Hoeveel glazen alcoholische drank drinkt u **momenteel** **gemiddeld** per dag doordeweeks? | …….. glazen per dag doordeweeks |

| 1. Hoeveel glazen alcoholische drank drinkt u **momenteel** **gemiddeld** per dag in het weekend? | | | …….. glazen per dag in het weekend | | | | |
| --- | --- | --- | --- | --- | --- | --- | --- |
| 1. Heeft u ooit drugs gebruikt (zoals hasj, wiet, cocaïne, heroïne, XTC of andere drugs)? | | | - ja - nee *🡺 ga door met vraag M1* | | | | |
| 1. In de onderstaande tabel kunt u invullen welke drugs u ooit heeft gebruikt, zoals hasj, wiet, paddo’s, cocaïne, uppers, pep, speed, XTC, MDMA, GHB, LSD, NSIC, heroïne, crack of andere drugs. Zo ja, op welke leeftijd heeft u de genoemde drugs voor het eerst gebruikt? Het tweede deel van de tabel vraagt of u deze drugs **in het afgelopen jaar** meer dan 1x gebruikt heeft en zo ja, hoe vaak u deze drugs gemiddeld per maand of per jaar gebruikte in **het afgelopen jaar**. | | | | | | | |
|  | **naam drug(s)** | **hoe oud was u de eerste keer?** | **minstens 1x gebruikt in afgelopen jaar ?** | **zo ja, hoe vaak gebruikte u deze drugs in het afgelopen jaar** | | |  |
|  | ……………………………………. | .…. jaar | 🞎 nee 🞎 ja | ….. per maand | OF | … per jaar |  |
|  | ……………………………………. | .…. jaar | 🞎 nee 🞎 ja | ….. per maand | OF | … per jaar |  |
|  | ……………………………………. | .…. jaar | 🞎 nee 🞎 ja | ….. per maand | OF | … per jaar |  |
|  | ……………………………………. | .…. jaar | 🞎 nee 🞎 ja | ….. per maand | OF | … per jaar |  |
|  | ……………………………………. | .…. jaar | 🞎 nee 🞎 ja | ….. per maand | OF | … per jaar |  |
|  |  |  |  |  |  |  |  |

| **VERMOEIDHEID** | |
| --- | --- |
| **De vragen over dit onderwerp worden op 2 verschillende manieren gesteld. Lees s.v.p. eerst de toelichting voordat u de vragen invult.**  Hieronder staan 4 uitspraken, waarmee u kunt aangeven hoe u zich de **afgelopen 2 weken** heeft gevoeld. U kunt elke vraag beantwoorden door in één van de zeven hokjes een kruisje te zetten. De plaats van het kruisje geeft aan in welke mate u vindt dat de uitspraak op u van toepassing is. | |
| 1. Ik voel me moe | Ja, dat klopt □□□□□□□ nee, dat klopt niet |
| 1. Ik ben gauw moe | Ja, dat klopt □□□□□□□ nee, dat klopt niet |
| 1. Ik voel me fit | Ja, dat klopt □□□□□□□ nee, dat klopt niet |
| 1. Lichamelijk voel ik me uitgeput | Ja, dat klopt □□□□□□□ nee, dat klopt niet |
| Neem in uw gedachten **een normale week in de afgelopen maanden**: | |
| 1. Heeft u vermoeidheidsklachten? | - ja - soms - nee *🡺 ga door naar vraag N1* |
| 1. Zo ja, hoe lang bestaat de vermoeidheid al?   *U kunt dit in weken, maanden of jaren aangeven.* | …... weken, **of:** …… maanden, **of:** ….. jaren |
| 1. Is er volgens u een aanwijsbare oorzaak voor de vermoeidheid (bijv. verhuizing, verandering opleiding / werk, geboorte van een kind)? | - ja, nl. ......................................................................................................   …………………………………………………………………………..   - nee - weet niet |

| **LICHAMELIJKE ACTIVITEITEN** | | | | | |
| --- | --- | --- | --- | --- | --- |
| **De volgende vragen gaan over dagelijkse bezigheden.**   1. Wordt u door uw gezondheid de **afgelopen 4 weken** beperkt bij deze bezigheden. Zo ja, in welke mate? | | | | | |
|  |  | **Ja, heel erg beperkt** | **Ja, een beetje beperkt** | **Nee, helemaal niet beperkt** |  |
|  | 1. **Forse inspanning**   (zoals hardlopen, zware voorwerpen tillen, inspannend sporten) | 🞎 | 🞎 | 🞎 |  |
|  | 1. **Matige inspanning**   (zoals het verplaatsen van een tafel, stofzuigen, fietsen) | 🞎 | 🞎 | 🞎 |  |
|  | 1. Tillen of boodschappentas dragen | 🞎 | 🞎 | 🞎 |  |
|  | 1. **Een paar** trappen oplopen | 🞎 | 🞎 | 🞎 |  |
|  | 1. **Eén** trap oplopen | 🞎 | 🞎 | 🞎 |  |
|  | 1. Buigen, knielen, of bukken | 🞎 | 🞎 | 🞎 |  |
|  | 1. **Meer dan een kilometer** lopen | 🞎 | 🞎 | 🞎 |  |
|  | 1. **Een halve kilometer** lopen | 🞎 | 🞎 | 🞎 |  |
|  | 1. **Honderd meter** lopen | 🞎 | 🞎 | 🞎 |  |
|  | 1. Uzelf wassen en aankleden | 🞎 | 🞎 | 🞎 |  |
|  |  |  |  |  |  |

| 1. In hoeverre heeft uw lichamelijke gezondheid of hebben uw emotionele problemen u de **afgelopen 4 weken** belemmerd in uw normale sociale bezigheden met gezin, vrienden, buren of anderen? | - helemaal niet - enigszins - nogal - veel - heel erg veel |
| --- | --- |
| 1. **Hoe vaak** hebben uw lichamelijke gezondheid of emotionele problemen gedurende de **afgelopen 4 weken** uw sociale activiteiten (zoals bezoek aan vrienden of naaste familieleden) belemmerd? | - voortdurend - meestal - soms - zelden - nooit |
| 1. Hoeveel pijn had u de **afgelopen 4 weken**? | - geen - heel licht - licht - nogal - ernstig - heel ernstig |
| 1. In welke mate heeft pijn u de **afgelopen 4 weken** belemmerd bij uw normale werkzaamheden (zowel werk buitenshuis als huishoudelijk werk)? | - helemaal niet - een klein beetje - nogal - veel - heel erg veel |

| 1. Kunt u in onderstaande tabel aangeven hoeveel uur u ongeveer, in **de afgelopen zomer en winter**,   per week aan de volgende activiteiten heeft besteed?   - *Het gaat hier om activiteiten buiten uw eventuele (betaalde) werk.* - *Vul ‘0’ in, indien een soort activiteit niet van toepassing is.* - *Rond ½-uren af naar boven (dus 1½ uur wordt 2 uur).* | | | | | |
| --- | --- | --- | --- | --- | --- |
|  | **soort activiteit in het afgelopen jaar** | | **aantal uren per week** | |  |
|  |  |  | **in de zomer** | **in de winter** |  |
|  | 1. wandelen (incl. naar werk, boodschappen en vrije tijd) | | **…** uur | **…** uur |  |
|  | 1. fietsen (incl. naar werk, boodschappen en vrije tijd) | | **…** uur | **…** uur |  |
|  | 1. tuinieren | | **…** uur | **…** uur |  |
|  | 1. klussen/doe-het-zelven | | **…** uur | **…** uur |  |
|  | 1. sport en andere lichaamsbeweging (bv. zwemmen, joggen, tennissen, dans) | | **…** uur | **…** uur |  |
|  | 1. huishoudelijk werk (bv. de was, schoonmaken, koken, zorg kinderen) | | **…** uur | **…** uur |  |
|  | | | | | |
| 1. In welke groep deelt u uw werk in het **afgelopen jaar**   in wat betreft lichaamsbeweging? | | - hoofdzakelijk zittend (bv. bureauwerk) - staand, soms lopend (bv. winkel, horeca, kappersbedrijf) - lopend met lichamelijke belasting (bv. verpleging) - zwaar lichamelijk werk (bv. schoonmaakwerk, werk op boerderij of tuinderij) - niet van toepassing (bv. pensioen of arbeidsongeschikt) | | | |

| 1. Wilt u in onderstaand schema invullen welke sport(en) u **in de loop van uw leven** hebt beoefend, of het in wedstrijdverband was,   hoeveel uur per week u aan die sport besteedde, en op welke leeftijd u dit deed?   - - - *Het gaat hier om activiteiten buiten uw eventuele (betaalde) werk.*     - *Rond ½-uren af naar boven (dus 1 ½ uur wordt 2 uur).*     - *Bij een verandering in wedstrijdniveau of het aantal uren per week dat u een bepaalde sport beoefende dient u een nieuwe regel te gebruiken.*     - *Als u een sport momenteel nog beoefent, vul dan uw huidige leeftijd in als eindleeftijd.* | | | | | | | | |
| --- | --- | --- | --- | --- | --- | --- | --- | --- |
|  |  | **sport** | **wedstrijd** | | **hoeveel uur**  **per week** | **leeftijd** | |  |
|  |  |  | **nee** | **ja** |  | **van** | **tot** |  |
|  | 1 |  | 🞎 | 🞎 | … uur | … jr | … jr |  |
|  | 2 |  | 🞎 | 🞎 | … uur | … jr | … jr |  |
|  | 3 |  | 🞎 | 🞎 | … uur | … jr | … jr |  |
|  | 4 |  | 🞎 | 🞎 | … uur | … jr | … jr |  |
|  | 5 |  | 🞎 | 🞎 | … uur | … jr | … jr |  |
|  | 6 |  | 🞎 | 🞎 | … uur | … jr | … jr |  |
|  | 7 |  | 🞎 | 🞎 | … uur | … jr | … jr |  |
|  | 8 |  | 🞎 | 🞎 | … uur | … jr | … jr |  |
|  |  |  |  |  |  |  |  |  |

| **BROERS/ ZUSSEN VOOR DE CONTROLEGROEP** | |
| --- | --- |
| 1. Heeft u één of meerdere (half)broers of (half)zussen? | - nee *🡺 ga door naar vraag O1* - ja, ik heb ….. broer(s)/ halfbroer(s) (svp aantal invullen) - ja, ik heb ….. zus(sen)/ halfzus(sen) (svp aantal invullen) |
| *Om het huidige vragenlijst-onderzoek goed te kunnen uitvoeren, is het belangrijk dat uw gegevens vergeleken worden met gegevens van personen die in het verleden niet voor kinderkanker behandeld zijn. Broers en zussen zijn daar erg geschikt voor. U heeft aangegeven dat u broers of zussen heeft. Met de volgende vraag willen we u daarom vragen of we hen mogen benaderen om ook deze vragenlijst in te vullen.* ***Het invullen van deze vraag is niet verplicht****. Bovendien, als u ons toestemming geeft om (één van) uw broers of zussen te benaderen, zullen zij vervolgens zelf kunnen besluiten of zij WEL OF NIET willen deelnemen aan dit vragenlijst-onderzoek.* | |
| 1. Geeft u ons toestemming om uw (half)broer(s) en/of (half)zus(sen) een uitnodiging te sturen voor deelname aan dit vragenlijst-onderzoek? | - ja - nee *🡺 ga door naar vraag O1* |
| De gegevens van mijn (half)broers of (half)zussen, die mogen worden benaderd, zijn:     1. Naam: …………………………………………………………..……..….….… Geslacht: m / v *     Adres: ……………………………………………………………..……..…………..………..…………    Postcode: …………………………. Woonplaats: ………............………………..….………………  Geboortedatum: …………………. Evt. emailadres/ telefoonnummer: ……..……………………     1. Naam: …………………………………………………………..……..….….… Geslacht: m / v *     Adres: ……………………………………………………………..……..…………..………..…………    Postcode: …………………………. Woonplaats: ………............………………..….………………  Geboortedatum: …………………. Evt. emailadres/ telefoonnummer: ……..……………………       1. Naam: …………………………………………………………..……..….….… Geslacht: m / v *     Adres: ……………………………………………………………..……..…………..………..…………    Postcode: …………………………. Woonplaats: ………............………………..….………………  Geboortedatum: …………………. Evt. emailadres/ telefoonnummer: ……..…………………… | |
| **TOT SLOT** | |
| 1. Heeft u de vragen in deze vragenlijst zelf ingevuld?   *Dit geldt niet voor de vragen die gaan over uw familie of geboorte.* | - ja, ik heb de vragenlijst alleen ingevuld *🡺 ga door naar vraag O9* - nee, ik heb de vragen samen met iemand anders ingevuld - nee, iemand anders heeft de vragenlijst ingevuld |
| 1. Zo nee, (met) wie was dat? | - ouder (s) - broer/ zus - mijn partner/ echtgenoot - een vriend of vriendin - verzorger/ persoonlijk begeleider - anders namelijk ……………………………………… |
| O9  Indien we naar aanleiding van deze vragenlijst nog vragen hebben, vindt u het dan goed dat we contact met u opnemen? Zo ja, kunt u dan s.v.p. uw telefoonnummer en/of uw email adres invullen? | - nee, ik wil niet dat u nog contact met mij opneemt over deze vragenlijst - ja, u mag contact met mij opnemen over deze vragenlijst   telefoonnummer ……………..………………………………..  email adres …………………………………………………….. |

| **Ruimte voor aanvullende opmerkingen** |
| --- |
