# Supplementary material for: Questionnaire‐ and linkage‐based outcomes in Dutch childhood cancer survivors: Methodology of the DCCSS LATER study part 1
Source: Cancer Med. 2022 Dec 15;12(6):7588–602. doi: 10.1002/cam4.5519 (PMC10067029; doi:10.1002/cam4.5519)
Supplement: Supplementary file 2 — Figure S1 [file CAM4-12-7588-s001.docx]

**
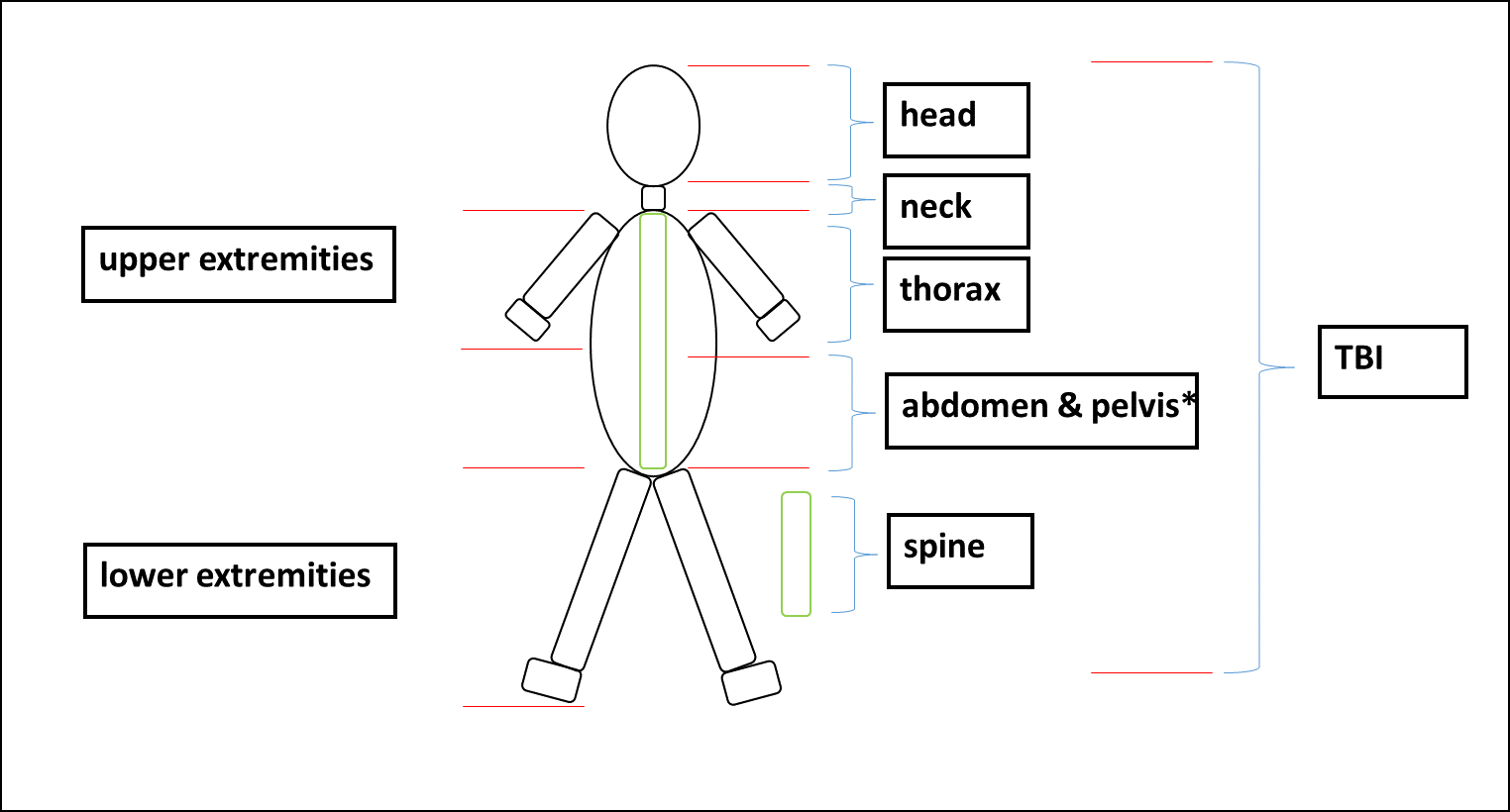
**

**Supplementary Figure 1.** Overview of different radiotherapy body compartments.

* Excluding external-beam radiotherapy on testes

Abbreviation: TBI, total body irradiation
