# Supplementary material for: Questionnaire‐ and linkage‐based outcomes in Dutch childhood cancer survivors: Methodology of the DCCSS LATER study part 1
Source: Cancer Med. 2022 Dec 15;12(6):7588–602. doi: 10.1002/cam4.5519 (PMC10067029; doi:10.1002/cam4.5519)
Supplement: Supplementary file 3 — Table S1–S3 [file CAM4-12-7588-s003.docx]

**Supplementary Tables**

| **Supplementary Table 1.** Diagnoses included in the DCCSS LATER registry, which are not covered by the third edition of the International Classification of Childhood Cancer | | |
| --- | --- | --- |
| **Diagnosis** | **ICD-O-3 morphology** | **ICD-O-3 topography** |
| Subependymoma | 9383/1 | C71._ |
| Myxopapillary ependymoma | 9394/1 | C72.0 |
| Subependymal giant cell astrocytoma | 9384/1 | C71._ |
| Pilocytic astrocytoma | 9421/1 | C71._ |
| Gliofibroma | 9442/1 | C71._ |
| Langerhans cell histiocytosis, NOS^1^ | 9751/3 |  |
| Abbreviation: ICD-O-3: International classification of diseases for oncology, third edition, first revision (2011). | | |
| ^1^ Only systemic multifocal or polyostotic Langerhans cell histiocytosis were included in the DCCSS LATER registry. | | |

| **Supplementary Table 2.** Items in the DCCSS LATER health outcomes and lifestyle questionnaire | |
| --- | --- |
| **Topic** | **Specific** |
| ***Determinants of outcomes*** |  |
| General | Weight |
|  | Height |
|  | Profession |
|  | Education |
|  | Job situation |
|  | Marital status |
|  | Living situation |
|  | Gemelli |
|  | Nationality |
|  | Country of birth |
|  | Birth weight |
|  | Gestational age |
| Family history of disease | Cancer |
|  | Cardiovascular disease |
| Reproductive and hormonal factors | Menarche details |
|  | Menopause details of mother |
|  | Pregnancy details and desire to have children |
|  | Fertility treatments |
|  | Birth control use |
|  | Hormonal treatments |
| Lifestyle factors | Smoking details |
|  | Alcohol use details |
|  | Substance use details |
|  | Physical activity details |
| ***Outcomes*** |  |
| Health problems | Myocardial infarction |
|  | Angina pectoris |
|  | Valvular disease |
|  | Pericarditis |
|  | Cardiomyopathy |
|  | Heart failure |
|  | Arrhytmia |
|  | Congenital heart defect |
|  | Other heart disease |
|  | Stroke |
|  | Transient ischemic attack |
|  | Vascular disease |
|  | Blood clotting disorder |
|  | Hypertension |
|  | Hypercholesterolemia |
|  | Gastrointestinal disorders |
|  | Pulmonary disorders |
|  | Persistent cough |
|  | Respiratory infections |
|  | Urinary tract infections |
|  | Kidney diseases |
|  | Liver diseases |
|  | Adrenal dysfunction |
|  | Musculoskeletal disorders |
|  | Diabetes |
|  | Epilepsy |
|  | Cataract |
|  | Hearing aid |
|  | Tinnitus |
|  | Growth disturbances |
|  | Hypothyroidism |
|  | Hyperthyroidism |
|  | Thyroid nodules |
|  | Other thyroid disorders |
|  | Other hormone-regulating disorders |
|  | Other disorders |
| Medications | Name medication |
|  | For what condition medication was used |
| Surgeries |  |
| Social outcomes | Problems with medical examination because of disease history |
|  | Problems with finding job |
|  | Problems with getting health insurance |
|  | Receiving any psychosocial help |
| Psychosexual outcomes | Sexual orentation |
|  | Sexual problems |
|  | Sexual activity |
| Puberty details |  |
| Mammography details |  |
| Fatigue details |  |

| **Supplementary Table 3.** Published studies with data from the DCCSS LATER 1 study | | | | | |
| --- | --- | --- | --- | --- | --- |
| **Topic** | **First author** | **Journal** | **Year** | **Methods of outcome ascertainment** | **Outcomes** |
| Cardiac | Feijen | J Clin Oncol | 2015 | Questionnaire, primary physician (GP), medical records | Heart failure |
|  | Feijen | J Am Heart Assoc. | 2019 | Questionnaire, primary physician (GP), medical records | Heart failure |
|  | Feijen | JAMA Oncol | 2019 | Questionnaire, primary physician (GP), medical records | Cardiomyopathy |
|  | Feijen | Heart | 2021 | Questionnaire, primary physician (GP), medical records | Cardiac ischemia |
| Subsequent tumors | Teepen | J Clin Oncol | 2017 | Linkages to NCR and NPR, medical records | All subsequent malignant neoplasms |
|  | Teepen | J Natl Cancer Inst | 2018 | Linkage to NPR | Colorectal adenoma and cancer |
|  | Kok | Neurooncol | 2018 | Linkage to NPR | Meningioma |
|  | Fidler | J Natl Cancer Inst | 2018 | Linkages to NCR and NPR, medical records | Bone cancers |
|  | Bright | J Natl Cancer Inst | 2018 | Linkages to NCR and NPR, medical records | Soft tissue sarcoma |
|  | Teepen | J Natl Cancer Inst | 2019 | Linkages to NCR and NPR | Skin cancer |
|  | Kok | JAMA Oncol | 2019 | Linkage to NPR | All subsequent solid benign tumors |
|  | Teepen | Cancer Causes Control | 2019 | Linkages to NCR and cause of death registry | Clinical characteristics and survival patterns of selected subsequent malignant neoplasms |
|  | Allodji | Eur J Cancer | 2019 | Linkages to NCR and NPR, medical records | Subsequent primary leukaemias |
|  | Reulen | Gut | 2020 | Linkages to NCR and NPR, medical records | Digestive cancers |
|  | Clement | Eur J Endocrinol | 2020 | Linkages to NCR and NPR, medical records | Presentation and outcome of subsequent thyroid cancer |
|  | Verbruggen | Eur J Cancer | 2021 | Linkage to NPR, medical records | Meningioma |
|  | Moskowitz | J Clin Oncol | 2021 | Linkages to NCR and NPR, medical records | Prediction of subsequent breast cancer |
|  | Wang | Eur J Cancer | 2022 | Linkages to NCR and NPR, medical records | Male breast cancer |
| Burden of disease | Streefkerk | Pediatr Blood Cancer | 2019 | Linkage to Nivel Primary Care database | Primary care physicians based health care use |
|  | Streefkerk | Plos One | 2020 | Linkage to Dutch Hospital Discharge registry | Hospitalization rates |
| Mortality | Kilsdonk | Cancer Invest | 2022 | Linkage to Central Bureau of Genealogy | Mortality |
| Fatigue | van Deuren | Cancer | 2022 | Questionnaire |  |
| Ototoxicity | Clemens | Eur J Cancer | 2016 | Audiograms | Ototoxicity |
|  | Clemens | Pediatr Hematol Oncol | 2017 | Audiograms | Course of serious hearing impairment |
|  | Meijer | Neurooncol Adv | 2020 | Questionnaire | Tinnitus |
|  | Langer | Eur J Cancer | 2020 | Audiograms | Genetic associations and diagnostic accuracy of genetic markers for platinum-induced ototoxicity |
|  | Clemens | Pharmacogenomics J | 2020 | Audiograms | Genetic associations for cisplatin-induced ototoxicity |
| Reproductive outcomes | van den Berg | Hum Reprod | 2018 | Questionnaire and clinical assessment | Ovarian reserve |
|  | van Dijk | Hum Reprod | 2018 | Questionnaire | Reproductive intentions and use of reproductive health care |
|  | van de Loo | Fertil Steril | 2019 | Questionnaire and clinical assessment | Pregnancy complications and outcomes |
|  | van Dijk | J Cancer Res Clin Oncol | 2020 | Questionnaire | Pregnancy and obstretic outcomes |
|  | Roshandel | Pediatr Blood Cancer | 2021 | Clinical assessment | Female reproductive function |
|  | van der Kooi | Hum Reprod | 2021 | Clinical assessment | Ovarian function |
| Psychosocial | van Erp | Supportive Care in Cancer | 2021 | Questionnaires | Psychosocial well-being |
|  | van Erp | Eur J Cancer | 2021 | Questionnaires | Health related quality of life |
| Abbreviations: GP, general practitioner; NCR, nationwide cancer registry; NPR, nationwide pathology registry. | | | | | |
